# Supplementary material for: Microvascular endothelial scavenger receptor class B type I protects against heart failure with preserved ejection fraction by inhibiting T-cell cardiotropism
Source: EMBO Mol Med. 2026 Apr 13;18(5):1679–706. doi: 10.1038/s44321-026-00405-9 (PMC13179366; doi:10.1038/s44321-026-00405-9)
Supplement: Supplementary file 1 — Appendix [file 44321_2026_405_MOESM1_ESM.pdf]

Appendix for

Microvascular endothelial scavenger receptor class B type I protects against heart failure with preserved ejection fraction by inhibiting T-cell cardiotropism

Table of contents

Appendix Figure S1 2
Appendix Figure S2 4
Appendix Figure S3 6
Appendix Figure S4 8
Appendix Figure S5 10
Appendix Figure S6 12
Appendix Figure S7 14
Appendix Figure S8 16
Appendix Figure S9 18
Appendix Figure S10 20
Appendix Figure S11 22
Appendix Figure S12 24
Appendix Figure S13 26
Appendix Figure S14 28
Appendix Table S1 30
Appendix Table S2 31
Appendix Table S3 32
Appendix Table S4 33

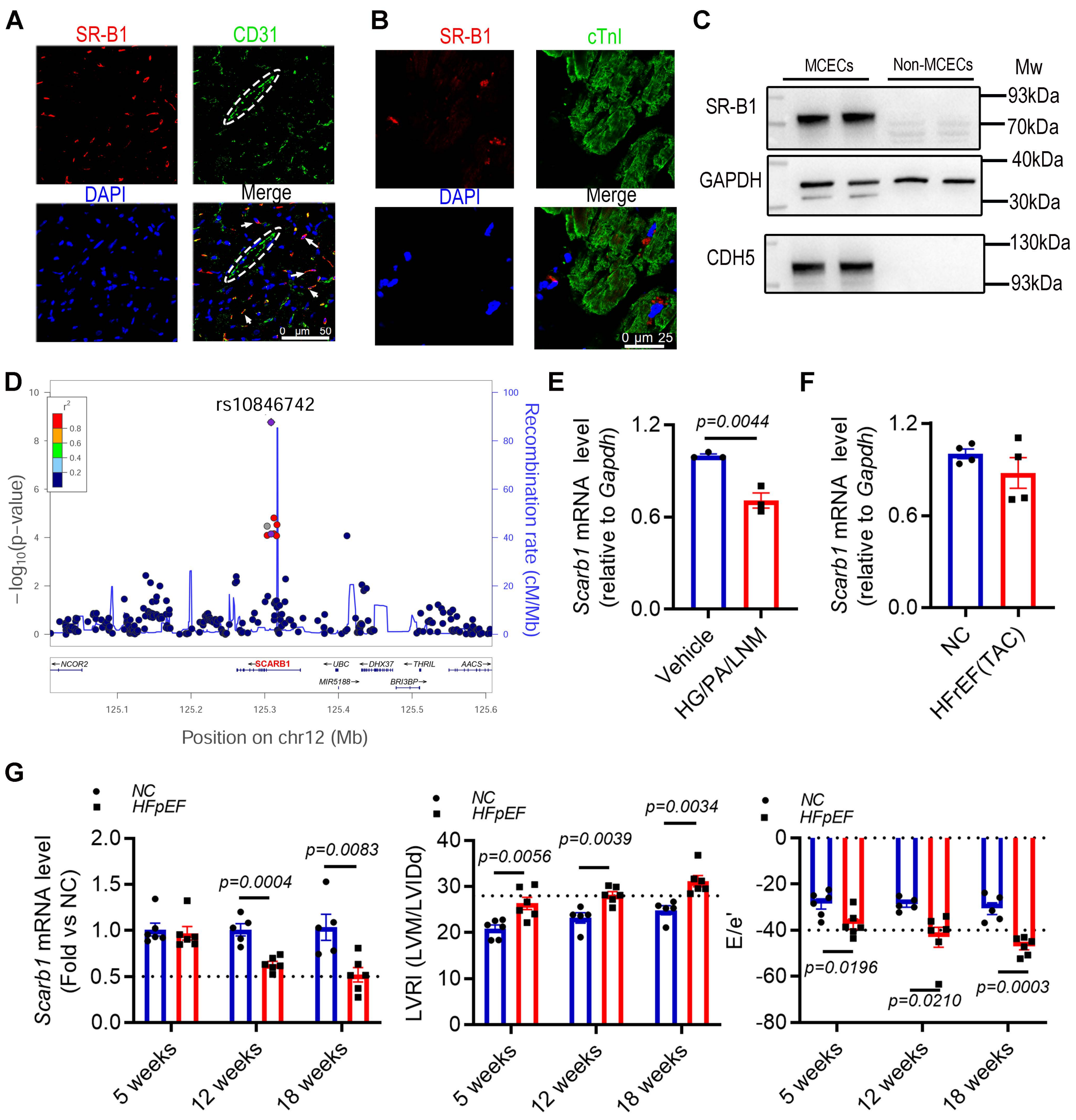
A
SR-B1
CD31
DAPI
Merge
0 µm 50
B
SR-B1
cTnI
DAPI
Merge
0 µm 25
C
MCECs
Non-MCECs
Mw
SR-B1
GAPDH
CDH5
93kDa
70kDa
40kDa
30kDa
130kDa
93kDa
D
rs10846742
−log10(p−value)
Recombination rate (cM/Mb)
Position on chr12 (Mb)
SCARB1
NCOR2
UBC
DHX37
THRIL
AACS
MIR5188
BRI3BP
E
p=0.0044
Scarb1 mRNA level (relative to Gapdh)
Vehicle
HG/PA/LNM
F
Scarb1 mRNA level (relative to Gapdh)
NC
HFrEF(TAC)
G
NC
HFpEF
Scarb1 mRNA level (Fold vs NC)
p=0.0004
p=0.0083
LVRI (LVM/LVIDd)
p=0.0056
p=0.0039
p=0.0034
E/e'
p=0.0196
p=0.0210
p=0.0003
5 weeks
12 weeks
18 weeks

Appendix Figure S1 Cardiac SR-B1 is predominantly present in cardiac endothelial cells. A, Immunofluorescent staining of SR-B1 and CD31 in mouse cardiac left ventricle. Scale bar, 50 μm. Arrow, SR-B1/CD31 co-localization. Dashed ellipse: vascular lumen. **B**, Immunofluorescent staining of SR-B1 in cardiac left ventricle of healthy human co-stained with cTnI to label cardiomyocytes. Scale bar, 25 μm. **C**, Immunoblotting of SR-B1 and CDH5 in adult mouse cardiac endothelial cells (MCECs) and non-MCECs; GAPDH abundance presented as loading control. **D**, Fine mapping of the region flanking significant locus rs10846742/*SCARB1* (*p=1.7×10-9*) with 300 kb on each side. The color code indicates linkage disequilibrium of each SNP as measured by r^2 according to the shown scale with the relevant GWAS SNP annotated. The GWAS summary statistics used were derived from the Million Veteran Program (MVP) through dbGAP under accession phs001672.v10 and Heart Failure Molecular Epidemiology for Therapeutic Targets (HERMES) in the GWAS Catalog under accession GCST009541. **E**, *Scarb1* (SR-B1) mRNA abundance was assessed in primary adult mouse cardiac endothelial cells incubated with high-glucose, palmitic acid and L-NAME treatment *via* RT-qPCR (n=3). Statistical significance was analyzed by Student's t-test. **F**, *Scarb1* (SR-B1) abundance was measured in mouse cardiac ECs from sham-operated or TAC-induced HFrEF mice (mean FS=18%) at 6 weeks post-surgery *via* RT-qPCR (n=4). **G**, *Scarb1* mRNA abundance in mouse cardiac ECs at various time points following administration of a high-fat diet (HFD) combined with L-NAME (n=5-6 / group, Welch's or Student's t-test).

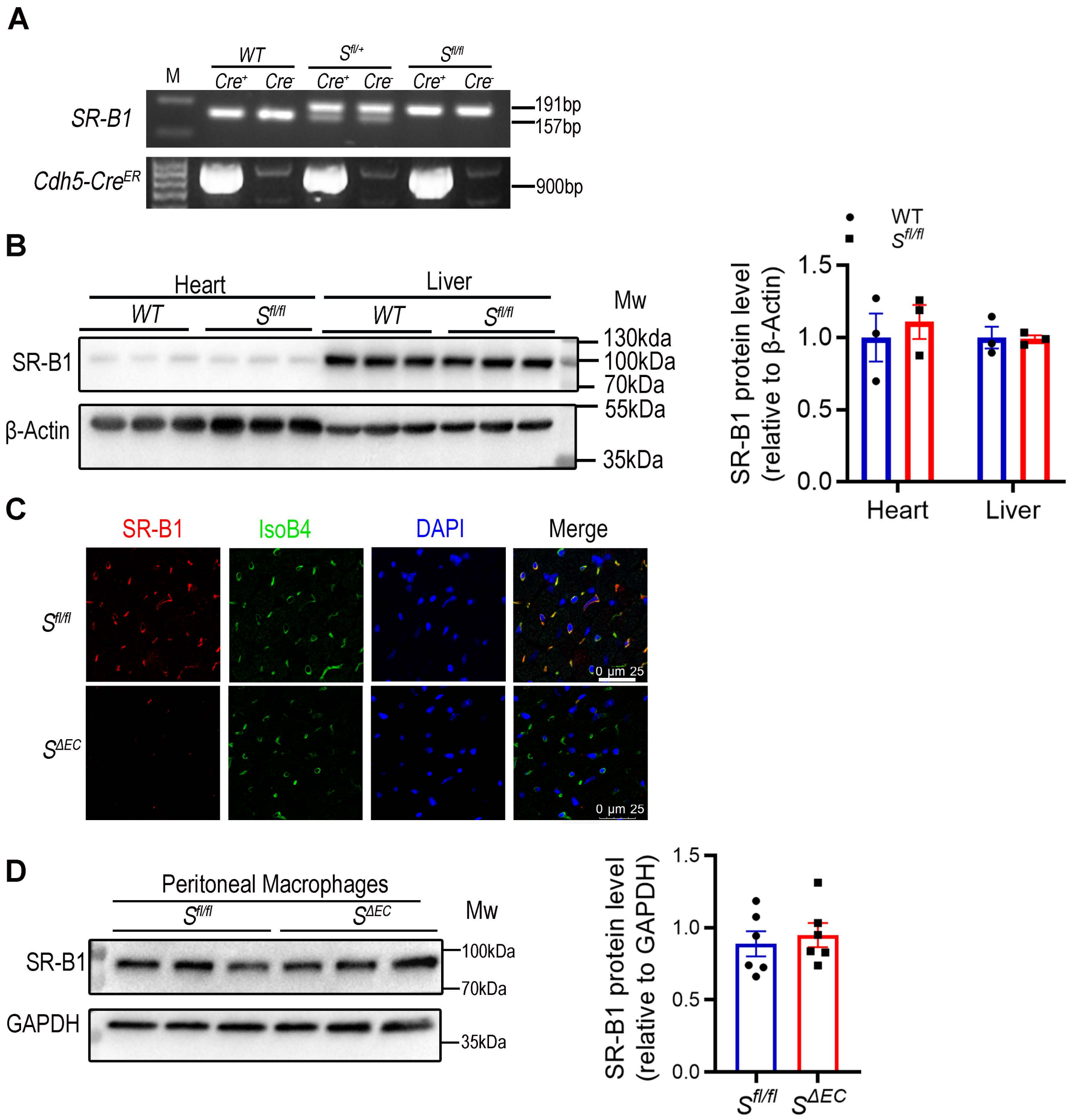
A
M
WT
Cre+ Cre-
Cre+ Cre-
Cre+ Cre-
SR-B1
191bp
157bp
Cdh5-CreER
900bp
B
Heart
Liver
WT
Sfl/fl
Mw
SR-B1
β-Actin
130kda
100kDa
70kDa
55kDa
35kDa
WT
Sfl/fl
SR-B1 protein level (relative to β-Actin)
1.5
1.0
0.5
0.0
Heart
Liver
C
SR-B1
IsoB4
DAPI
Merge
Sfl/fl
SΔEC
0 µm 25
D
Peritoneal Macrophages
Sfl/fl
SΔEC
Mw
SR-B1
GAPDH
100kDa
70kDa
35kDa
SR-B1 protein level (relative to GAPDH)
1.5
1.0
0.5
0.0
Sfl/fl
SΔEC

Appendix Figure S2 Validation of generating endothelial-specific SR-B1 deficient mice. A, A representative agarose gel image based on PCR to identify genotype of WT, $S^{fl/+}$, $S^{fl/fl}$, and *Cdh5-Cre*^{ER} (*Cre*$^{+}$) mice. **B**, Immunobloting of SR-B1 in the heart and liver of $S^{fl/fl}$ mice and their wild-type (WT) littermates (left panel); densitometric analysis of SR-B1 protein abundance (n=3, right panel). Significant difference was evaluated *via* Student's t-test. **C**, Immunofluorescent staining of SR-B1 co-stained with IsoB4 in left ventricle from $S^{fl/fl}$ and $S^{\Delta EC}$ mice 2 weeks after tamoxifen administration. Scale bar, 25 μm. **D**, Immunoblotting of SR-B1 in peritoneal macrophages isolated from $S^{fl/fl}$ and $S^{\Delta EC}$ mice 2 weeks after tamoxifen administration (left panel); densitometric analysis of SR-B1 protein level (n=6, right panel). Significant difference was evaluated *via* Student's t-test.

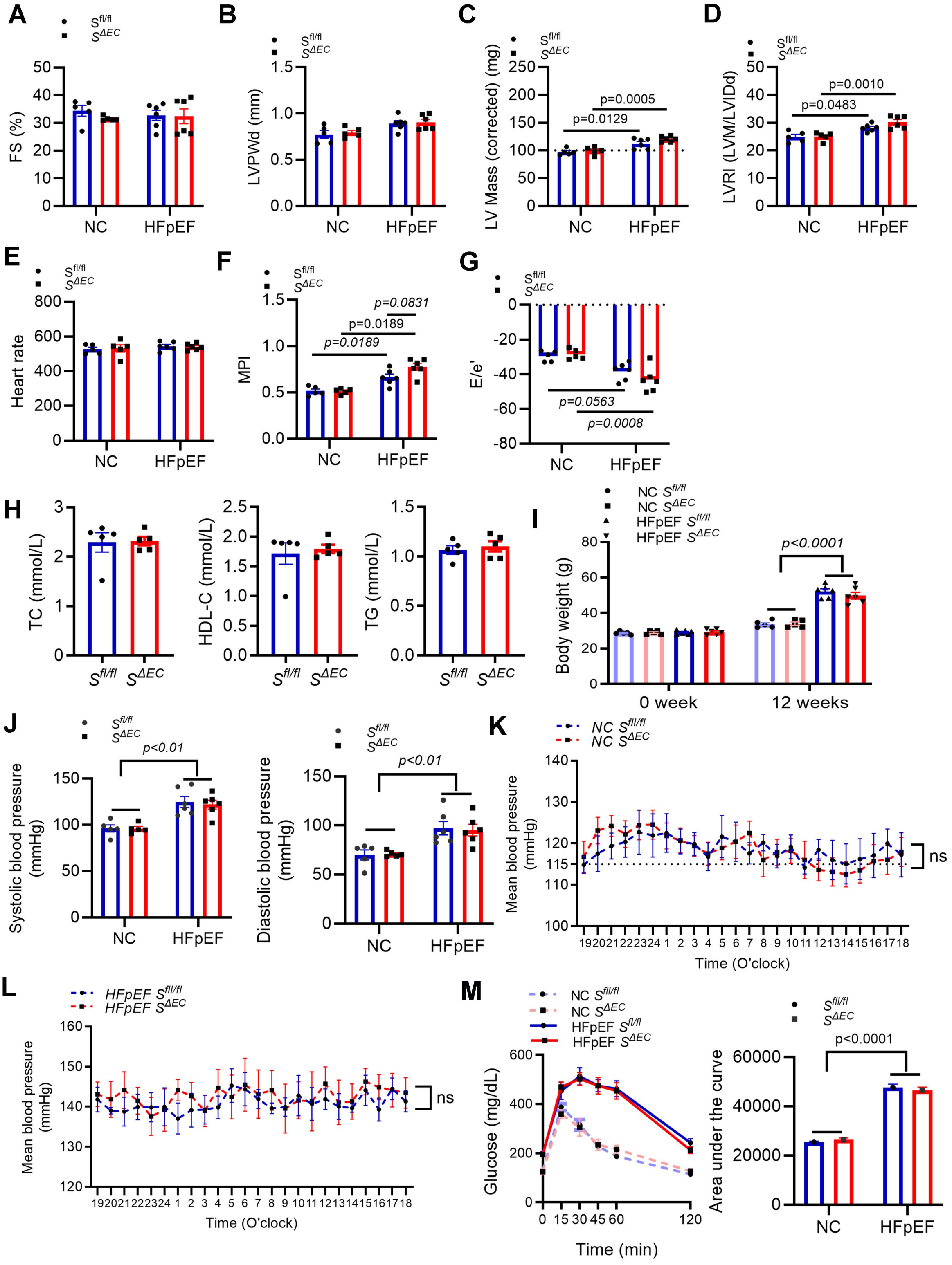
A
B
C
D
E
F
G
H
I
J
K
L
M
p=0.0129
p=0.0005
p=0.0483
p=0.0010
p=0.0189
p=0.0831
p=0.0563
p=0.0008
p<0.0001
p<0.01
ns
Time (O'clock)
Time (min)
Area under the curve
NC
HFpEF
0 week
12 weeks

Appendix Figure S3 Endothelial-limited SR-B1 deficient mice has a detrimental trend of cardiac hypertrophy and diastolic dysfunction after 10 weeks of HFD and L-NAME regimens. **A**, FS, left ventricular fractional shortening. **B**, LVPWd, left ventricular posterior wall diastolic thickness. **C**, LV mass, left ventricular mass. **D**, LVRI, left ventricular remodeling index. **E**, Heart rate in short axis view. **F**, MPI, myocardium performance index. **G**, E/e′, ratio between mitral E wave and e′ wave. N = 5, 5, 6, 6; statistical difference was assessed by two-way ANOVA followed by Bonferroni post-hoc test (**A-G**). Endothelial-specific SR-B1 deletion does not alter basic characteristics of mice (**H-M**). **H**, Plasma concentrations of total cholesterol (TC, left, Mann-Whitney test), high-density lipoprotein cholesterol (HDL-C, middle, Mann-Whitney test), and triglyceride (TG, right, Student's t-test) were measured in 10-week-old $S^{fl/fl}$ and $S^{\Delta EC}$ mice. **I**, Body weight of $S^{fl/fl}$ and $S^{\Delta EC}$ mice before HFpEF diet and after 12 weeks of normal or HFpEF diets (n=5, 5, 6, 6, two-way ANOVA followed by Bonferroni post-hoc test). **J**, Systolic and diastolic blood pressure in $S^{fl/fl}$ and $S^{\Delta EC}$ mice after 12 weeks of normal or HFpEF diet (n=5, 5, 6, 6, two-way ANOVA followed by Bonferroni post-hoc test). **K**, The 24-hour blood pressure in 10-week-old $S^{fl/fl}$ and $S^{\Delta EC}$ healthy mice was monitored by radiotelemetry at 3 weeks following tamoxifen injection (n = 5, 6). Longitudinal blood pressure data were analyzed using a mixed-effects model with repeated measures. **L**, The 24-hour blood pressure profiles of $S^{fl/fl}$ and $S^{\Delta EC}$ HFpEF mice, which underwent a 20-week regimen of high-fat diet (HFD) combined with L-NAME treatment, were monitored using radiotelemetry at 3 weeks post-tamoxifen injection (n = 4; a mixed-effects model with repeated measures). **M**, Blood glucose during intraperitoneal glucose tolerance test (ipGTT) in $S^{fl/fl}$ and $S^{\Delta EC}$ mice after 12 weeks of normal or 60% HFD diet plus L-NAME (left panel), with corresponding area under the curve (AUC) (right panel, n=5, 5, 6, 6, two-way ANOVA followed by Bonferroni post-hoc test).

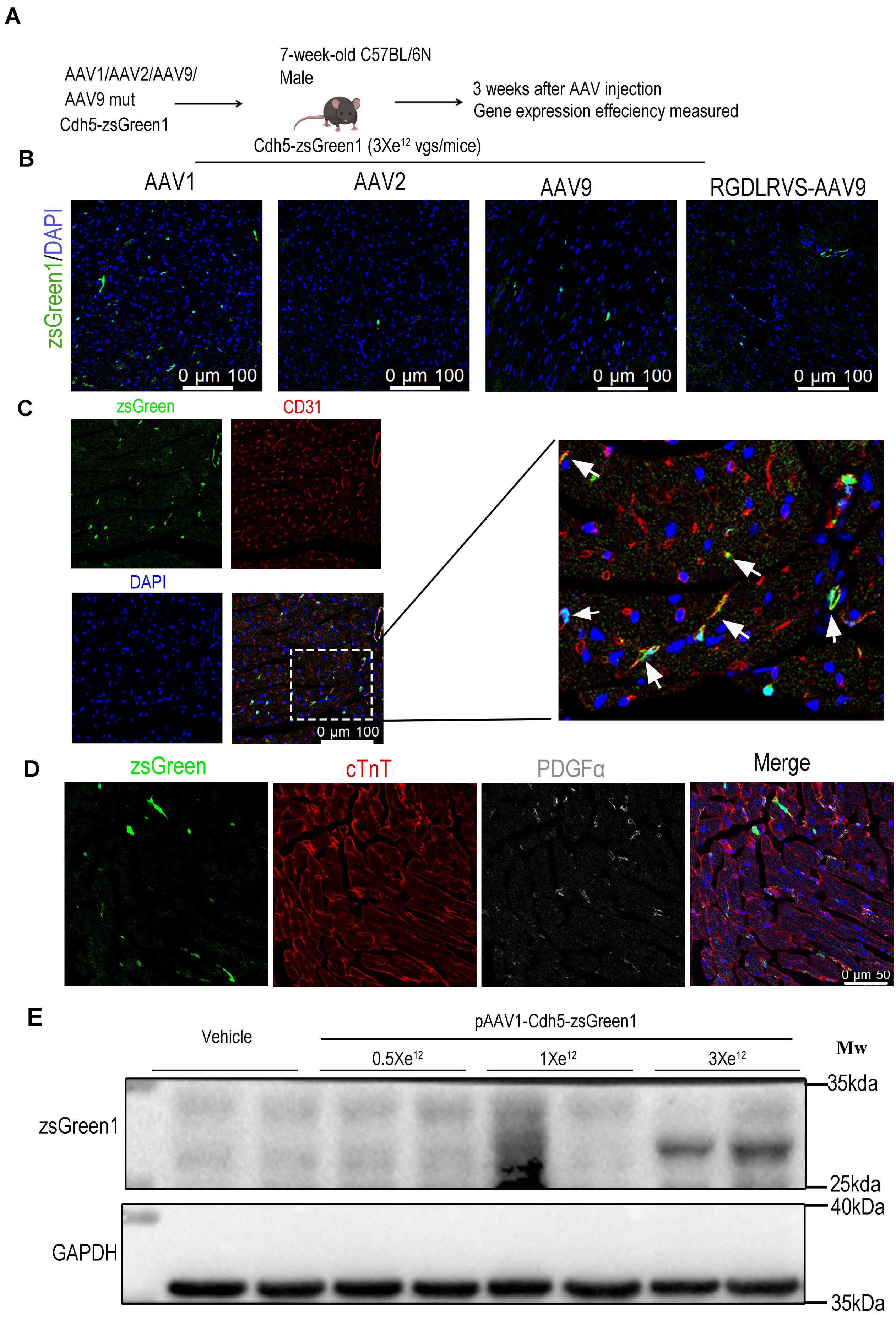
A
AAV1/AAV2/AAV9/
AAV9 mut
Cdh5-zsGreen1
7-week-old C57BL/6N
Male
3 weeks after AAV injection
Gene expression effeciency measured
Cdh5-zsGreen1 (3Xe12 vgs/mice)
B
AAV1
AAV2
AAV9
RGDLRVS-AAV9
zsGreen1/DAPI
0 μm 100
C
zsGreen
CD31
DAPI
0 μm 100
D
zsGreen
cTnT
PDGFα
Merge
0 μm 50
E
Vehicle
pAAV1-Cdh5-zsGreen1
0.5Xe12
1Xe12
3Xe12
Mw
zsGreen1
GAPDH
35kda
25kda
40kDa
35kDa

Appendix Figure S4 Screening and validating for high-efficiency serotypes and concentration of AAV in invading cardiac endothelial cells. **A**, A schematic diagram of experiment workflow to investigate the efficiency of AAV invading cardiac endothelial cells. **B**, Fluorescent imaging of ZsGreen co-staining with DAPI in the cardiac ventricles of wild-type mice after injected with different AAV serotypes. Scale bar, 100 μm. **C**, Representative fluorescence images showing ZsGreen signals mainly localized in CD31-positive cells in cardiac ventricular sections of wild-type (WT) mice following AAV1-mediated gene delivery. Scale bar, 100 μm. Arrow, CD31+ endothelial cells expressing ZsGreen. **D**, Representative fluorescence microscopy images, showing signals for ZsGreen, cardiac troponin T (cTnT), and platelet-derived growth factor receptor alpha (PDGFα) after AAV1-mediated gene delivery. Scale bar, 50 μm. **E**, Immunoblotting of ZsGreen and GAPDH in the cardiac ventricles of wild-type mice after injected with different doses of AAV1.

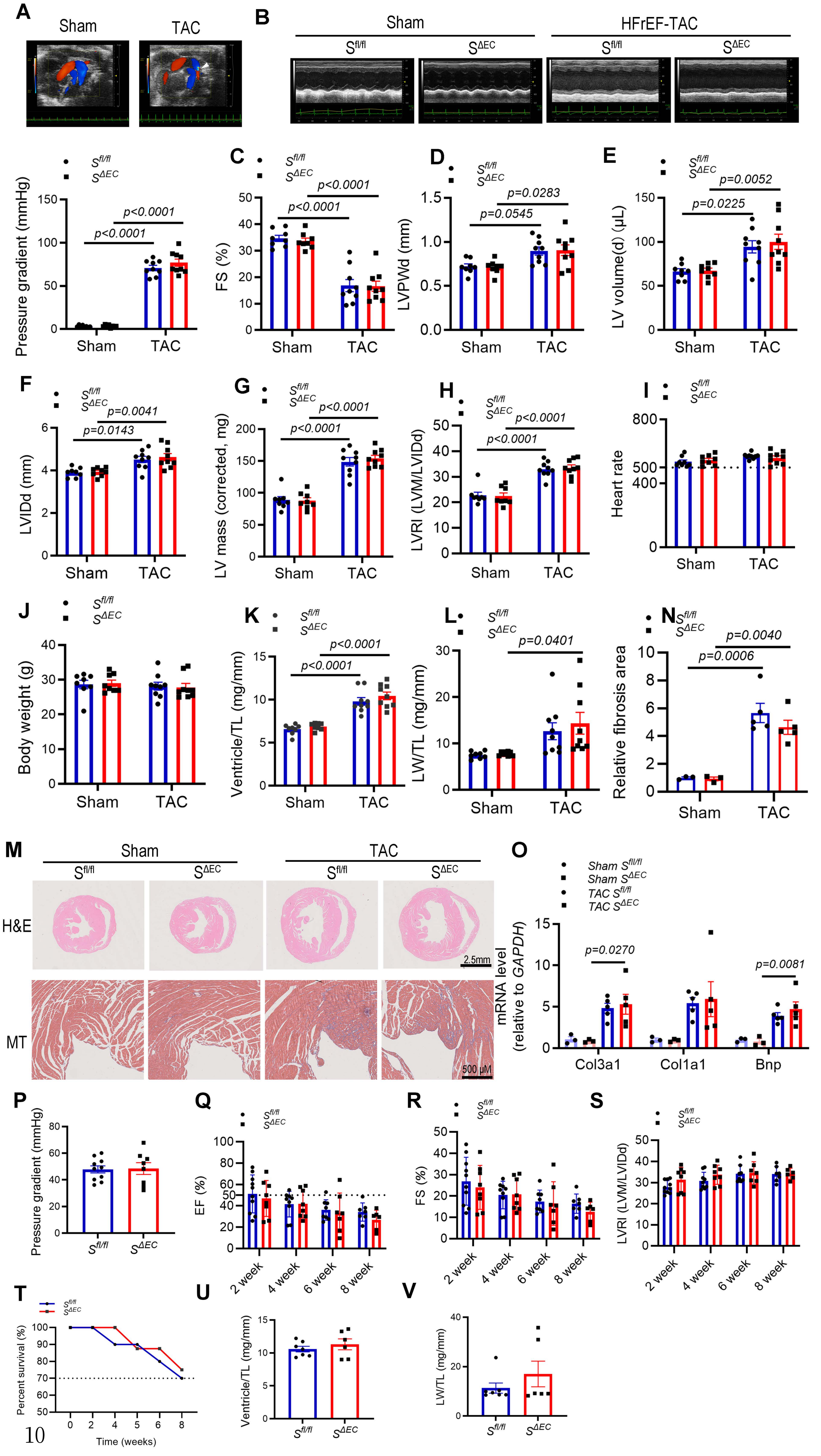
A
Sham
TAC
B
Sham
HFrEF-TAC
S^fl/fl
S^ΔEC
Pressure gradient (mmHg)
p<0.0001
Sham
TAC
C
FS (%)
D
LVPWd (mm)
p=0.0283
p=0.0545
E
LV volume(d) (μL)
p=0.0052
p=0.0225
F
LVIDd (mm)
p=0.0041
p=0.0143
G
LV mass (corrected, mg)
H
LVRI (LVM/LVIDd)
I
Heart rate
J
Body weight (g)
K
Ventricle/TL (mg/mm)
L
LW/TL (mg/mm)
p=0.0401
N
Relative fibrosis area
p=0.0040
p=0.0006
M
H&E
MT
2.5mm
500 μM
O
Sham S^fl/fl
Sham S^ΔEC
TAC S^fl/fl
TAC S^ΔEC
mRNA level (relative to GAPDH)
p=0.0270
p=0.0081
Col3a1
Col1a1
Bnp
P
Q
EF (%)
R
S
2 week
4 week
6 week
8 week
T
Percent survival (%)
Time (weeks)
U
V

Appendix Figure S5 Endothelial-specific SR-B1 is dispensable for acute cardiac systolic dysfunction and pathological remodeling induced by TAC. All cardiac function index parameters obtained from echocardiography were in mice 2 weeks after TAC operation (**A-L**, n=8,8,9,9). **A**, Representative color Doppler images of aortic arch in sham and TAC mice (upper, the white arrow indicates the ligation site); pressure gradient across the constriction site was calculated according to the modified Bernoulli equation 4 x $Vmax^2$, and Vmax is the maximum peak aortic velocity obtained from Pulsed-wave Doppler imaging in mice 1 week after TAC operation (lower). **B**, Representative M-mode images in the parasternal short axis view. **C**, FS. **D**, LVPWd. **E**, LV volume (d), Left ventricular volume at the end of diastole. **F**, LVIDd. **G**, LV mass. **H**, LVRI. **I**, Heart rate in parasternal short axis view. **J**, Body weight. **K**, Ratio between cardiac ventricle weight and tibia length (TL). **L**, Ratio between wet lung weight and TL in $S^{fl/fl}$ and $S^{\Delta EC}$ mice euthanized 2 weeks after TAC operation (n=8,8,9,9). **M**, Representative images of Hematoxylin and Eosin (H&E; Scale bar, 2.5 mm) and Masson's Trichrome (MT; Scale bar, 500 µm.) staining in transversal sections of heart 2 weeks after TAC operation. **N**, Fibrosis area in MT-stained transversal sections was measured and compared relative to $S^{fl/fl}$ mice subjected to sham operation (n=3, 3, 5, 5). **O**, *Col3a1*, *Col1a1*, and *Bnp* mRNA abundance in left cardiac ventricle was measured *via* RT-qPCR (n=3, 3, 5, 5). A two-way ANOVA followed by Bonferroni post-hoc test was applied to evaluate the statistical difference. **P-Q**, Cardiac functional dynamics after TAC surgery was assessed (n=8-10, Unpaired Student's t-test or Mann-Whitney test). **P**, Pressure gradient across aortic constriction 1 week post-TAC. **Q**, EF. **R**, FS. **S**, LVRI. **T**, Percent survival after TAC surgery. **U**, Ratio between cardiac ventricle weight and tibia length (TL) in $S^{fl/fl}$ and $S^{\Delta EC}$ mice euthanized 8 weeks after TAC surgery. **V**, Ratio between wet lung weight and TL.

A Gating strategy for the EnMT data

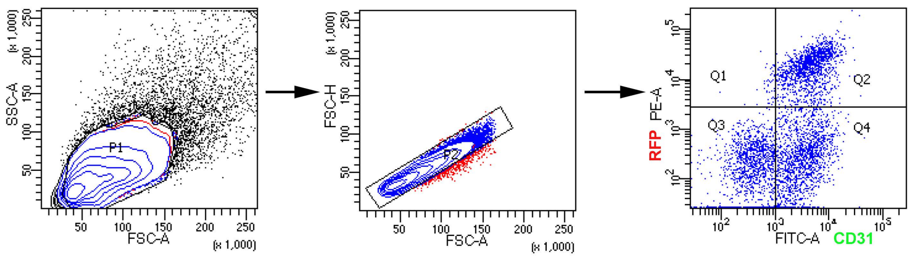

B Gating strategy for flow cytometric analyses of immune cell in heart

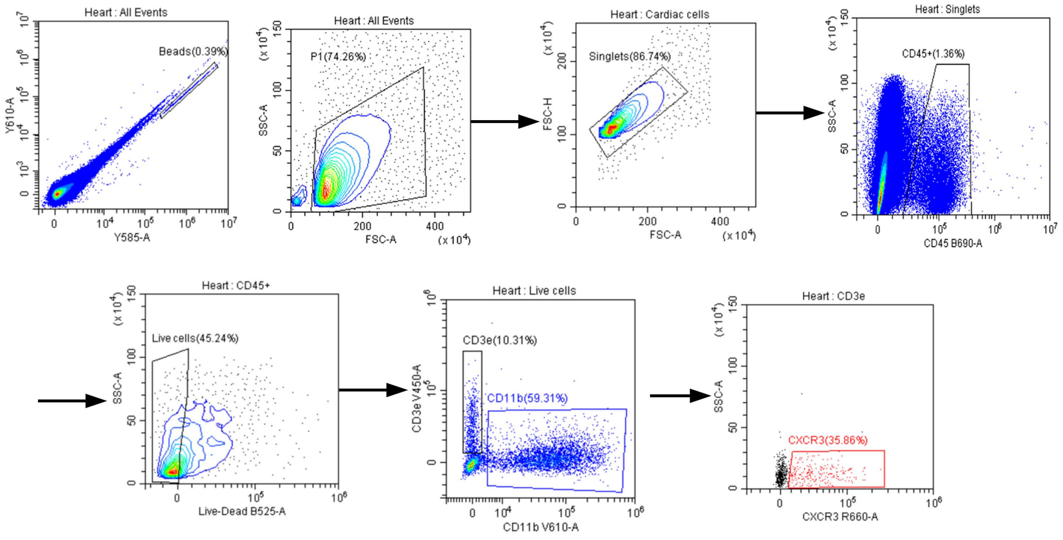

C Gating strategy for flow cytometric analyses of immune cell in blood

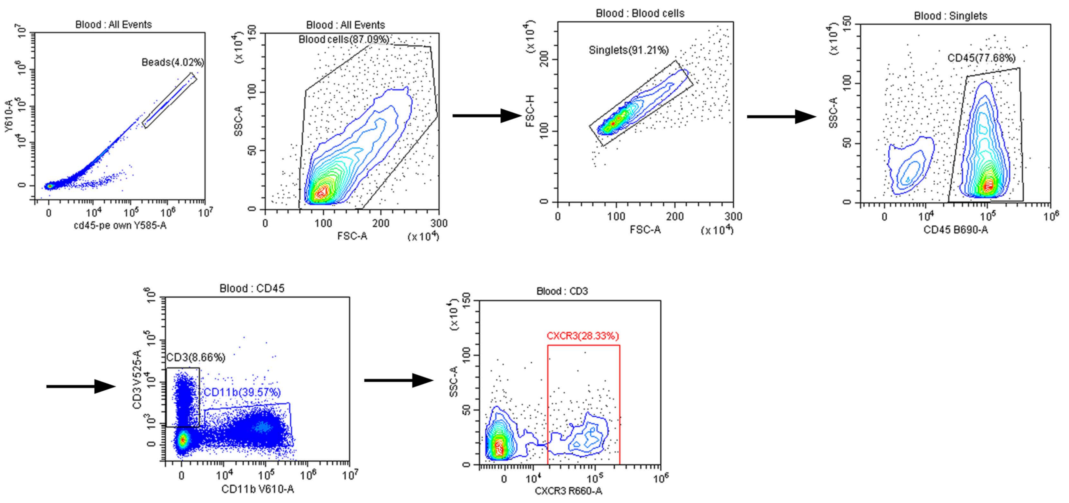

Appendix Figure S6 Gating strategies of flow cytometry. A, Gating strategy for flow cytometry of EnMT. Red fluorescence protein (RFP) from cardiac endothelial ell was detected with PE channel (*y* axis), and endothelial marker CD31 detected with FITC channel (*x* axis). **B-C**, Flow cytometry gating strategy for characterizing cardiac (**B**) and circulating (**C**) immune cells in mice. Sequential gating was performed to identify distinct immune cell populations based on specific surface marker expression profiles.

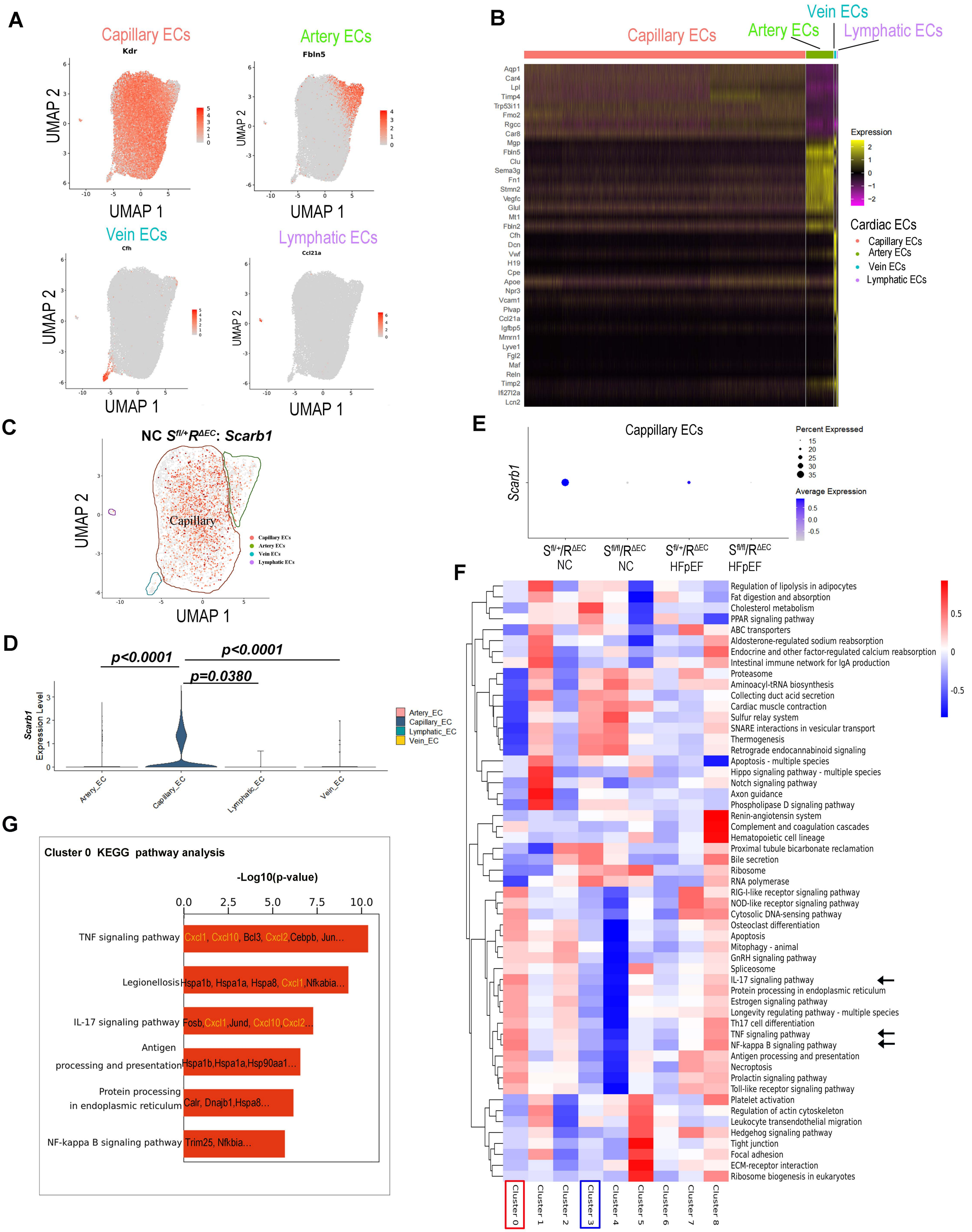
A
Capillary ECs
Artery ECs
Vein ECs
Lymphatic ECs
UMAP 1
UMAP 2
B
Cardiac ECs
Expression
C
NC $S^{fl/+}R^{\Delta EC}$: Scarb1
Capillary
D
p<0.0001
p<0.0001
p=0.0380
Scarb1 Expression Level
Artery_EC
Capillary_EC
Lymphatic_EC
Vein_EC
E
Cappillary ECs
Percent Expressed
Average Expression
$S^{fl/+}/R^{\Delta EC}$ NC
$S^{fl/fl}/R^{\Delta EC}$ NC
$S^{fl/+}/R^{\Delta EC}$ HFpEF
$S^{fl/fl}/R^{\Delta EC}$ HFpEF
F
Regulation of lipolysis in adipocytes
Fat digestion and absorption
Cholesterol metabolism
PPAR signaling pathway
ABC transporters
Aldosterone-regulated sodium reabsorption
Endocrine and other factor-regulated calcium reabsorption
Intestinal immune network for IgA production
Proteasome
Aminoacyl-tRNA biosynthesis
Collecting duct acid secretion
Cardiac muscle contraction
Sulfur relay system
SNARE interactions in vesicular transport
Thermogenesis
Retrograde endocannabinoid signaling
Apoptosis - multiple species
Hippo signaling pathway - multiple species
Notch signaling pathway
Axon guidance
Phospholipase D signaling pathway
Renin-angiotensin system
Complement and coagulation cascades
Hematopoietic cell lineage
Proximal tubule bicarbonate reclamation
Bile secretion
Ribosome
RNA polymerase
RIG-I-like receptor signaling pathway
NOD-like receptor signaling pathway
Cytosolic DNA-sensing pathway
Osteoclast differentiation
Apoptosis
Mitophagy - animal
GnRH signaling pathway
Spliceosome
IL-17 signaling pathway
Protein processing in endoplasmic reticulum
Estrogen signaling pathway
Longevity regulating pathway - multiple species
Th17 cell differentiation
TNF signaling pathway
NF-kappa B signaling pathway
Antigen processing and presentation
Necroptosis
Prolactin signaling pathway
Toll-like receptor signaling pathway
Platelet activation
Regulation of actin cytoskeleton
Leukocyte transendothelial migration
Hedgehog signaling pathway
Tight junction
Focal adhesion
ECM-receptor interaction
Ribosome biogenesis in eukaryotes
Cluster 0
Cluster 1
Cluster 2
Cluster 3
Cluster 4
Cluster 5
Cluster 6
Cluster 7
Cluster 8
G
Cluster 0 KEGG pathway analysis
-Log10(p-value)
TNF signaling pathway: Cxcl1, Cxcl10, Bcl3, Cxcl2,Cebpb, Jun...
Legionellosis: Hspa1b, Hspa1a, Hspa8, Cxcl1,Nfkabia...
IL-17 signaling pathway: Fosb,Cxcl1,Jund, Cxcl10,Cxcl2...
Antigen processing and presentation: Hspa1b,Hspa1a,Hsp90aa1...
Protein processing in endoplasmic reticulum: Calr, Dnajb1,Hspa8...
NF-kappa B signaling pathway: Trim25, Nfkbia...

Appendix Figure S7 Definition of cardiac endothelial clusters. **A**, UMAP plot, showing the expression of representative markers from four cluster, including the capillary ECs, artery ECs, vein ECs, and lymphatic ECs. **B**, Heatmap top 10-ranking marker genes for each cluster of cardiac ECs. Yellow represents high gene expression and purple represents low gene expression. **C**, UMAP visualization depicting *Scarb1* (SR-B1) expression patterns across four EC clusters from the NC $S^{fl/+}$ $RFP^{\Delta EC}$ group. **D**, Quantitative analysis of SR-B1 expression levels in four EC clusters from the NC $S^{fl/+}$ $RFP^{\Delta EC}$ control group. Mann-Whitney U test was used to evaluate the statistical difference. **E**, The bubble map of *Scarb1* expression in capillary ECs from four groups. **F**, Heatmap showing KEGG signaling pathways enriched in sub-clusters of cardiac microvascular ECs. **G**, Bar chart of the top 10 differentially enriched KEGG pathway in the capillary subcluster 0.

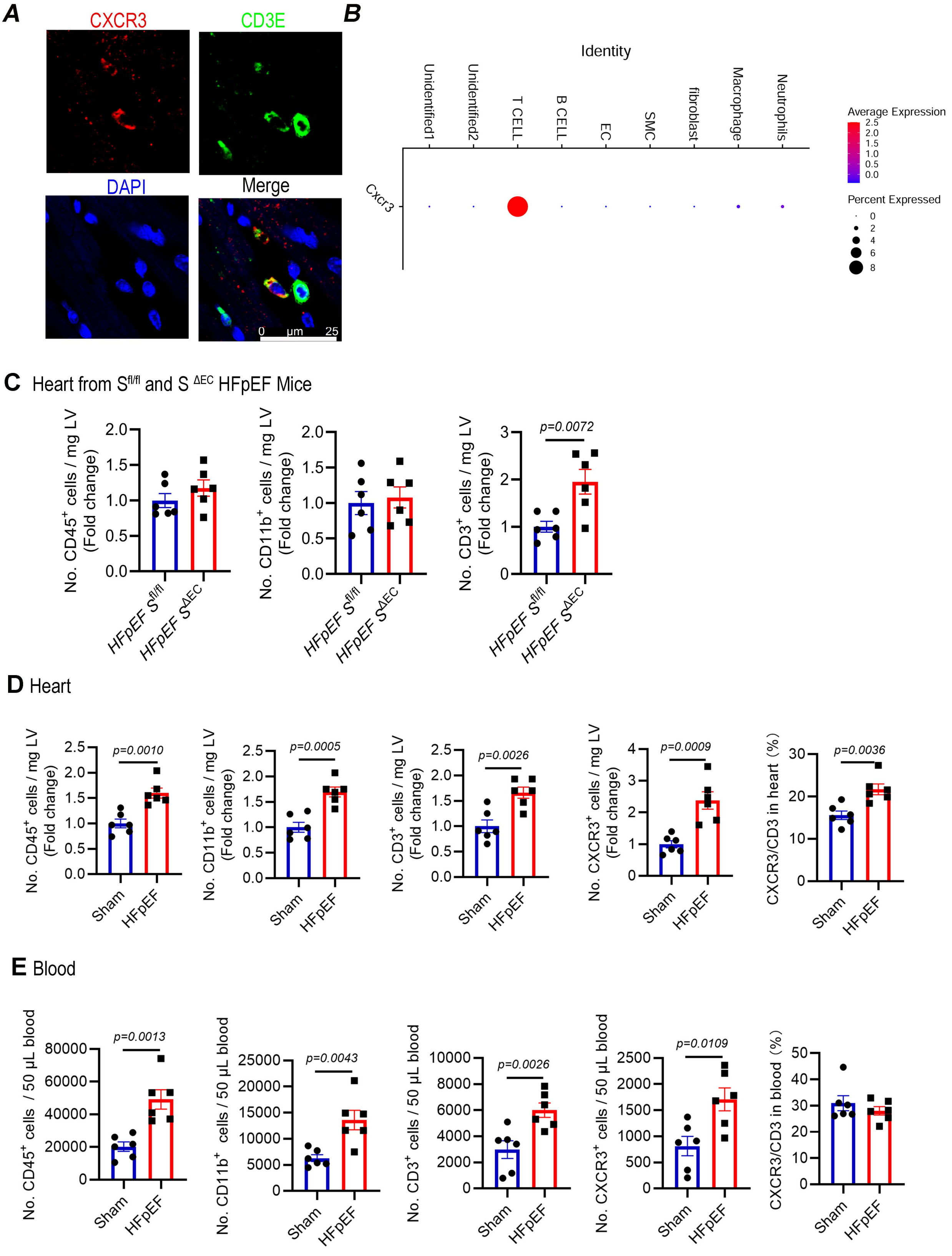
A
CXCR3
CD3E
DAPI
Merge
0 μm 25
B
Identity
Unidentified1
Unidentified2
T CELL
B CELL
EC
SMC
fibroblast
Macrophage
Neutrophils
Cxcr3
Average Expression
Percent Expressed
C Heart from S^fl/fl and S^ΔEC HFpEF Mice
No. CD45+ cells / mg LV (Fold change)
No. CD11b+ cells / mg LV (Fold change)
No. CD3+ cells / mg LV (Fold change)
p=0.0072
HFpEF S^fl/fl
HFpEF S^ΔEC
D Heart
p=0.0010
p=0.0005
p=0.0026
p=0.0009
p=0.0036
No. CXCR3+ cells / mg LV (Fold change)
CXCR3/CD3 in heart (%)
Sham
HFpEF
E Blood
No. CD45+ cells / 50 μL blood
No. CD11b+ cells / 50 μL blood
No. CD3+ cells / 50 μL blood
No. CXCR3+ cells / 50 μL blood
CXCR3/CD3 in blood (%)
p=0.0013
p=0.0043
p=0.0026
p=0.0109

Appendix Figure S8 A, Immunofluorescent staining of CXCR3 in HFpEF cardiac ventricle sections co-stained with CD3e to label T cells and DAPI to label nuclei. Scale bar, 25 μm. **B**, The bubble map illustrates *Cxcr3* expression in a mixed cardiac sample, including healthy control mice and HFpEF mice, derived from a wild-type single-cell transcriptome database (GSE236586). **C**, Quantitative flow cytometric analysis of immune cell populations, including CD45+ leukocytes, CD11b+ myeloid cells, and CD3+ T lymphocytes, in the left ventricular tissue of $S^{fl/fl}$ and $S^{\Delta EC}$ HFpEF mice (n=6, Student's t-test). **D**, Flow cytometric analysis of immune cell infiltration in cardiac tissues from mice subjected to a 10-week HFD diet plus L-NAME compared with control group (n = 6, Student's t-test). **E**, Absolute immune cell counts by flow cytometry in peripheral blood comparing 10-week HFpEF mice with control mice. (n=6, Student's t-test or Mann-Whitney test).

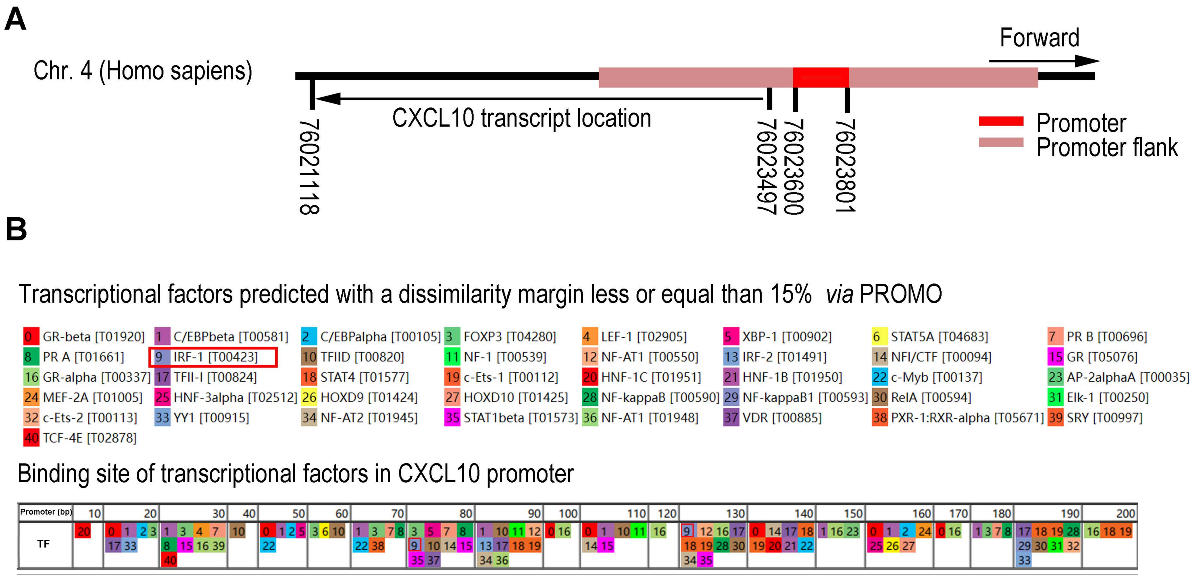
A
Chr. 4 (Homo sapiens)
Forward
CXCL10 transcript location
76021118
76023497
76023600
76023801
Promoter
Promoter flank
B
Transcriptional factors predicted with a dissimilarity margin less or equal than 15% via PROMO
0 GR-beta [T01920]
1 C/EBPbeta [T00581]
2 C/EBPalpha [T00105]
3 FOXP3 [T04280]
4 LEF-1 [T02905]
5 XBP-1 [T00902]
6 STAT5A [T04683]
7 PR B [T00696]
8 PR A [T01661]
9 IRF-1 [T00423]
10 TFIID [T00820]
11 NF-1 [T00539]
12 NF-AT1 [T00550]
13 IRF-2 [T01491]
14 NFI/CTF [T00094]
15 GR [T05076]
16 GR-alpha [T00337]
17 TFII-I [T00824]
18 STAT4 [T01577]
19 c-Ets-1 [T00112]
20 HNF-1C [T01951]
21 HNF-1B [T01950]
22 c-Myb [T00137]
23 AP-2alphaA [T00035]
24 MEF-2A [T01005]
25 HNF-3alpha [T02512]
26 HOXD9 [T01424]
27 HOXD10 [T01425]
28 NF-kappaB [T00590]
29 NF-kappaB1 [T00593]
30 RelA [T00594]
31 Elk-1 [T00250]
32 c-Ets-2 [T00113]
33 YY1 [T00915]
34 NF-AT2 [T01945]
35 STAT1beta [T01573]
36 NF-AT1 [T01948]
37 VDR [T00885]
38 PXR-1:RXR-alpha [T05671]
39 SRY [T00997]
40 TCF-4E [T02878]
Binding site of transcriptional factors in CXCL10 promoter
Promoter (bp)
TF

Appendix Figure S9 Computational prediction of transcription factors binding to the CXCL10 promoter. A, *CXCL10* promoter location obtained from Ensembl genome browser 94 (http://www.ensembl.org). **B**, Rank of predicted transcriptional factors (top panel) and corresponding binding sites (bottom panel) using the PROMO algorithm[14,15].

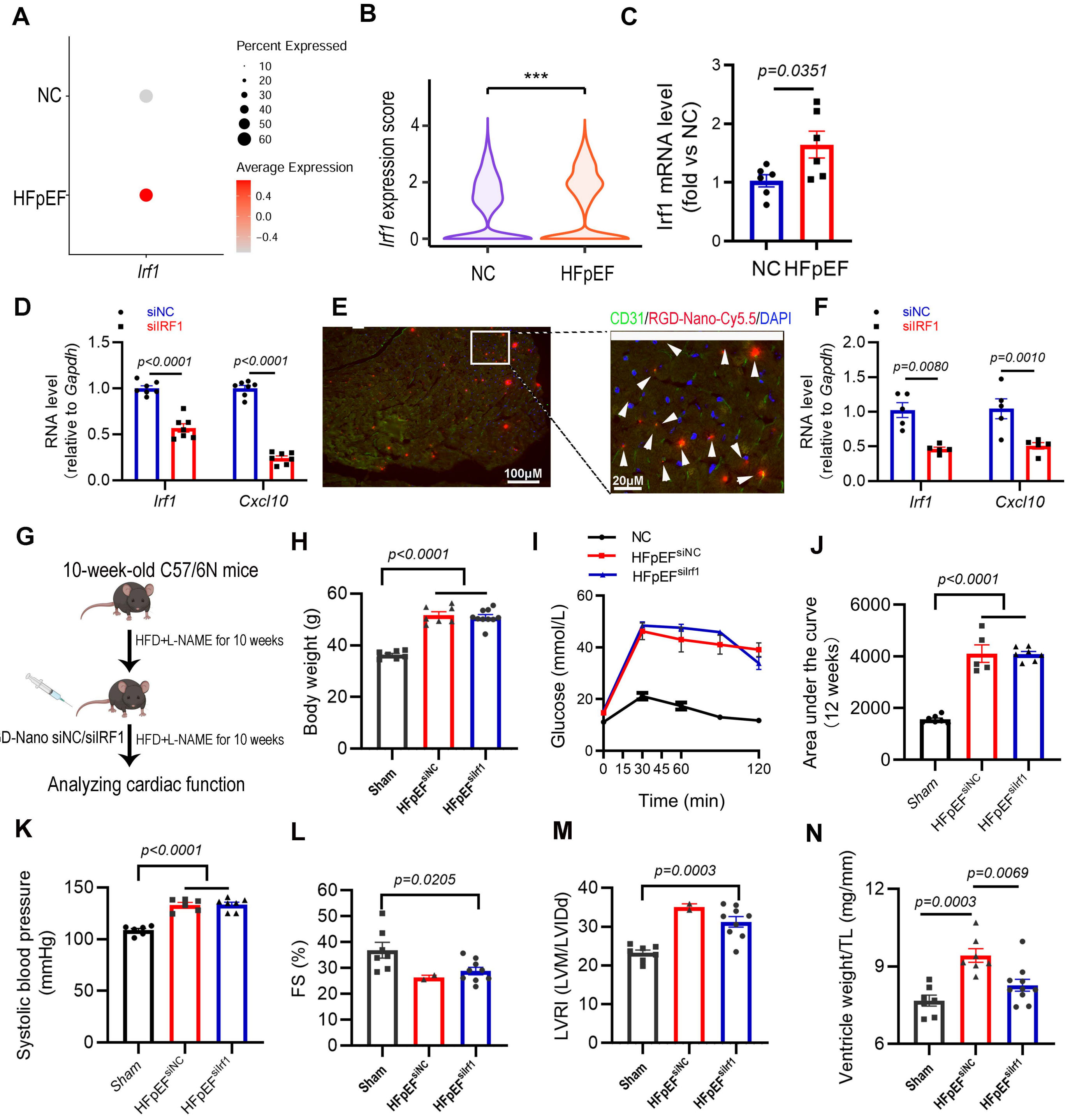
A
Percent Expressed
10
20
30
40
50
60
Average Expression
0.4
0.0
−0.4
NC
HFpEF
Irf1
B
***
Irf1 expression score
NC
HFpEF
C
p=0.0351
Irf1 mRNA level (fold vs NC)
NC
HFpEF
D
siNC
siIRF1
RNA level (relative to Gapdh)
p<0.0001
p<0.0001
Irf1
Cxcl10
E
CD31/RGD-Nano-Cy5.5/DAPI
100μM
20μM
F
siNC
siIRF1
RNA level (relative to Gapdh)
p=0.0080
p=0.0010
Irf1
Cxcl10
G
10-week-old C57/6N mice
HFD+L-NAME for 10 weeks
RGD-Nano siNC/siIRF1
HFD+L-NAME for 10 weeks
Analyzing cardiac function
H
p<0.0001
Body weight (g)
Sham
HFpEF siNC
HFpEF siIrf1
I
NC
HFpEF siNC
HFpEF siIrf1
Glucose (mmol/L)
Time (min)
J
p<0.0001
Area under the curve (12 weeks)
Sham
HFpEF siNC
HFpEF siIrf1
K
p<0.0001
Systolic blood pressure (mmHg)
Sham
HFpEF siNC
HFpEF siIrf1
L
p=0.0205
FS (%)
Sham
HFpEF siNC
HFpEF siIrf1
M
p=0.0003
LVRI (LVM/LVIDd)
Sham
HFpEF siNC
HFpEF siIrf1
N
p=0.0003
p=0.0069
Ventricle weight/TL (mg/mm)
Sham
HFpEF siNC
HFpEF siIrf1

Appendix Figure S10 Knockdown of IRF1 specifically in cardiac endothelial cells significantly ameliorates cardiac pathological remodeling in HFpEF mice. **A**, The bubble map of *Irf1* expression in cardiac capillary endothelial cells from HFpEF and NC groups. **B**, Quantitative analysis of *Irf1* expression levels from the HFpEF and control groups. Mann-Whitney U test was used to evaluate the statistical difference. **C**, Irf1 abundance was measured in mouse cardiac ECs from normal control and HFpEF mice at 20 weeks of HFD plus L-NAME regimens (n=6). **D**, Quantitative analysis of *Irf1* and *Cxcl10* mRNA expression levels in mouse endothelial cell line H5V following transfection with either scramble control siRNA (siNC) or IRF1-specific siRNA (siIRF1) (n=7, Student's t-test). **E**, Representative immunofluorescent images demonstrating co-localization (indicated by white arrows) of CD31+ endothelial cells (green) and Cy5.5-labeled RGD peptide-conjugated magnetic nanoparticles (red) in cardiac tissue sections. Nuclei were counterstained with DAPI (blue). Scale bar: 100 μm (left panel); 20 μm (right panel). **F**, Targeted delivery of IRF1-siRNA using RGD-conjugated nanoparticles significantly reduces *Irf1* and *Cxcl10* mRNA expression in isolated cardiac endothelial cells from wild-type mice, as quantified by RT-qPCR (n=5; Student's t-test). **G**, Workflow of the experimental design to investigate the role of endothelial-specific IRF1 in the HFpEF pathogenesis. **H**, Body weight (n=7, 7, 10). **I**, Blood glucose during intraperitoneal glucose tolerance test (ipGTT) (n=7, 7, 10), with corresponding area under the curve (**J**). **K**, Systolic blood pressure (n=6, 6, 7) of mice subjected to 10 weeks of HFD plus L-NAME regimens (One-way ANOVA with Bonferroni's multiple comparisons test). At 10 weeks post-injection of RGD nanoparticles, cardiac systolic function parameters were assessed by echocardiography, including (**L**) FS and (**M**) LVRI (n=7, 2, 9, differences between sham group and HFpEF-siIRF1 group only analyzed using Student's t-test). **N**, Ratio of cardiac ventricle weight to tibia length (TL) (n=7, 7, 10, One-way ANOVA with Bonferroni's multiple comparisons test).

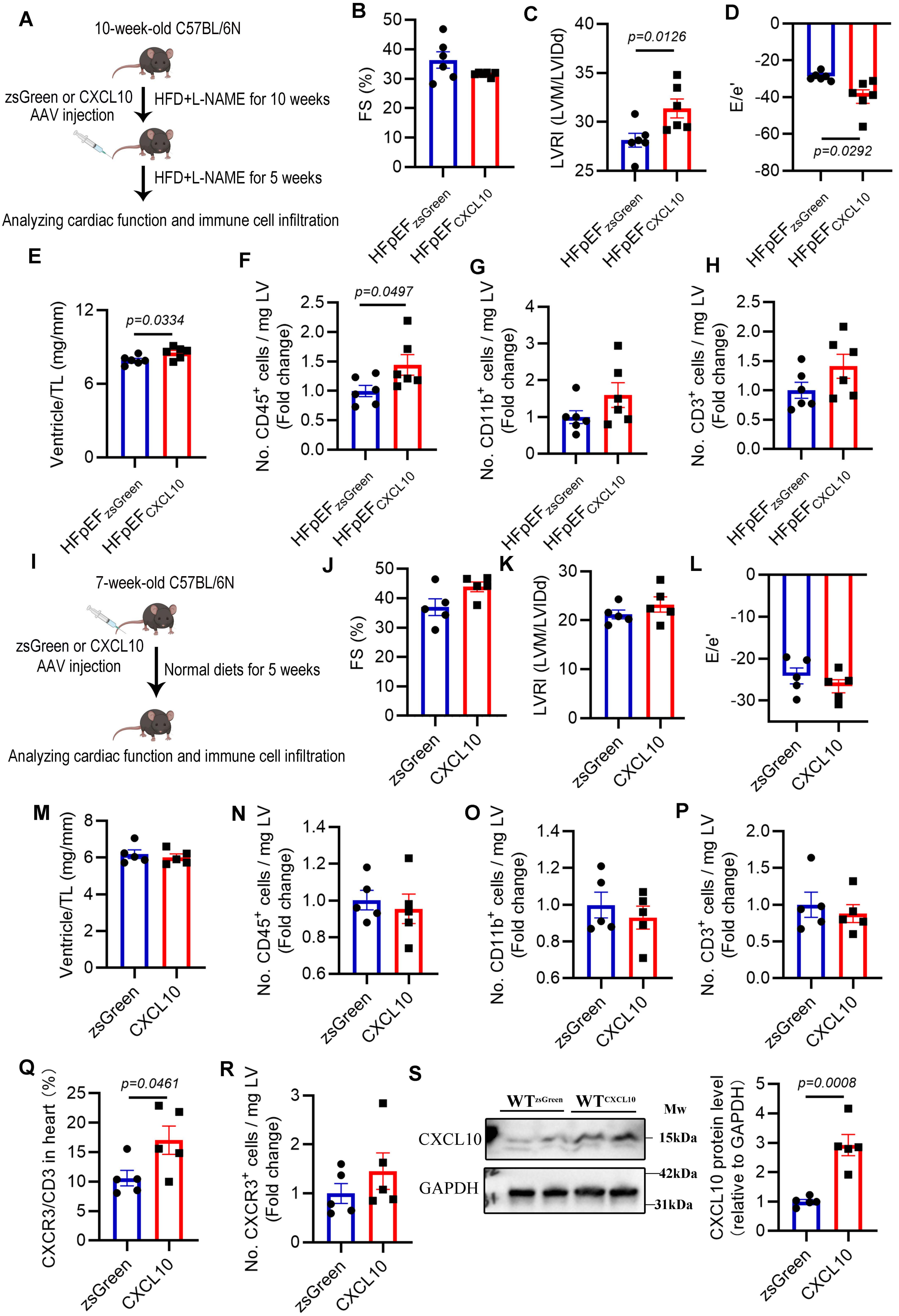
A
10-week-old C57BL/6N
zsGreen or CXCL10
AAV injection
HFD+L-NAME for 10 weeks
HFD+L-NAME for 5 weeks
Analyzing cardiac function and immune cell infiltration
B
FS (%)
HFpEF zsGreen
HFpEF CXCL10
C
LVRI (LVM/LVIDd)
p=0.0126
D
E/e'
p=0.0292
E
Ventricle/TL (mg/mm)
p=0.0334
F
No. CD45+ cells / mg LV
(Fold change)
p=0.0497
G
No. CD11b+ cells / mg LV
(Fold change)
H
No. CD3+ cells / mg LV
(Fold change)
I
7-week-old C57BL/6N
zsGreen or CXCL10
AAV injection
Normal diets for 5 weeks
Analyzing cardiac function and immune cell infiltration
J
FS (%)
zsGreen
CXCL10
K
LVRI (LVM/LVIDd)
L
E/e'
M
Ventricle/TL (mg/mm)
N
No. CD45+ cells / mg LV
(Fold change)
O
No. CD11b+ cells / mg LV
(Fold change)
P
No. CD3+ cells / mg LV
(Fold change)
Q
CXCR3/CD3 in heart (%)
p=0.0461
R
No. CXCR3+ cells / mg LV
(Fold change)
S
WTzsGreen
WTCXCL10
Mw
CXCL10
15kDa
GAPDH
42kDa
31kDa
CXCL10 protein level
(relative to GAPDH)
p=0.0008

Appendix Figure S11 The role of CXCL10 in cardiac function and immune cell infiltration in heart was investigated in both HFpEF disease state (A-H) and healthy state (I-S). **A**, Workflow of the experimental design to investigate the role of CXCL10 during HFpEF pathogenesis. Echocardiogrphic parameters are shown in: (**B**) FS (n=6, Mann-Whitney test); (**C**) LVRI (n=6, Student's t-test); (**D**) E/e′ ratio (n=6, Welch's t-test). **E**, Ratio of cardiac ventricle weight to TL (n=6; Student’s t-test). Flow cytometric quantification of (**F**) CD45+ cells, (**G**) CD11b+ cells, and (**H**) CD3+ T cells in the left ventricle (n=6, Student's t-test). **I**, Workflow of the experimental design to investigate the role of CXCL10 in healthy hearts. Cardiac function was assessed by echocardiography, shown in: (**J**) FS; (**K**) LVRI; (**L**) E/e′ ratio. **M**, Ratio of cardiac ventricle weight to TL. Flow cytometric quantification of (**N**) CD45+ T cells, (**O**) CD11b+ cells, (**P**) CD3+ cells, (**Q**) CXCR3+ cells, and (**R**) CXCR3+/CD3+ ratio in the left ventricle. (**S**) Representative western blot images of cardiac CXCL10 protein expression (left panel) and corresponding quantification (right panel). N=5 (**J-S**), significant differences were analyzed using Student's t-test.

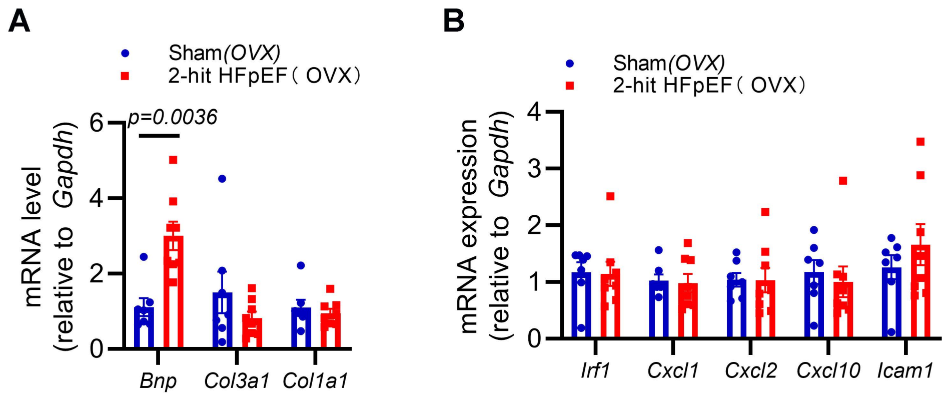
A
Sham(OVX)
2-hit HFpEF（OVX）
p=0.0036
mRNA level
(relative to Gapdh)
0
2
4
6
Bnp
Col3a1
Col1a1
B
Sham(OVX)
2-hit HFpEF（OVX）
mRNA expression
(relative to Gapdh)
0
1
2
3
4
Irf1
Cxcl1
Cxcl2
Cxcl10
Icam1

Appendix Figure S12 A, *Bnp, Col3a1,* and *Col1a1* mRNA abundance in female mouse left ventricles (n=7, 8). Data for *Bnp* were analyzed using the Mann-Whitney test. **B**, *Irf1*, *Cxcl1*, *Cxcl2*, *Cxcl10* and *Icam 1* mRNA abundance (n=7, 8).

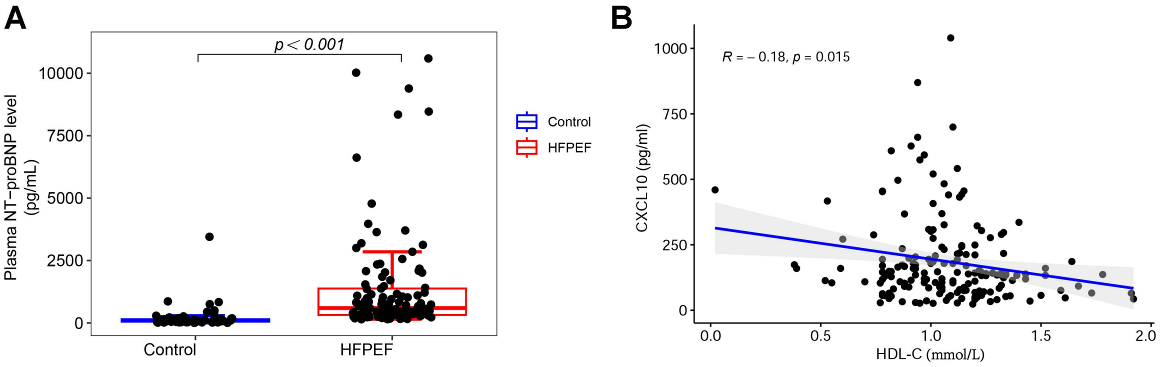
A
p < 0.001
Plasma NT−proBNP level (pg/mL)
10000
7500
5000
2500
0
Control
HFPEF
Control
HFPEF
B
R = − 0.18, p = 0.015
CXCL10 (pg/ml)
1000
750
500
250
0
0.0
0.5
1.0
1.5
2.0
HDL-C (mmol/L)

Appendix Figure S13 A, Plasma NT-proBNP levels were quantitatively assessed using ELISA in both healthy controls and patients with HFpEF (n=67, 109; Mann-Whitney test). **B**, The correlation between plasma CXCL10 levels and HDL-C concentrations was evaluated using Spearman's rank correlation analysis.

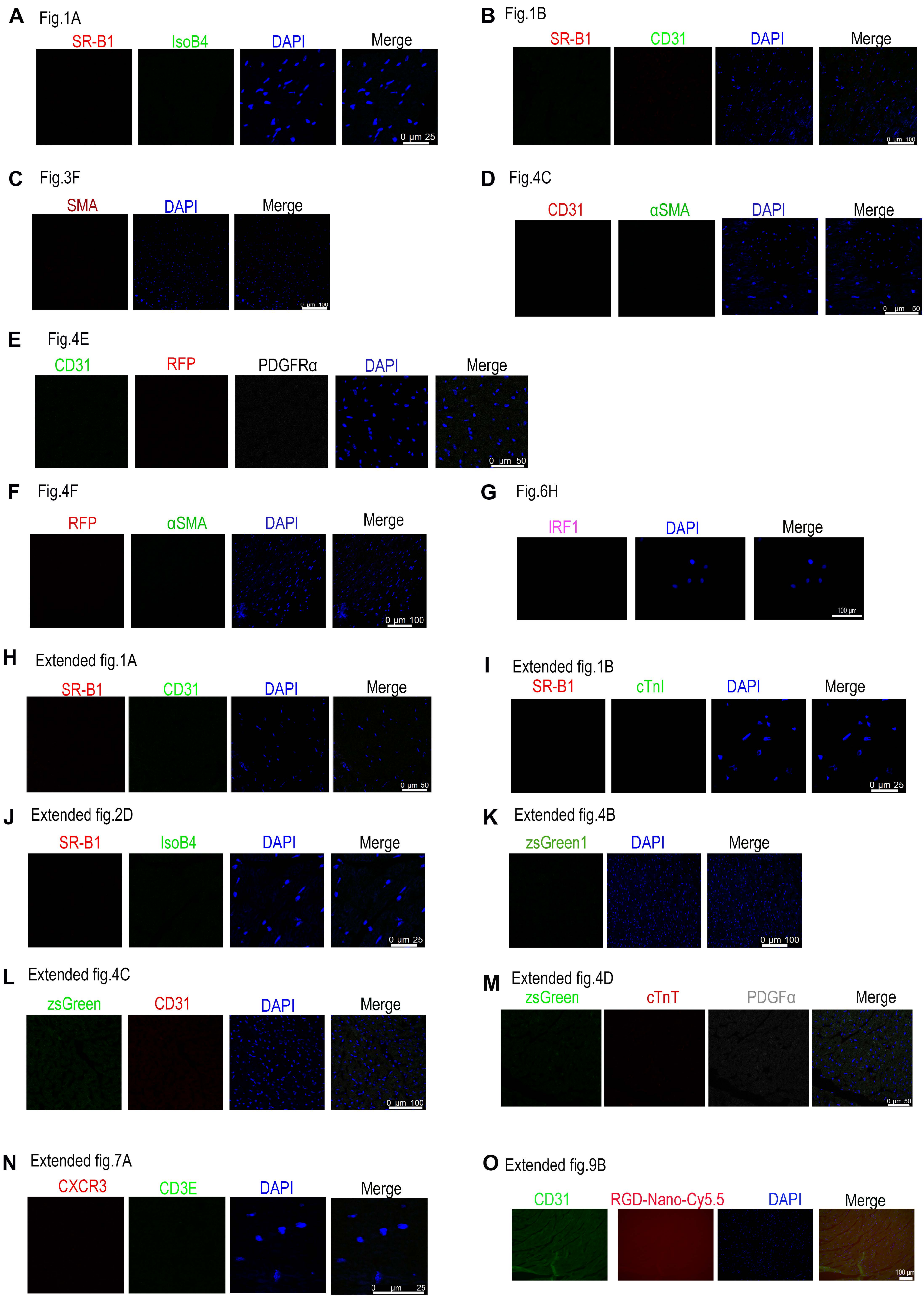
A Fig.1A
SR-B1
IsoB4
DAPI
Merge
0 μm 25
B Fig.1B
SR-B1
CD31
DAPI
Merge
C Fig.3F
SMA
DAPI
Merge
D Fig.4C
CD31
αSMA
DAPI
Merge
0 μm 50
E Fig.4E
CD31
RFP
PDGFRα
DAPI
Merge
0 μm 50
F Fig.4F
RFP
αSMA
DAPI
Merge
0 μm 100
G Fig.6H
IRF1
DAPI
Merge
H Extended fig.1A
SR-B1
CD31
DAPI
Merge
I Extended fig.1B
SR-B1
cTnI
DAPI
Merge
0 μm 25
J Extended fig.2D
SR-B1
IsoB4
DAPI
Merge
0 μm 25
K Extended fig.4B
zsGreen1
DAPI
Merge
0 μm 100
L Extended fig.4C
zsGreen
CD31
DAPI
Merge
0 μm 100
M Extended fig.4D
zsGreen
cTnT
PDGFα
Merge
N Extended fig.7A
CXCR3
CD3E
DAPI
Merge
0 μm 25
O Extended fig.9B
CD31
RGD-Nano-Cy5.5
DAPI
Merge

Appendix Figure S14 All negative controls for immunofluorescence imaging are presented, with each panel clearly labeled to indicate the corresponding fluorescent image number. Tissue sections or cell culture were incubated with antibody diluent in the absence of the primary antibody, while all other experimental conditions remained consistent.

Appendix Table S1 Baseline characteristics of tissue samples from the control group and patients diagnosed with cardiomyopathy-related HFpEF

	Control (n=5)	HFpEF (n=12)
Clinical Characteristics		
Age (year)	-	54.83 ± 2.91
Female gender, n (%)	1 (20.00)	6 (50.00)
SBP (mmHg)	-	132.83 ± 3.00
DBP (mmHg)	-	75.67 ± 3.64
HR (beats/min)	-	72.50 ± 2.91
BMI (kg/m^2)	-	27.92 ± 0.91
Echocardiography		
LVEF (%)	-	68.00 ± 2.02
Septal e’ (cm/s)	-	4.66 ± 0.40
Lateral e’ (cm/s)	-	6.18 ± 0.48
E/e’	-	15.35 (14.23, 22.18)
PASP (mmHg)	-	36.00 (31.00, 37.50)
LVPWT (mm)	-	12.00 (11.00, 14.50)
LVIDD (mm)	-	4.51 ± 0.17
RWT	-	0.56 (0.48, 0.65)
LVMI (g/m^2)	-	166.92 ± 13.21
Medical history		
Smoking, n (%)	-	3 (25.00)
Drinking, n (%)	-	4 (33.33)
Hypertension, n (%)	-	6 (50.00)
DM, n (%)	-	4 (33.33)
AF, n (%)	-	2 (16.67)
CAD, n (%)	-	3 (25.00)
Medical history		
Diuretics, n (%)	-	3 (25.00)
Beta blocker, n (%)	-	4 (33.33)
CCB, n (%)	-	3 (25.00)
ACEI/ARB, n (%)	-	1 (8.33)
Laboratory Examination		
Cr (μmol/L)	-	75.18 ± 4.04
NT-proBNP (pg/ml)	-	2563.74 ± 535.14
HDL-C (mmol/L)	-	0.98 ± 0.09

Values are median (IQR), mean ± SEM or frequencies (%). SBP, systolic blood pressure; DBP, diastolic blood pressure; HR, heart rate; BMI, body mass index; LVEF, left ventricular ejection fraction; PASP, pulmonary artery systolic pressure; LVPWT, left ventricular diastolic posterior wall thickness; LVIDD, left ventricular internal diameter, diastolic; RWT, relative wall thickness; LVMI, left ventricular mass index; DM, diabetes mellitus; AF, atrial fibrillation; CAD, cardiac artery disease; CCB, calcium channel blockers;

ACEI, angiotensin-converting enzyme inhibitor; ARB, angiotensin receptor blocker; Cr, creatinine; NT-proBNP, N-terminal pro-B-type natriuretic peptide; HDL-C, high-density lipoprotein cholesterol.

Appendix Table S2 Baseline characteristics of control group and patients diagnosed with HFpEF

	Control (n=67)	HFpEF (n=109)	P
Clinical Characteristics			
Age (year, IQR)	66 (63, 69.5)	67 (63, 71)	0.37
Female gender, n (%)	35 (52.24)	52 (47.71)	0.67
SBP (mmHg, IQR)	134 (129, 150)	137 (128, 148)	0.63
DBP (mmHg, IQR)	80 (72, 86)	76 (70, 84)	0.08
HR (beats/min, IQR)	75 (65, 79)	71 (66, 80)	0.38
BMI (kg/m^2, IQR)	26.17 (24.16, 27.68)	25.26 (23.44, 27.34)	0.20
Echocardiography			
LVEF (%, IQR)	65.00 (61.50, 67.00)	63.00 (58.00, 65.00)	0.01
Septal e' (cm/s, IQR)	12.60 (11.05, 13.55)	5.20 (5.00, 6.40)	<0.001
Lateral e' (cm/s, IQR)	17.30 (15.95, 19.05)	7.00 (6.00, 8.00)	<0.001
E/e' (IQR)	4.83 (4.43, 6.11)	10.91 (8.83, 13.67)	<0.001
PASP (mmHg, IQR)	9.20 (8.05, 10.65)	28.00 (25.00, 30.00)	<0.001
LVPWT (mm, IQR)	10.00 (9.00, 10.50)	10.00 (10.00, 11.00)	<0.001
LVIDD (mm, IQR)	45.00 (42.00, 47.50)	45.00 (41.00, 48.00)	0.99
RWT (IQR)	0.44 (0.40, 0.47)	0.47 (0.43, 0.51)	0.002
LVMI (g/m^2, IQR)	87.26 (75.19, 99.71)	99.78 (87.28, 115.97)	<0.001
Medical history			
Smoking, n (%)	19 (28.36)	39 (35.78)	0.39
Drinking, n (%)	18 (26.87)	36 (33.03)	0.49
Hypertension, n (%)	21 (31.34)	78 (71.56)	<0.001
DM, n (%)	13 (19.40)	43 (39.45)	0.01
AF, n (%)	7 (10.45)	21 (19.27)	0.18
CAD, n (%)	12 (17.91)	75 (68.81)	<0.001
Medicine history			
Diuretics, n (%)	2 (2.99)	22 (20.18)	0.003
Beta blocker, n (%)	3 (4.48)	44 (40.37)	<0.001
CCB, n (%)	11 (16.42)	27 (24.77)	0.26
ACEI/ARB, n (%)	9 (13.43)	20 (18.35)	0.52
Laboratory Examination			
Cr, (μmol/L, IQR)	63.00 (56.00, 72.00)	67.00 (58.00, 80.00)	0.16
NT-proBNP, (pg/ml, IQR)	99.95 (51.26, 150.99)	597.20 (320.90, 1380.00)	<0.001
HDL-C (mmol/L, IQR)	1.20 (1.05, 1.32)	1.01 (0.88, 1.13)	<0.001
CXCL10 (pg/ml, IQR)	114.75 (68.92, 145.20)	159.95 (91.46, 296.91)	0.001

Values are median (IQR) or frequencies (%). SBP, systolic blood pressure; DBP, diastolic blood pressure; HR, heart rate; BMI, body mass index; LVEF, left ventricular ejection fraction; PASP, pulmonary artery systolic pressure; LVPWT, left ventricular diastolic posterior wall thickness; LVIDD, left ventricular internal diameter, diastolic; RWT, relative wall thickness; LVMI, left ventricular mass index; DM, diabetes mellitus; AF, atrial fibrillation; CAD, cardiac artery disease; CCB, calcium channel blockers; ACEI, angiotensin-converting enzyme inhibitor; ARB, angiotensin receptor blocker; Cr, creatinine; NT-proBNP, N-terminal pro-B-type natriuretic peptide; HDL-C, high-density lipoprotein cholesterol.

Appendix Table S3 Univariate and multivariate logistic regression analysis of major cardiovascular risk factors on HFpEF pathogenesis

Variable	Univariate analysis			Multivariate analysis		
	P value	OR	95%CI	*P* value	OR	95%CI
Age	0.90	1.00	(0.96, 1,04)			
Gender	0.56	1.20	(0.65, 2.21)			
BMI	0.54	0.97	(0.89, 1.06)			
DBP	0.24	0.98	(0.96, 1.01)			
Smoking	0.31	1.41	(0.73, 1.72)			
AF	0.17	2.05	(0.82, 5.11)			
CAD	<0.001	10.11	(4.80, 21.29)	<0.001	8.05	(3.54, 18.34)
HBP	<0.001	5.51	(2.84, 10.70)	0.003	3.25	(1.51, 6.98)
DM	0.007	2.71	(1.32, 5.54)	0.006	3.55	(1.45, 8.70)
Log CXCL10	0.003	4.28	(1.64, 11.15)	0.048	3.28	(1.01, 10.65)

OR, odds ratio; CI, confidence interval.

Appendix Table S4 Exact P Values for the partial figures reporting P-Value ranges.

Figure Name		Group Name	Exact P Value
Figure 1	Fig.1E	NC vs. HFpEF	0.000032
Figure 2	Fig.2B	*$S^{fl/fl}$ vs.$S^{\Delta EC}$*	0.000003
	Fig 2C	*$S^{fl/fl}$ vs.$S^{\Delta EC}$*	0.000031
	Fig.2G LVPWd	NC:$S^{fl/fl}$ vs. NC:$S^{\Delta EC}$	>0.999999999999
		NC:$S^{fl/fl}$ vs. HFpEF:$S^{fl/fl}$	7.30001E-05
		NC:$S^{fl/fl}$ vs. HFpEF:$S^{\Delta EC}$	3.96E-10
		NC:$S^{\Delta EC}$ vs. HFpEF:$S^{fl/fl}$	0.001252442
		NC:$S^{\Delta EC}$ vs. HFpEF:$S^{\Delta EC}$	5.384E-09
		HFpEF:$S^{fl/fl}$ vs. HFpEF:$S^{\Delta EC}$	0.000737293
	Fig.2I LVIR	NC:$S^{fl/fl}$ vs. NC:$S^{\Delta EC}$	>0.99999999999999
		NC:$S^{fl/fl}$ vs. HFpEF:$S^{fl/fl}$	4.63481E-05
		NC:$S^{fl/fl}$ vs. HFpEF:$S^{\Delta EC}$	1E-12
		NC:$S^{\Delta EC}$ vs. HFpEF:$S^{fl/fl}$	0.00215645
		NC:$S^{\Delta EC}$ vs. HFpEF:$S^{\Delta EC}$	2.338E-11
		HFpEF:$S^{fl/fl}$ vs. HFpEF:$S^{\Delta EC}$	7.56744E-07
	Fig.2K MPI	NC:$S^{fl/fl}$ vs. NC:$S^{\Delta EC}$	>0.999999999999999
		NC:$S^{fl/fl}$ vs. HFpEF:$S^{fl/fl}$	2.49347E-06
		NC:$S^{fl/fl}$ vs. HFpEF:$S^{\Delta EC}$	1.0218E-11
		NC:$S^{\Delta EC}$ vs. HFpEF:$S^{fl/fl}$	2.76528E-05
		NC:$S^{\Delta EC}$ vs. HFpEF:$S^{\Delta EC}$	7.7204E-11
		HFpEF:$S^{fl/fl}$ vs. HFpEF:$S^{\Delta EC}$	0.000295228
	Fig.2L E/e'	NC:$S^{fl/fl}$ vs. NC:$S^{\Delta EC}$	>0.999999999999999
		NC:$S^{fl/fl}$ vs. HFpEF:$S^{fl/fl}$	0.000500158
		NC:$S^{fl/fl}$ vs. HFpEF:$S^{\Delta EC}$	2.1509E-11
		NC:$S^{\Delta EC}$ vs. HFpEF:$S^{fl/fl}$	0.001711282
		NC:$S^{\Delta EC}$ vs. HFpEF:$S^{\Delta EC}$	6.2635E-11
		HFpEF:$S^{fl/fl}$ vs. HFpEF:$S^{\Delta EC}$	3.17154E-06
	Fig.2N Running Distance	NC:$S^{fl/fl}$ vs. NC:$S^{\Delta EC}$	>0.999999999999999
		NC:$S^{fl/fl}$ vs. HFpEF:$S^{fl/fl}$	1.72109E-05
		NC:$S^{fl/fl}$ vs. HFpEF:$S^{\Delta EC}$	3.40339E-08
		NC:$S^{\Delta EC}$ vs. HFpEF:$S^{fl/fl}$	5.35315E-05
		NC:$S^{\Delta EC}$ vs. HFpEF:$S^{\Delta EC}$	8.15949E-08
		HFpEF:$S^{fl/fl}$ vs. HFpEF:$S^{\Delta EC}$	0.008021452
Figure 3	Fig.3A Ventricle/TL	NC:$S^{fl/fl}$ vs. NC:$S^{\Delta EC}$	>0.999999999999999
		NC:$S^{fl/fl}$ vs. HFpEF:$S^{fl/fl}$	2.3153E-05
		NC:$S^{fl/fl}$ vs. HFpEF:$S^{\Delta EC}$	1.81978E-09

		NC:$S^{\Delta EC}$ vs. HFpEF:$S^{fl/fl}$	9.60633E-05
		NC:$S^{\Delta EC}$ vs. HFpEF:$S^{\Delta EC}$	6.7309E-09
		HFpEF:$S^{fl/fl}$ vs. HFpEF:$S^{\Delta EC}$	0.011708303
	Fig.3B LW / TL	NC:$S^{fl/fl}$ vs. NC:$S^{\Delta EC}$	>0.999999999999999
		NC:$S^{fl/fl}$ vs. HFpEF:$S^{fl/fl}$	0.003465676
		NC:$S^{fl/fl}$ vs. HFpEF:$S^{\Delta EC}$	9.62333E-07
		NC:$S^{\Delta EC}$ vs. HFpEF:$S^{fl/fl}$	0.003498021
		NC:$S^{\Delta EC}$ vs. HFpEF:$S^{\Delta EC}$	9.71738E-07
		HFpEF:$S^{fl/fl}$ vs. HFpEF:$S^{\Delta EC}$	0.044875531
	Fig.3D Relative fibrosis area	NC:$S^{fl/fl}$ vs. NC:$S^{\Delta EC}$	>0.999999999999999
		NC:$S^{fl/fl}$ vs. HFpEF:$S^{fl/fl}$	0.002083933
		NC:$S^{fl/fl}$ vs. HFpEF:$S^{\Delta EC}$	1.52197E-08
		NC:$S^{\Delta EC}$ vs. HFpEF:$S^{fl/fl}$	0.001762658
		NC:$S^{\Delta EC}$ vs. HFpEF:$S^{\Delta EC}$	1.29149E-08
		HFpEF:$S^{fl/fl}$ vs. HFpEF:$S^{\Delta EC}$	0.001320255
	Fig.3E: Cola3a1	NC:flox vs. NC:ec	>0.999999999999999
		NC:flox vs. HFpEF:flox	0.00410197
		NC:flox vs. HFpEF:ec	8.80127E-10
		NC:ec vs. HFpEF:flox	0.005121372
		NC:ec vs. HFpEF:ec	1.06975E-09
		HFpEF:flox vs. HFpEF:ec	1.66713E-05
	Fig.3E: Cola1a1	NC:flox vs. NC:ec	>0.999999999999999
		NC:flox vs. HFpEF:flox	0.292053538
		NC:flox vs. HFpEF:ec	0.001121814
		NC:ec vs. HFpEF:flox	0.323390354
		NC:ec vs. HFpEF:ec	0.001280096
		HFpEF:flox vs. HFpEF:ec	0.190759275
	Fig.3E: BNP	NC:flox vs. NC:ec	>0.999999999999999
		NC:flox vs. HFpEF:flox	0.000429403
		NC:flox vs. HFpEF:ec	7.10267E-10
		NC:ec vs. HFpEF:flox	0.000280922
		NC:ec vs. HFpEF:ec	4.98652E-10
		HFpEF:flox vs. HFpEF:ec	0.000122107
	Fig.3F	NC:$S^{fl/fl}$ vs. NC:$S^{\Delta EC}$	>0.999999999999999
		NC:$S^{fl/fl}$ vs. HFpEF:$S^{fl/fl}$	0.000300311
		NC:$S^{fl/fl}$ vs. HFpEF:$S^{\Delta EC}$	3.86224E-07
		NC:$S^{\Delta EC}$ vs. HFpEF:$S^{fl/fl}$	0.00053118
		NC:$S^{\Delta EC}$ vs. HFpEF:$S^{\Delta EC}$	5.72422E-07
		HFpEF:$S^{fl/fl}$ vs. HFpEF:$S^{\Delta EC}$	0.000410611
Figure 4	Fig.4B:Cd31	NC vs. TGF-beta	>0.9999999999
		NC vs. TGF-beta+H2O2	0.074957278
		NC vs. TGF-beta+IL-1b	0.011005872

		TGF-beta vs. TGF-beta+H2O2	0.117829459
		TGF-beta vs. TGF-beta+IL-1b	0.017998633
		TGF-beta+H2O2 vs. TGF-beta+IL-1b	>0.9999999999
	Fig.4B:Cdh5	NC vs. TGF-beta	0.000296924
		NC vs. TGF-beta+H2O2	0.000107935
		NC vs. TGF-beta+IL-1b	0.132730825
		TGF-beta vs. TGF-beta+H2O2	>0.9999999999
		TGF-beta vs. TGF-beta+IL-1b	0.122034718
		TGF-beta+H2O2 vs. TGF-beta+IL-1b	0.04885462
	Fig.4B:Col3a1	NC vs. TGF-beta	0.003048923
		NC vs. TGF-beta+H2O2	0.008714946
		NC vs. TGF-beta+IL-1b	>0.9999999999
		TGF-beta vs. TGF-beta+H2O2	>0.9999999999
		TGF-beta vs. TGF-beta+IL-1b	0.008714946
		TGF-beta+H2O2 vs. TGF-beta+IL-1b	0.02299695
	Fig.4B:Col1a1	NC vs. TGF-beta	0.003048923
		NC vs. TGF-beta+H2O2	0.000757041
		NC vs. TGF-beta+IL-1b	0.149844172
		TGF-beta vs. TGF-beta+H2O2	>0.9999999999
		TGF-beta vs. TGF-beta+IL-1b	>0.9999999999
		TGF-beta+H2O2 vs. TGF-beta+IL-1b	0.668300285
	Fig.4B:Acta2	NC vs. TGF-beta	0.000148522
		NC vs. TGF-beta+H2O2	0.000949177
		NC vs. TGF-beta+IL-1b	1.99654E-05
		TGF-beta vs. TGF-beta+H2O2	0.999996118
		TGF-beta vs. TGF-beta+IL-1b	0.001333147
		TGF-beta+H2O2 vs. TGF-beta+IL-1b	0.011577489
	Fig.4B:eNOS	NC vs. TGF-beta	>0.9999999999
		NC vs. TGF-beta+H2O2	0.000644613
		NC vs. TGF-beta+IL-1b	9.66E-08
		TGF-beta vs. TGF-beta+H2O2	2.72335E-05
		TGF-beta vs. TGF-beta+IL-1b	0.000000007
		TGF-beta+H2O2 vs. TGF-beta+IL-1b	0.007047323
	Fig.4B:Scarb1	NC vs. TGF-beta	0.000105989
		NC vs. TGF-beta+H2O2	3.76013E-05
		NC vs. TGF-beta+IL-1b	2.21123E-05

		TGF-beta vs. TGF-beta+H2O2	>0.9999999999
		TGF-beta vs. TGF-beta+IL-1b	>0.9999999999
		TGF-beta+H2O2 vs. TGF-beta+IL-1b	>0.9999999999
Figure 5	Fig.5F:Scarb1	HFpEF $S^{fl/fl}$ vs. HFpEF $S^{\Delta EC}$	7.23906E-05
	Fig.5F:Cxcl10	HFpEF $S^{fl/fl}$ vs. HFpEF $S^{\Delta EC}$	1.22187E-05
	Fig.5G:CXCL10	HFpEF $S^{fl/fl}$ vs. HFpEF $S^{\Delta EC}$	6.39807E-05
Figure 6	Fig.6A:SR-B1	siNC vs. siSR-B1	6E-10
	Fig.6A:CXCL2	siNC vs. siSR-B1	6.00623E-05
	Fig.6A:CXCL10	siNC vs. siSR-B1	7.32764E-05
	Fig.6B	Vehicle:siNC vs. Vehicle:siSRB1	>0.999999999999999
		Vehicle:siNC vs. LPS:siNC	0.602836543
		Vehicle:siNC vs. LPS:siSRB1	3.3959E-11
		Vehicle:siSRB1 vs. LPS:siNC	>0.999999999999999
		Vehicle:siSRB1 vs. LPS:siSRB1	4.5498E-11
		LPS:siNC vs. LPS:siSRB1	1.62464E-10
	Fig.6C	Vehicle:siNC vs. Vehicle:siSRB1	7.004E-07
		Vehicle:siNC vs. LPS:siNC	0.000364783
		Vehicle:siNC vs. LPS:siSRB1	1.87E-08
		Vehicle:siSRB1 vs. LPS:siNC	0.849553528
		Vehicle:siSRB1 vs. LPS:siSRB1	0.000364783
		LPS:siNC vs. LPS:siSRB1	7.004E-07
	Fig.6G	Vector vs. IRF1	2.917E-12
	Fig.6I:SR-B1	NC vs. siSR-B1	4.7E-09
		NC vs. siSR-B1/siIRF1	2.892E-07
		siSR-B1 vs. siSR-B1/siIRF1	0.786294612
	Fig.6I:IRF1	NC vs. siSR-B1	0.001042125
		NC vs. siSR-B1/siIRF1	2.048E-07
		siSR-B1 vs. siSR-B1/siIRF1	2.4807E-06
	Fig.6I:CXCL10	NC vs. siSR-B1	0.001877148
		NC vs. siSR-B1/siIRF1	0.990987669
		siSR-B1 vs. siSR-B1/siIRF1	0.002998433
Figure 8	Fig.8A:BNP	Healthy vs. HFpEF	0.000323206
	Fig.8A:Col3a1	Healthy vs. HFpEF	0.000323206
	Fig.8B	Healthy vs. HFpEF	8.70329E-05
	Fig.8E	Linear correlation between Log CXCL10 and Log NT-ProBNP	0.000009685
	Fig.8F:CAD	The effect of CAD variable on HFpEF	6.7728E-07
