# Supplementary figures and images for: Microvascular endothelial scavenger receptor class B type I protects against heart failure with preserved ejection fraction by inhibiting T-cell cardiotropism
Source: EMBO Mol Med. 2026 Apr 13;18(5):1679–706. doi: 10.1038/s44321-026-00405-9 (PMC13179366; doi:10.1038/s44321-026-00405-9)

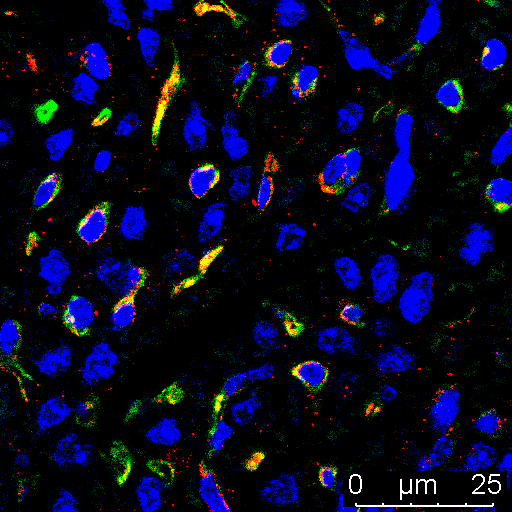

Supplement: Supplementary file 3 — Source data Fig. 1 [file 44321_2026_405_MOESM3_ESM.zip › Figure 1/A/Microvasculature-Merge.tif]

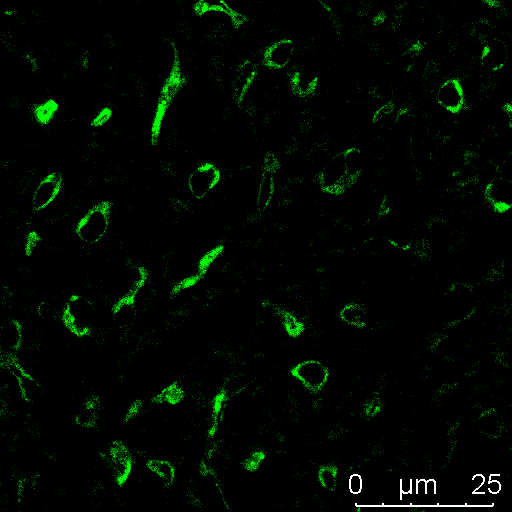

Supplement: Supplementary file 3 — Source data Fig. 1 [file 44321_2026_405_MOESM3_ESM.zip › Figure 1/A/Microvasculature-IB4.tif]

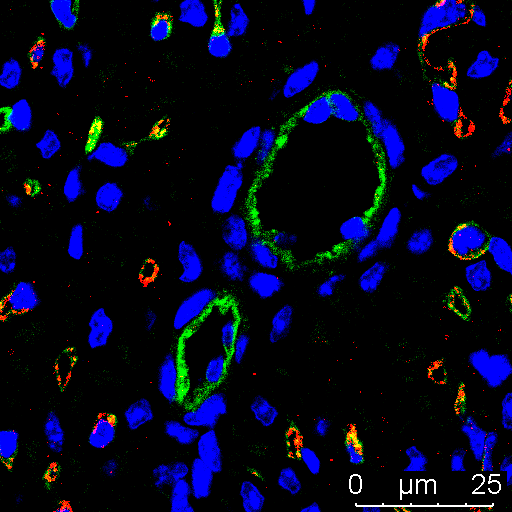

Supplement: Supplementary file 3 — Source data Fig. 1 [file 44321_2026_405_MOESM3_ESM.zip › Figure 1/A/Large vessel-Merge.tif]

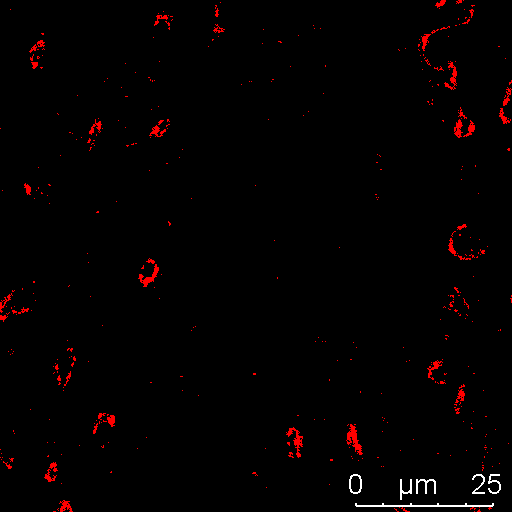

Supplement: Supplementary file 3 — Source data Fig. 1 [file 44321_2026_405_MOESM3_ESM.zip › Figure 1/A/Large vessel-SR-B1.tif]

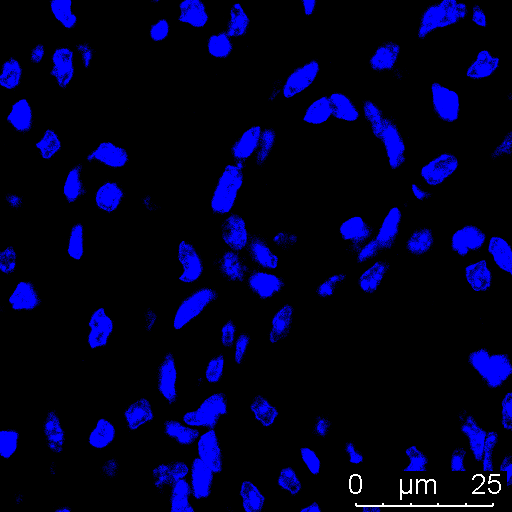

Supplement: Supplementary file 3 — Source data Fig. 1 [file 44321_2026_405_MOESM3_ESM.zip › Figure 1/A/Large vessel-DAPI.tif]

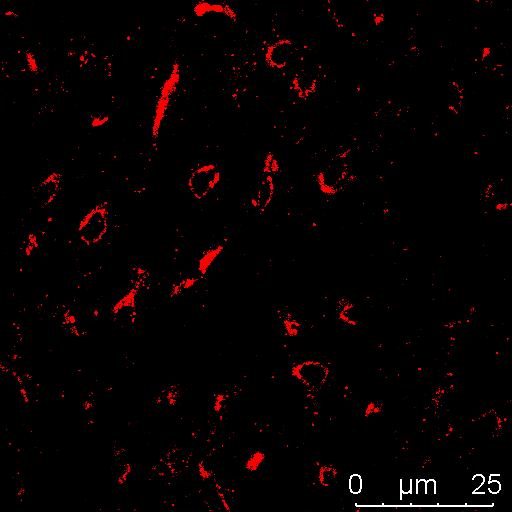

Supplement: Supplementary file 3 — Source data Fig. 1 [file 44321_2026_405_MOESM3_ESM.zip › Figure 1/A/Microvasculature-SR-B1.tif]

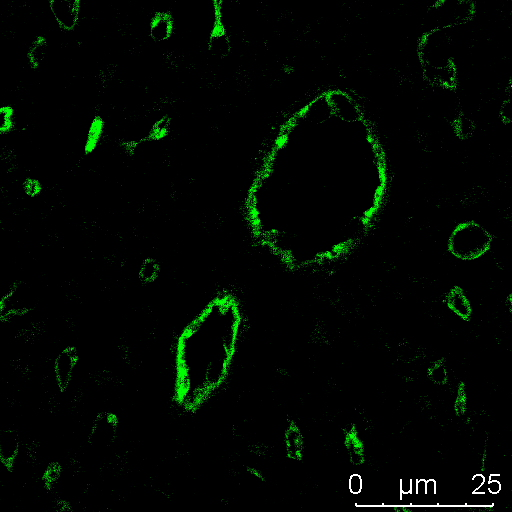

Supplement: Supplementary file 3 — Source data Fig. 1 [file 44321_2026_405_MOESM3_ESM.zip › Figure 1/A/Large vessel-IB4.tif]

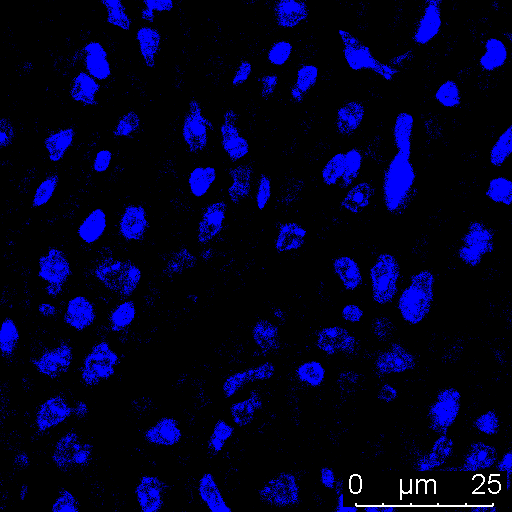

Supplement: Supplementary file 3 — Source data Fig. 1 [file 44321_2026_405_MOESM3_ESM.zip › Figure 1/A/Microvasculature-DAPI.tif]

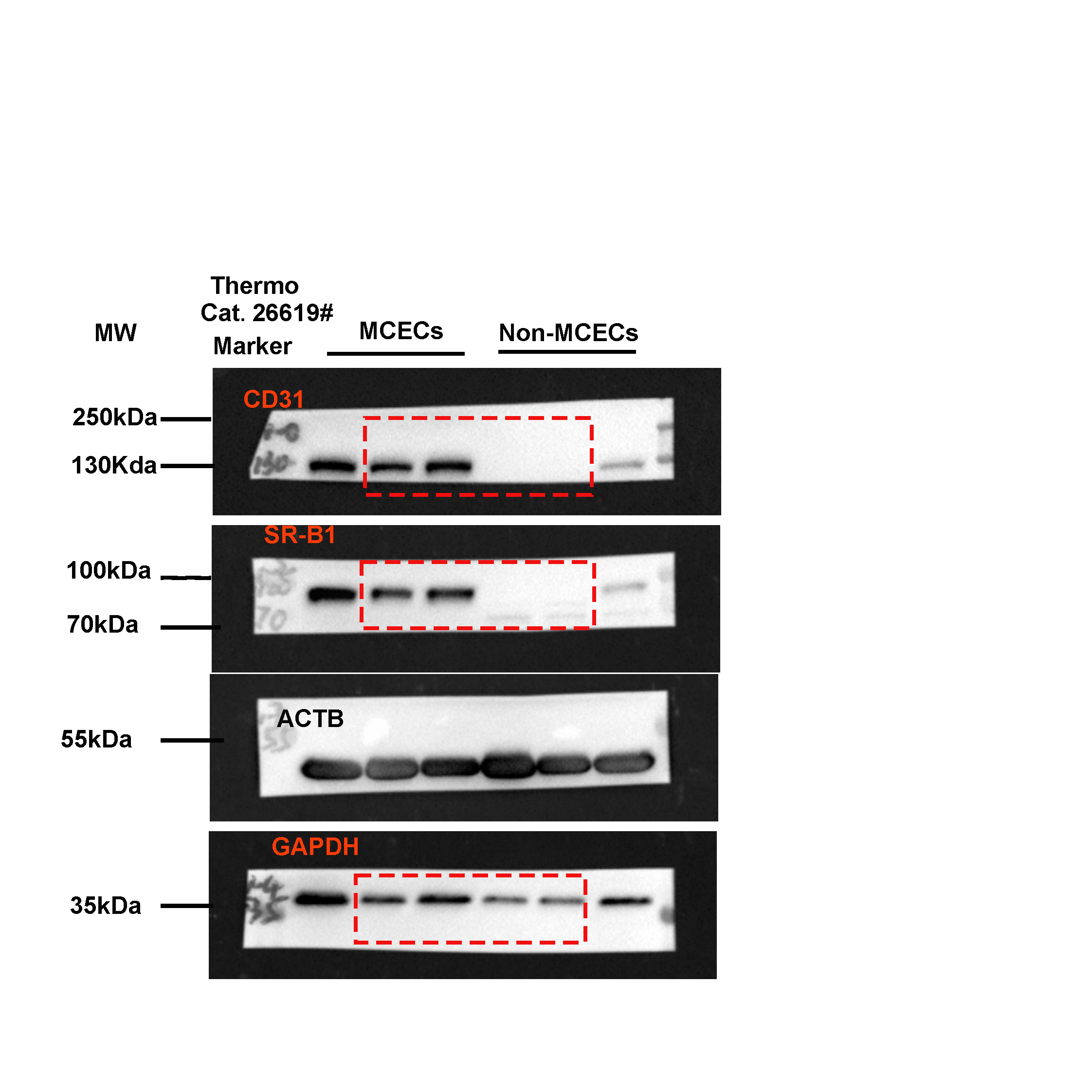

Supplement: Supplementary file 3 — Source data Fig. 1 [file 44321_2026_405_MOESM3_ESM.zip › Figure 1/D/1D.jpg]

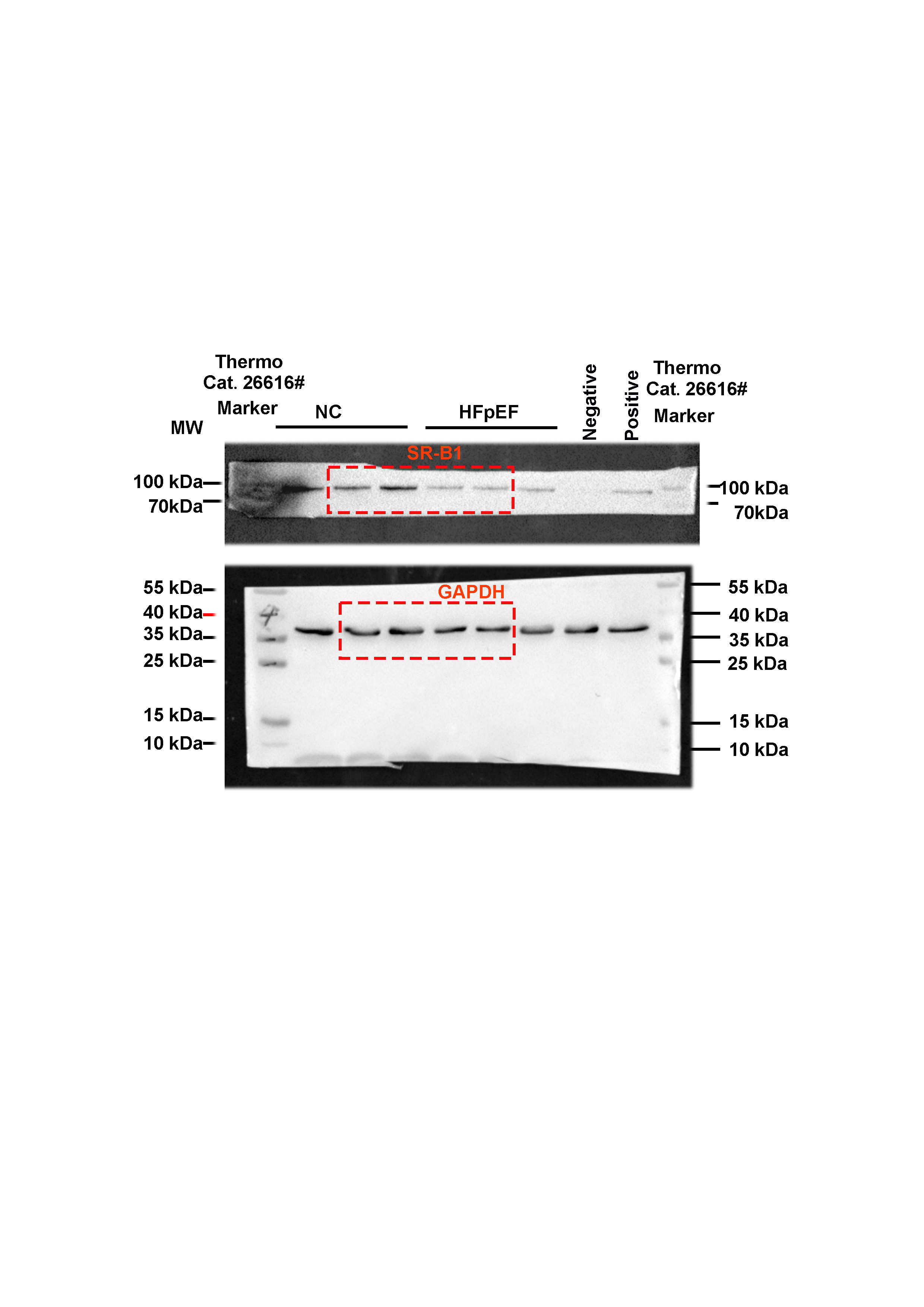

Supplement: Supplementary file 3 — Source data Fig. 1 [file 44321_2026_405_MOESM3_ESM.zip › Figure 1/E/1E.jpg]

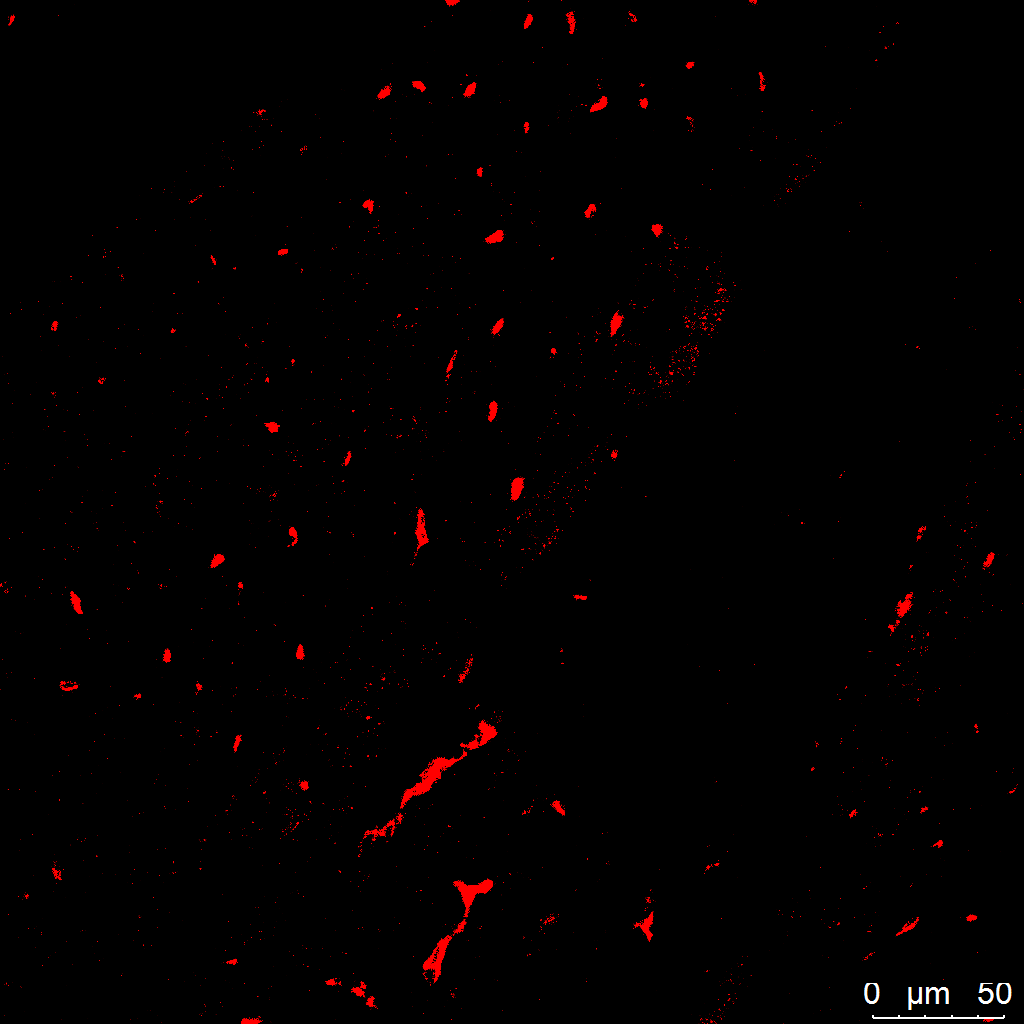

Supplement: Supplementary file 3 — Source data Fig. 1 [file 44321_2026_405_MOESM3_ESM.zip › Figure 1/B/SR-B1.tif]

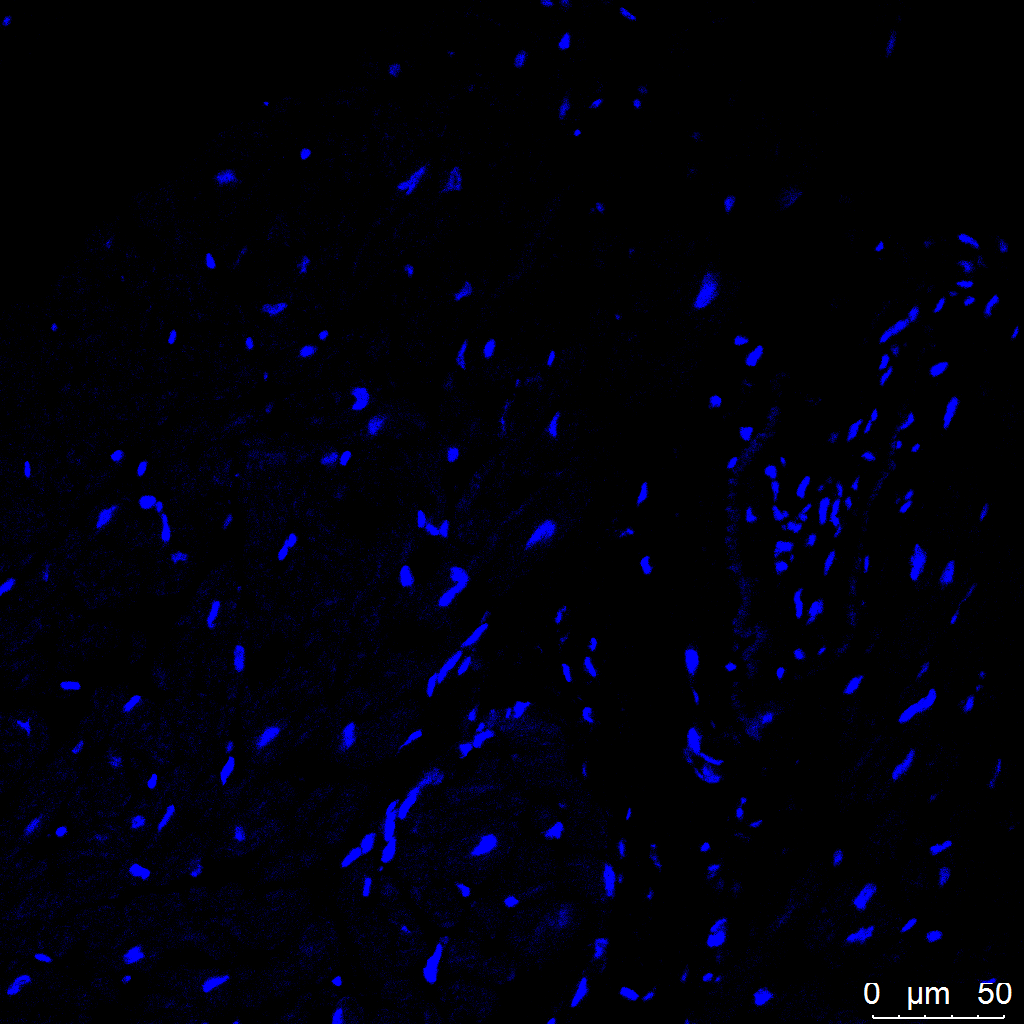

Supplement: Supplementary file 3 — Source data Fig. 1 [file 44321_2026_405_MOESM3_ESM.zip › Figure 1/B/DAPI.tif]

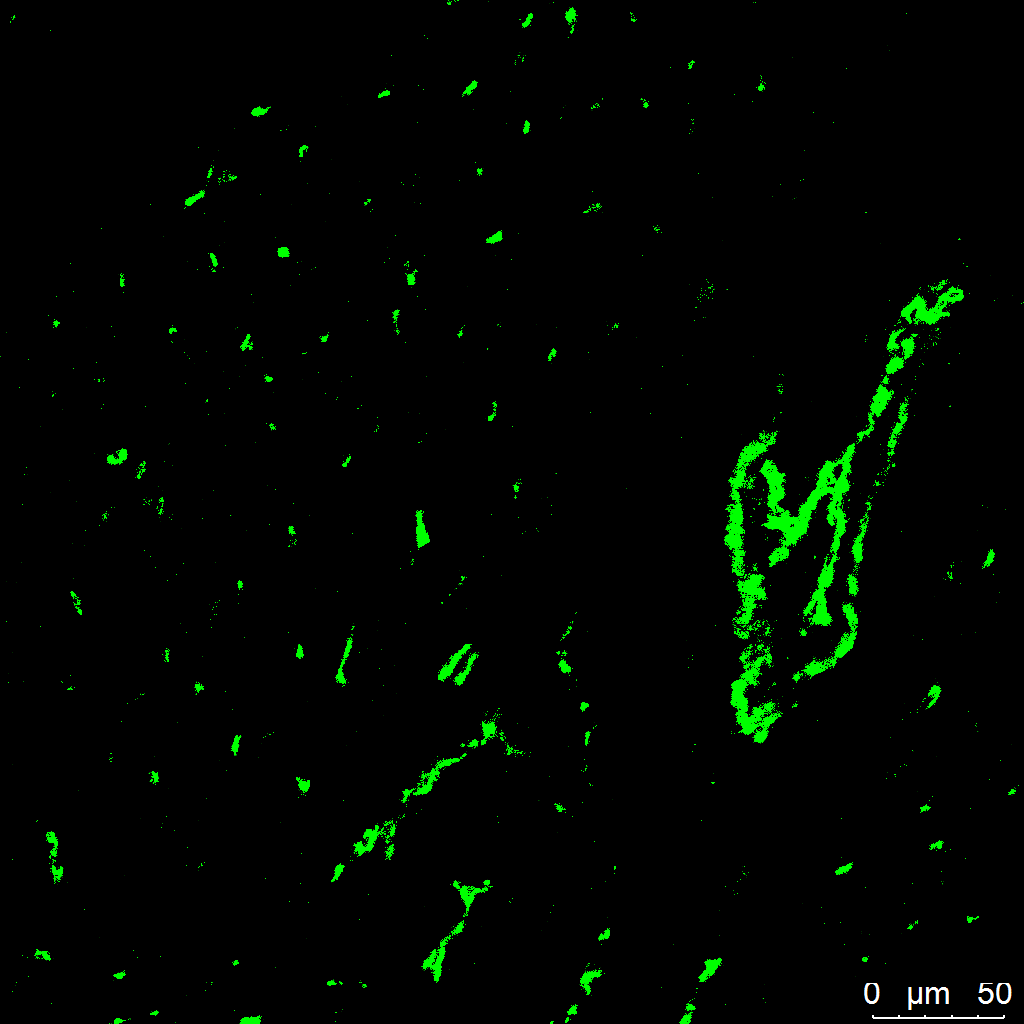

Supplement: Supplementary file 3 — Source data Fig. 1 [file 44321_2026_405_MOESM3_ESM.zip › Figure 1/B/CD31.tif]

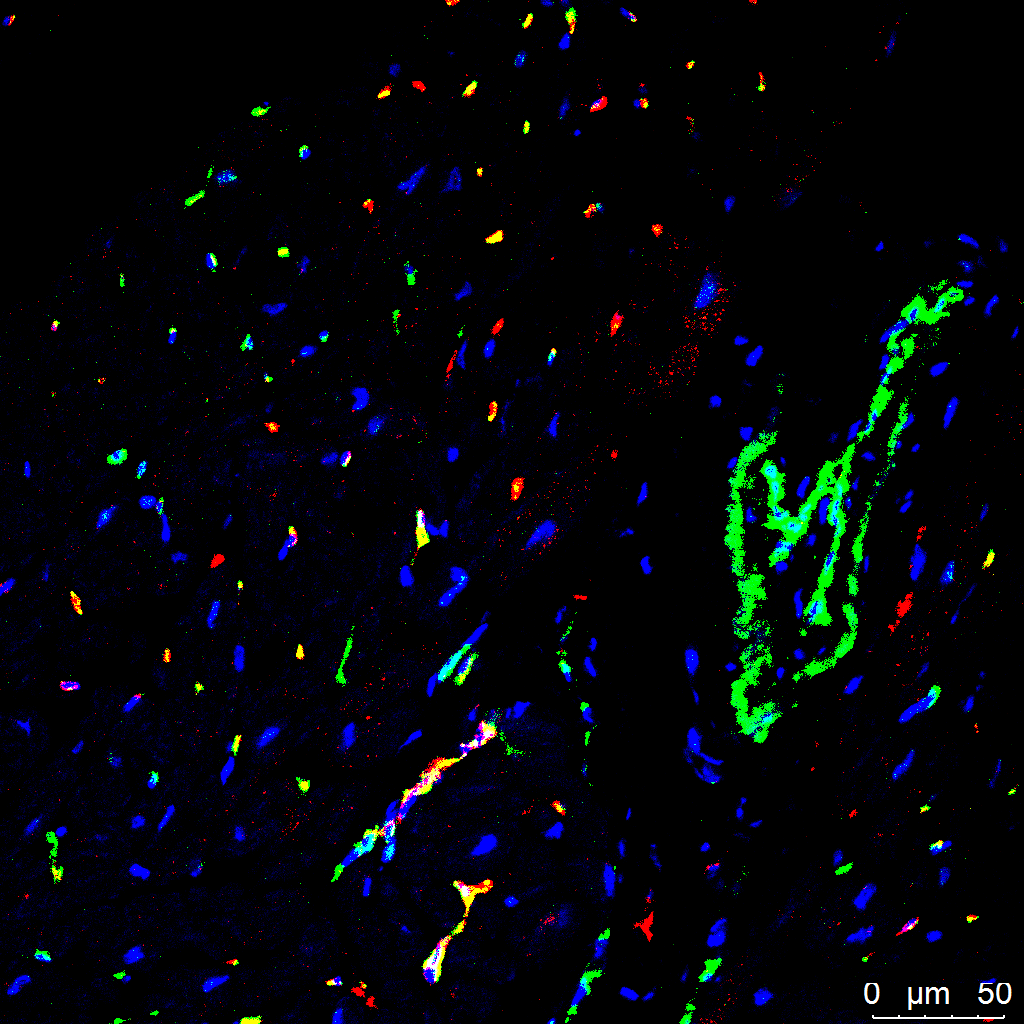

Supplement: Supplementary file 3 — Source data Fig. 1 [file 44321_2026_405_MOESM3_ESM.zip › Figure 1/B/Merge.tif]

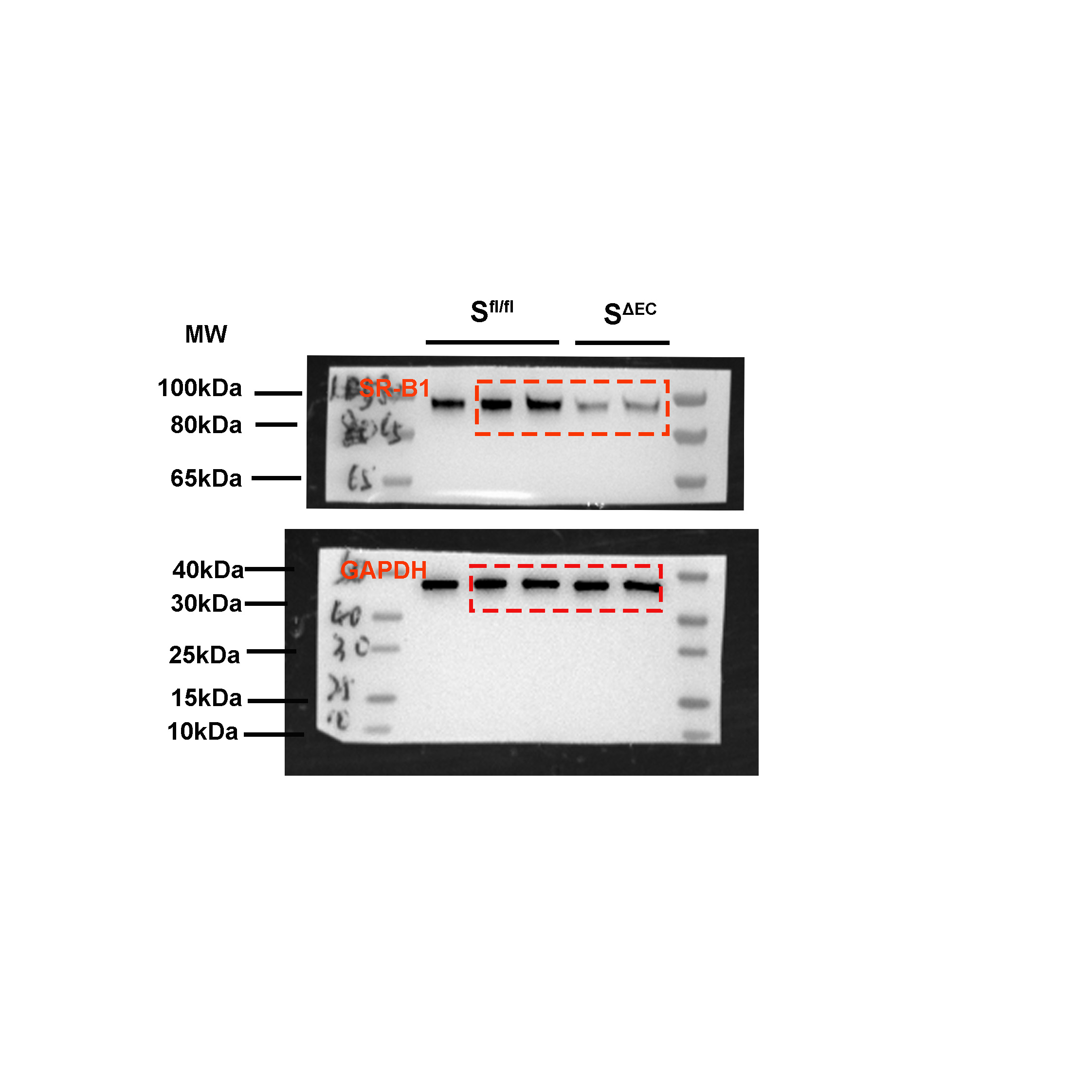

Supplement: Supplementary file 4 — Source data Fig. 2 [file 44321_2026_405_MOESM4_ESM.zip › Figure 2/C/2C.jpg]

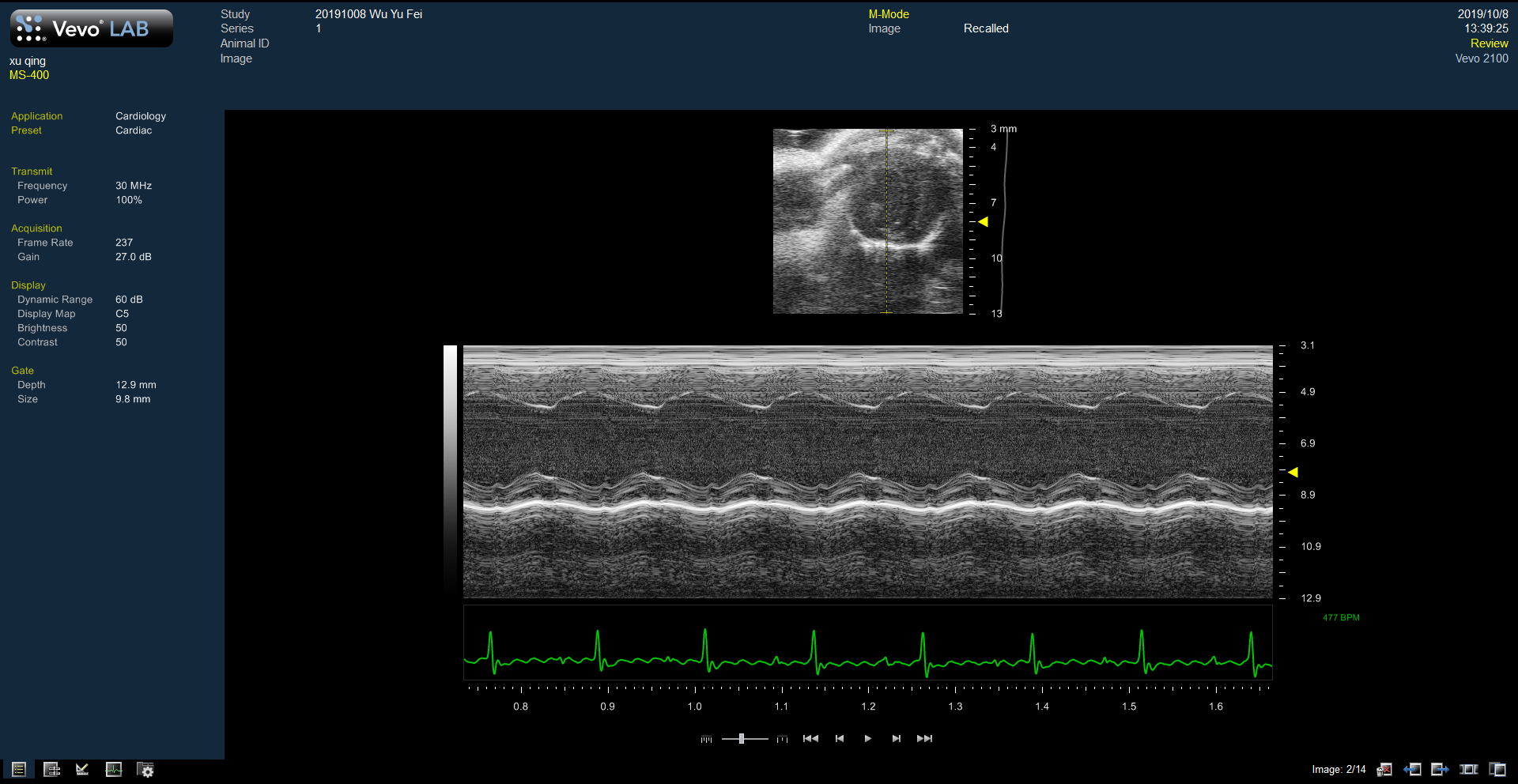

Supplement: Supplementary file 4 — Source data Fig. 2 [file 44321_2026_405_MOESM4_ESM.zip › Figure 2/E/NC-Sflfl.tif]

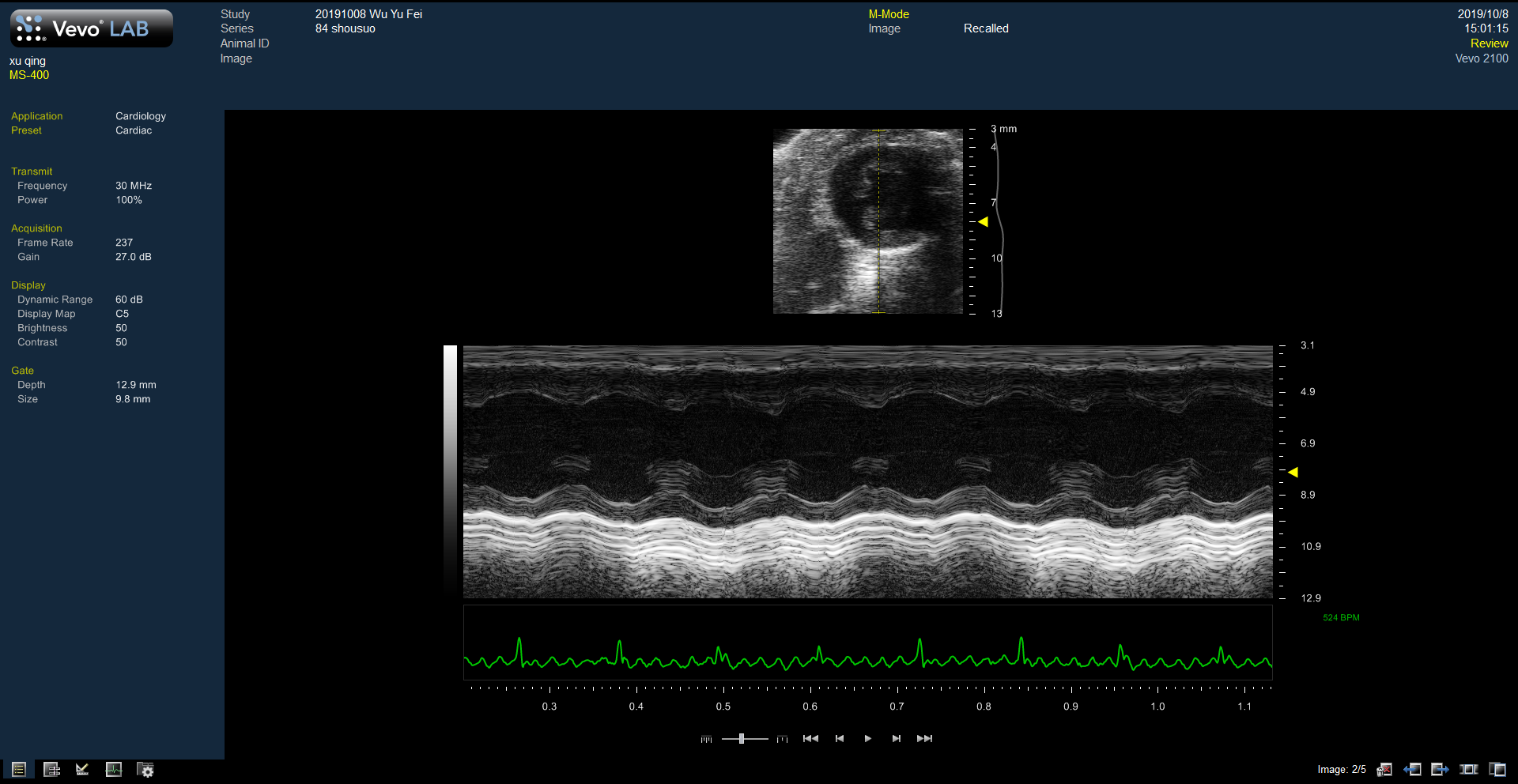

Supplement: Supplementary file 4 — Source data Fig. 2 [file 44321_2026_405_MOESM4_ESM.zip › Figure 2/E/HFpEF-SEC.tif]

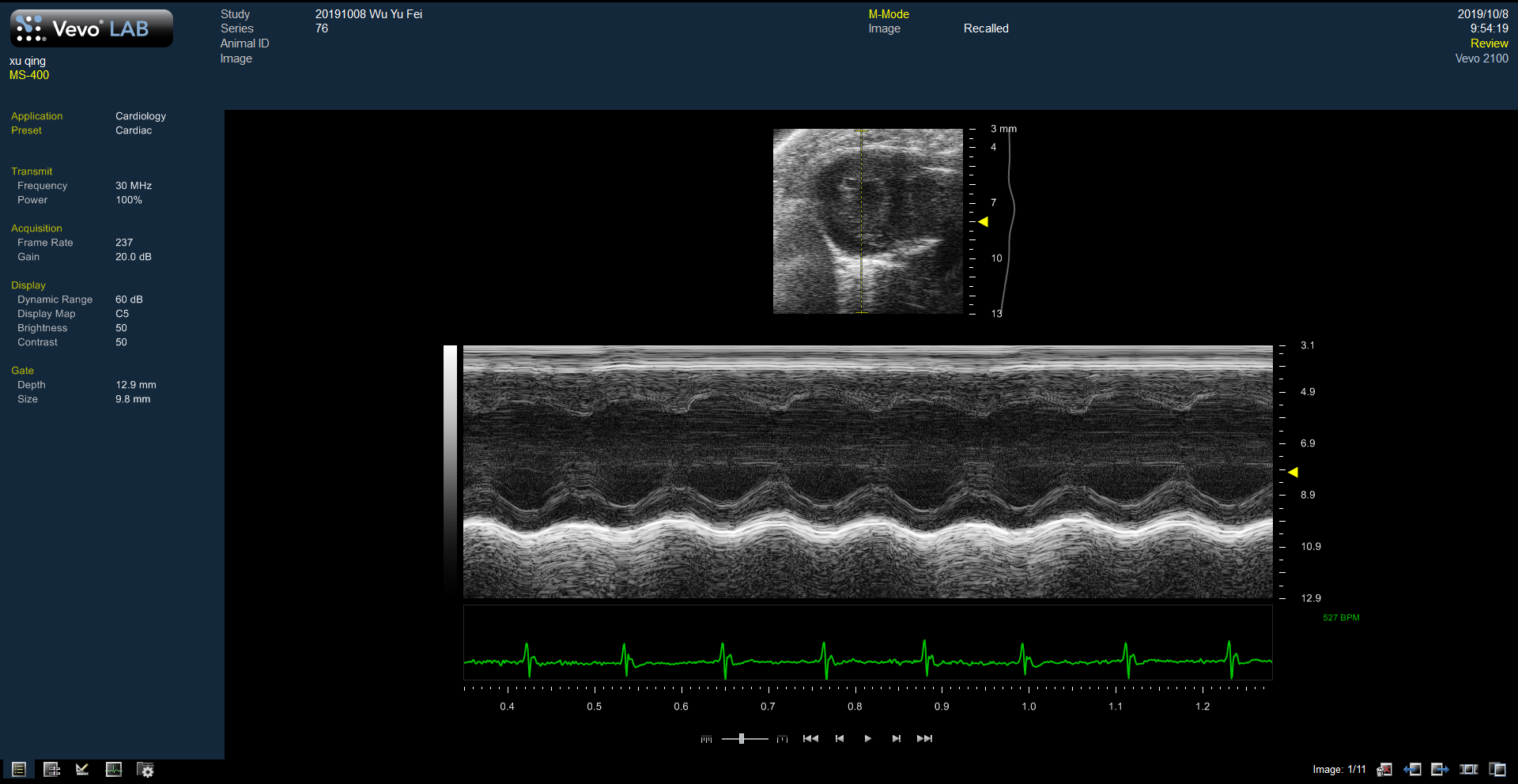

Supplement: Supplementary file 4 — Source data Fig. 2 [file 44321_2026_405_MOESM4_ESM.zip › Figure 2/E/HFpEF-Sflfl.tif]

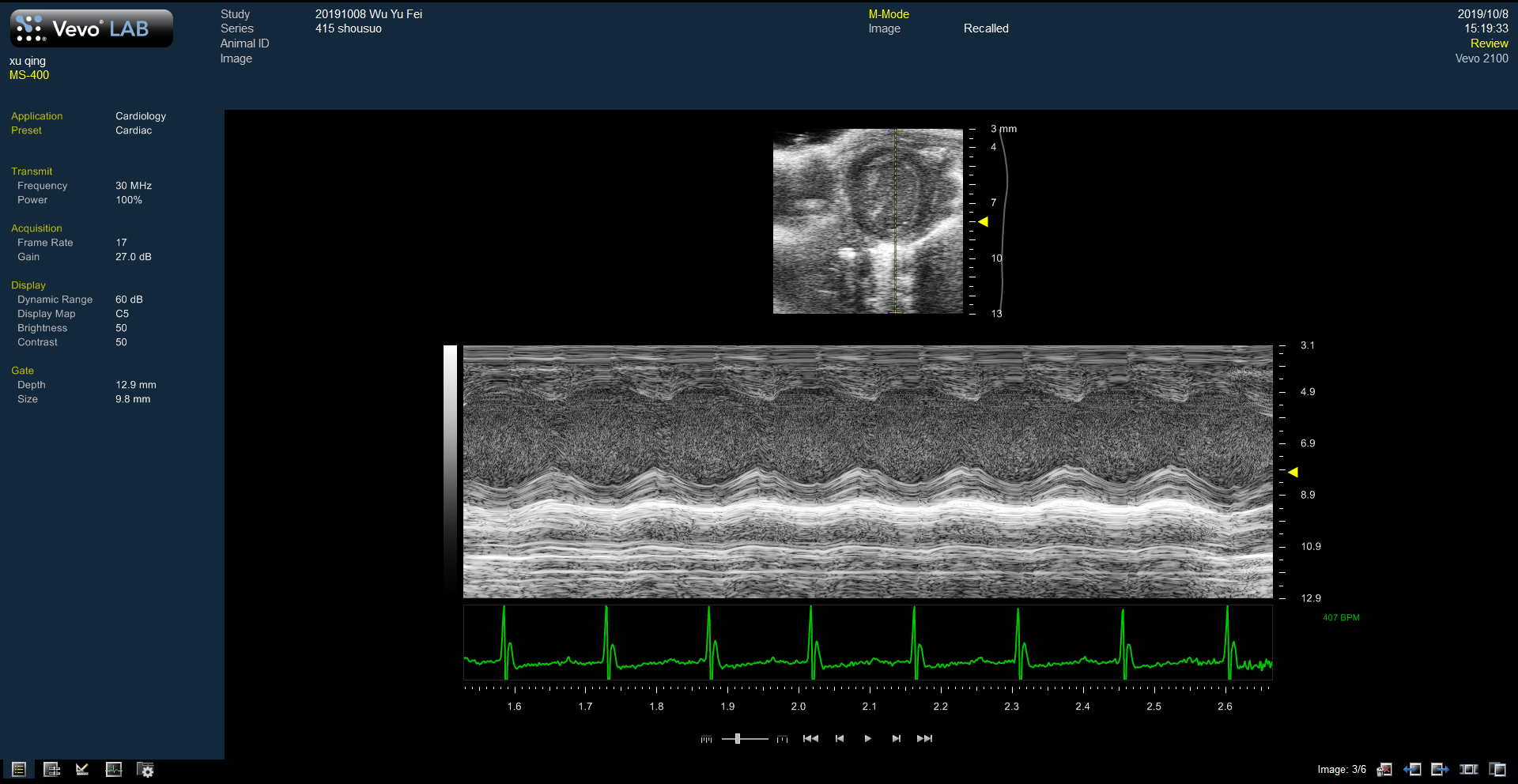

Supplement: Supplementary file 4 — Source data Fig. 2 [file 44321_2026_405_MOESM4_ESM.zip › Figure 2/E/NC-SEC.tif]

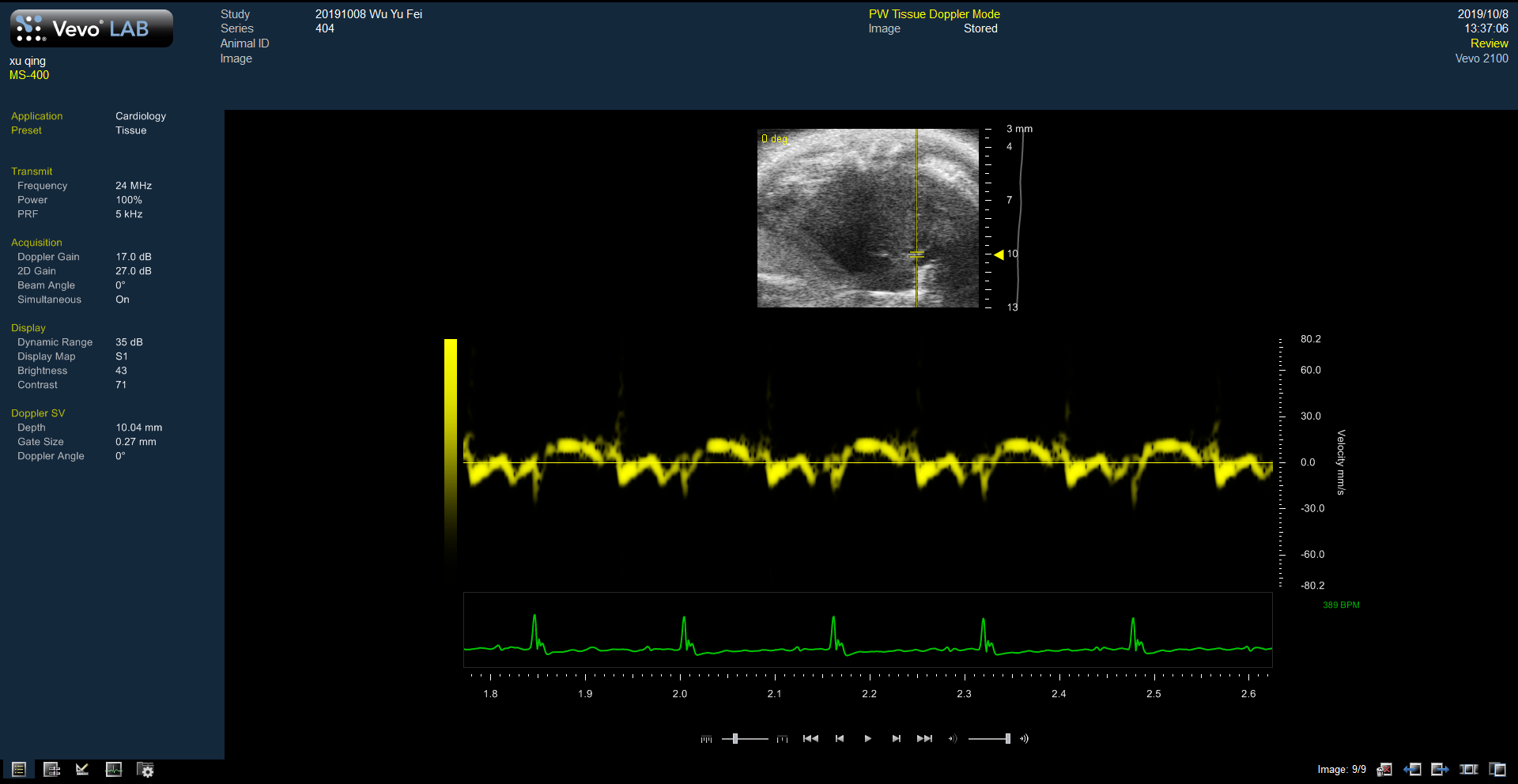

Supplement: Supplementary file 4 — Source data Fig. 2 [file 44321_2026_405_MOESM4_ESM.zip › Figure 2/M/Tissue Doppler tracing/NC-Sflfl.tif]

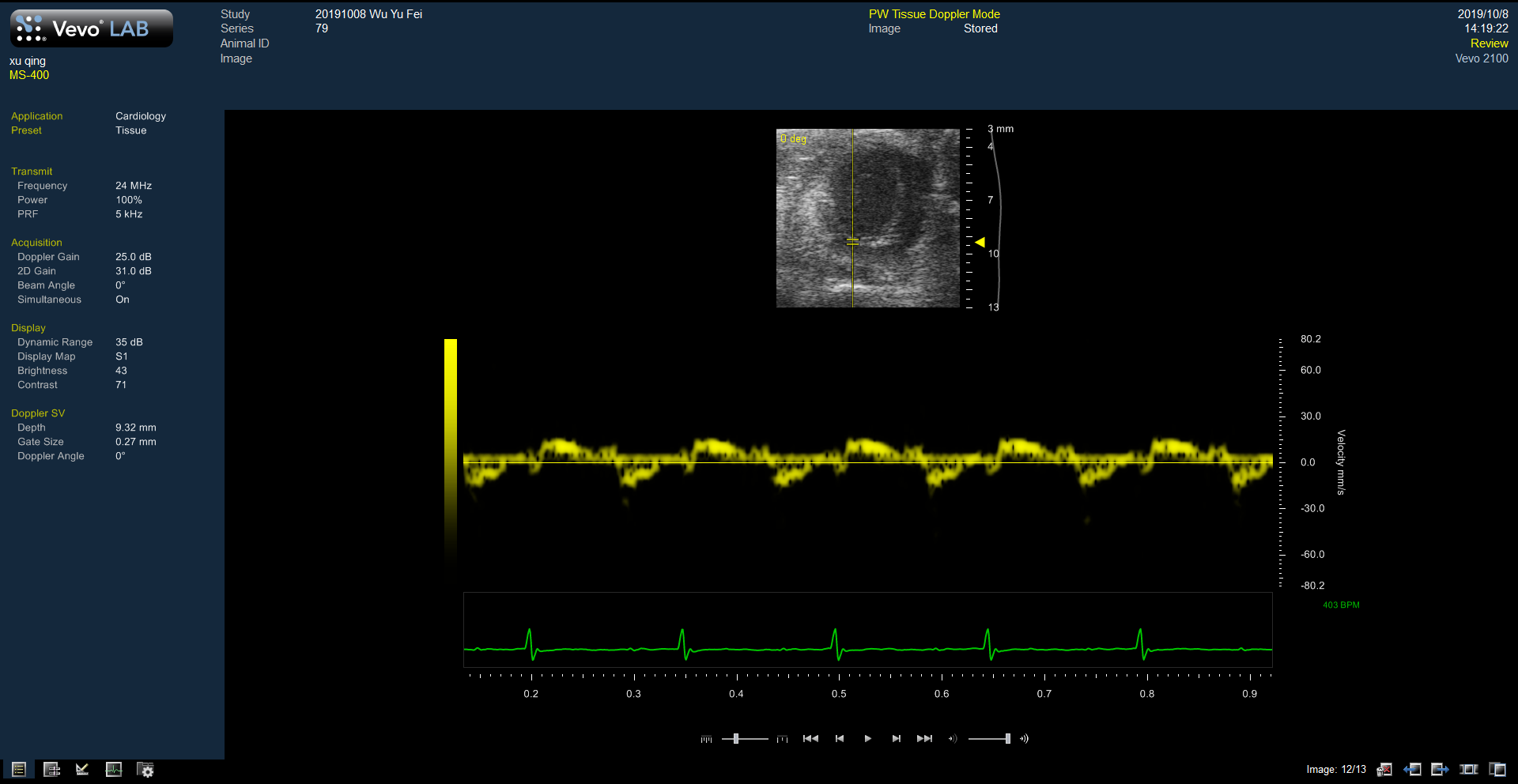

Supplement: Supplementary file 4 — Source data Fig. 2 [file 44321_2026_405_MOESM4_ESM.zip › Figure 2/M/Tissue Doppler tracing/HFpEF-SEC.tif]

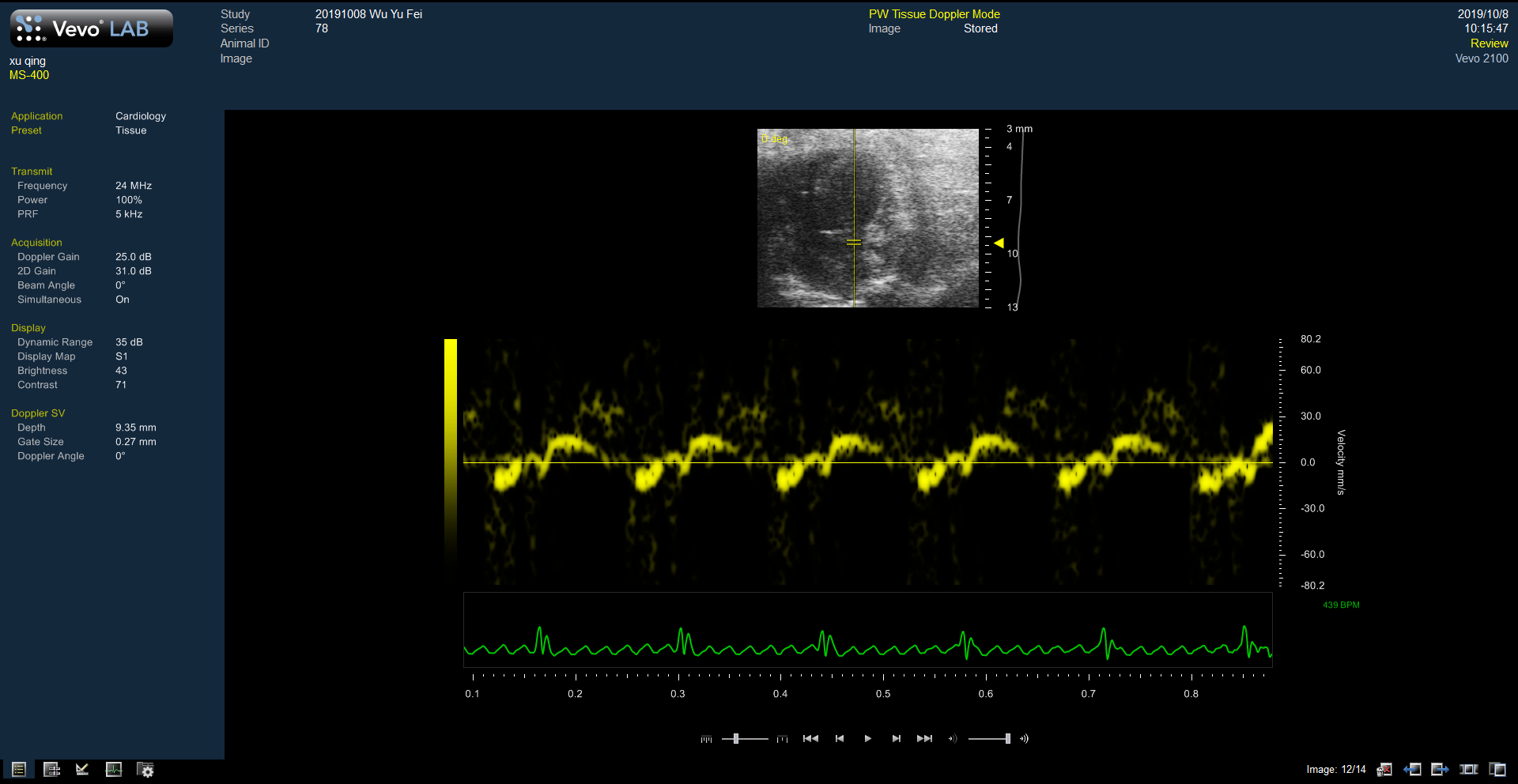

Supplement: Supplementary file 4 — Source data Fig. 2 [file 44321_2026_405_MOESM4_ESM.zip › Figure 2/M/Tissue Doppler tracing/HFpEF-Sflfl.tif]

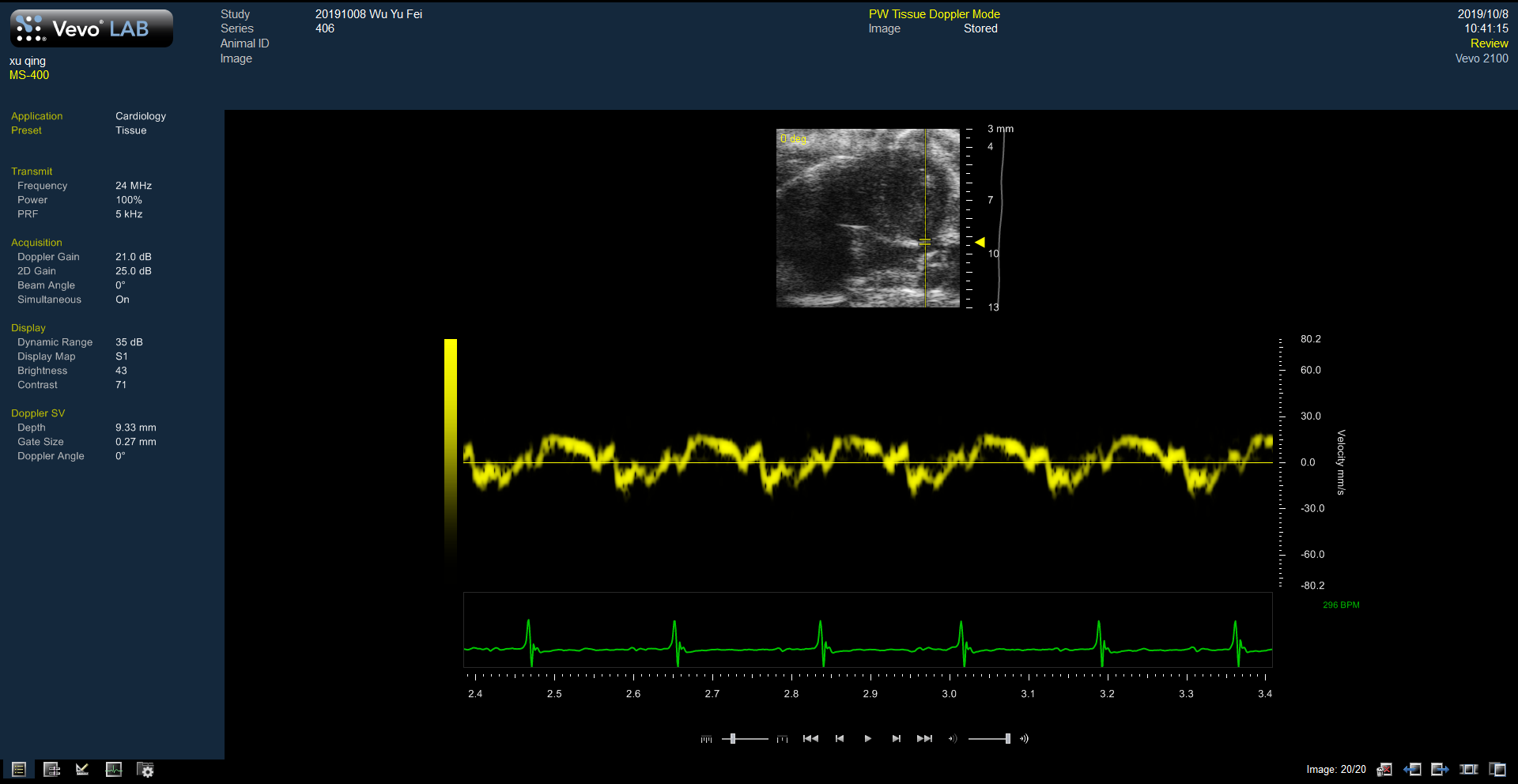

Supplement: Supplementary file 4 — Source data Fig. 2 [file 44321_2026_405_MOESM4_ESM.zip › Figure 2/M/Tissue Doppler tracing/NC-SEC.tif]

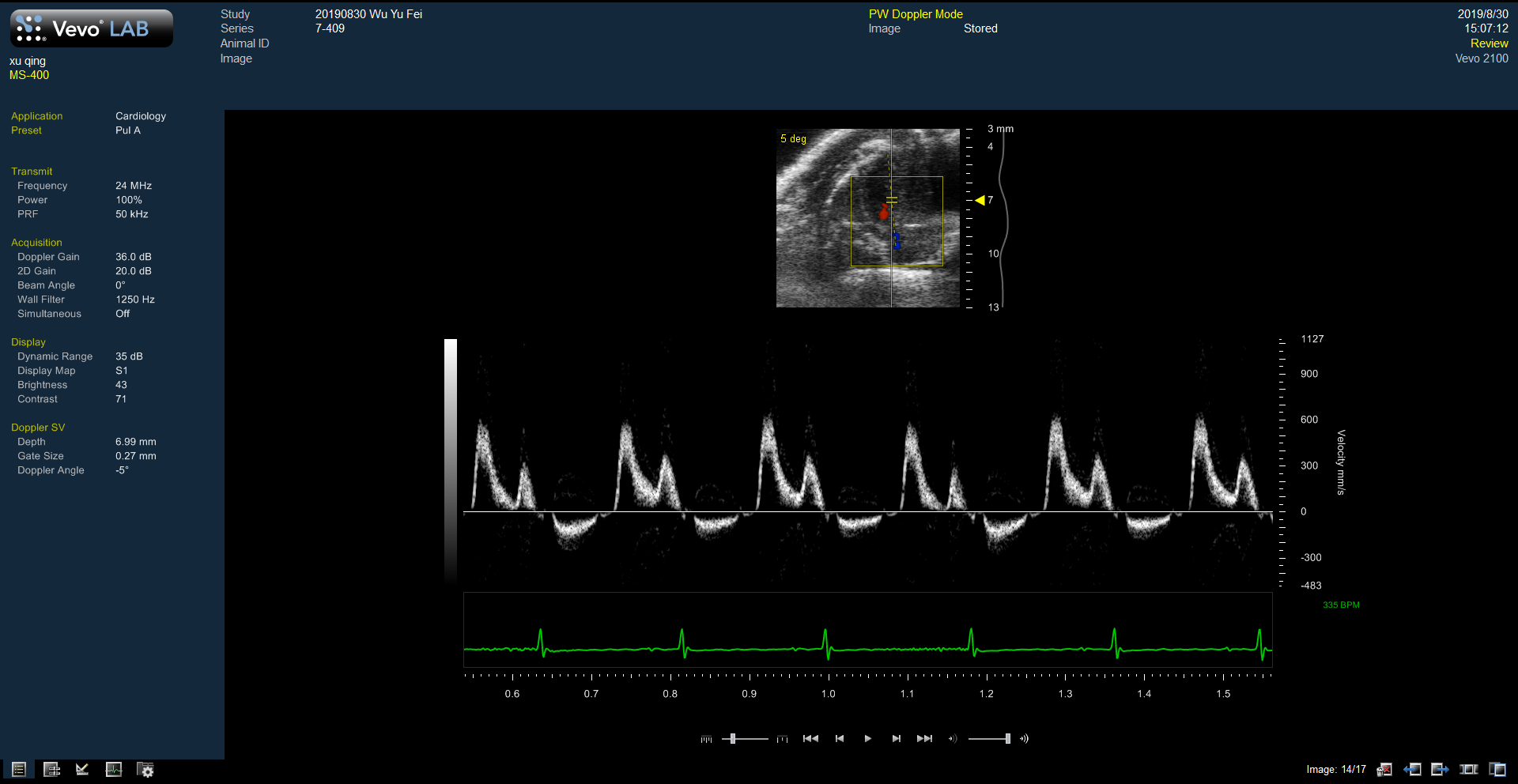

Supplement: Supplementary file 4 — Source data Fig. 2 [file 44321_2026_405_MOESM4_ESM.zip › Figure 2/M/Pulsed-wave Doppler waveform/NC-Sflfl.tif]

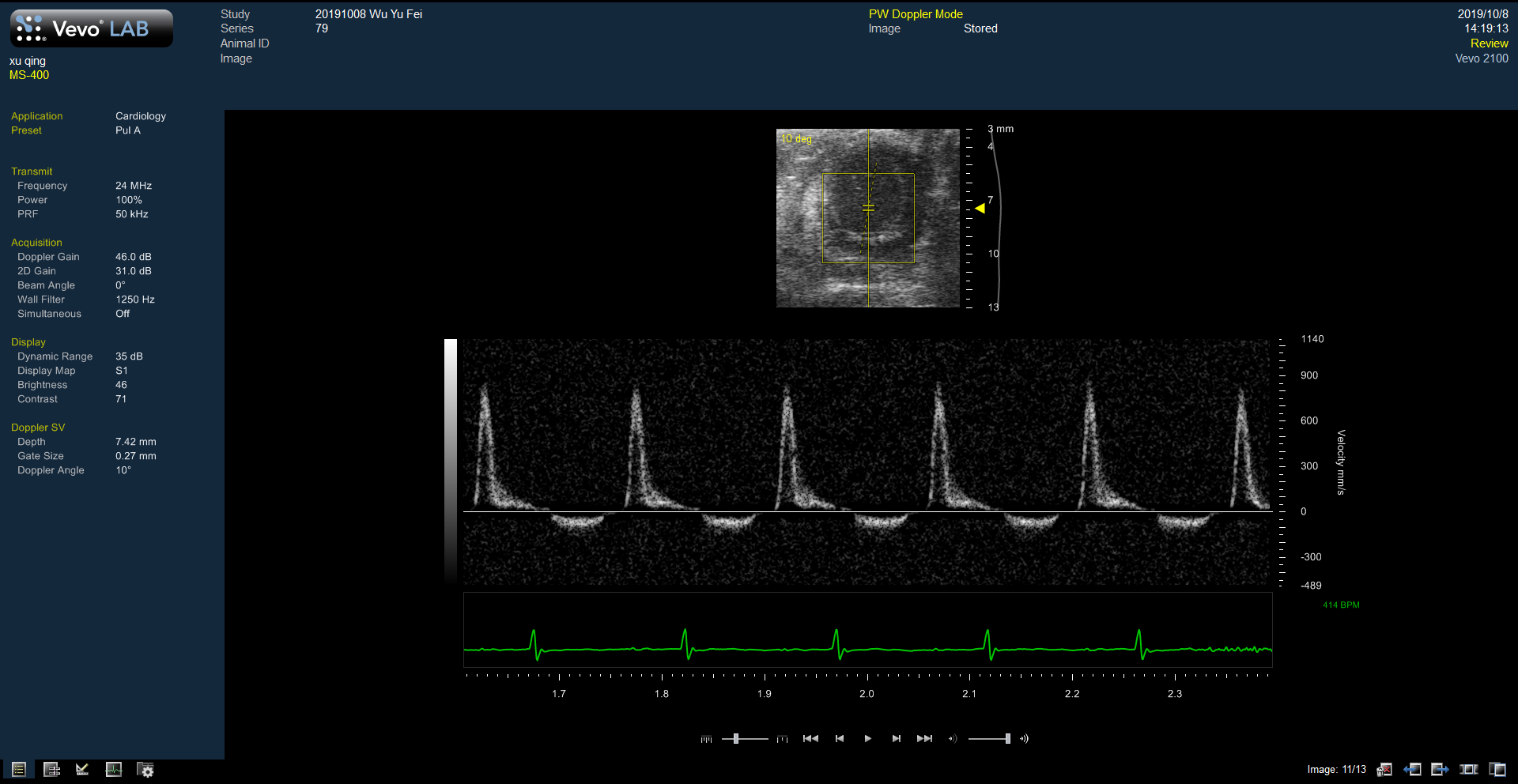

Supplement: Supplementary file 4 — Source data Fig. 2 [file 44321_2026_405_MOESM4_ESM.zip › Figure 2/M/Pulsed-wave Doppler waveform/HFpEF-SEC.tif]

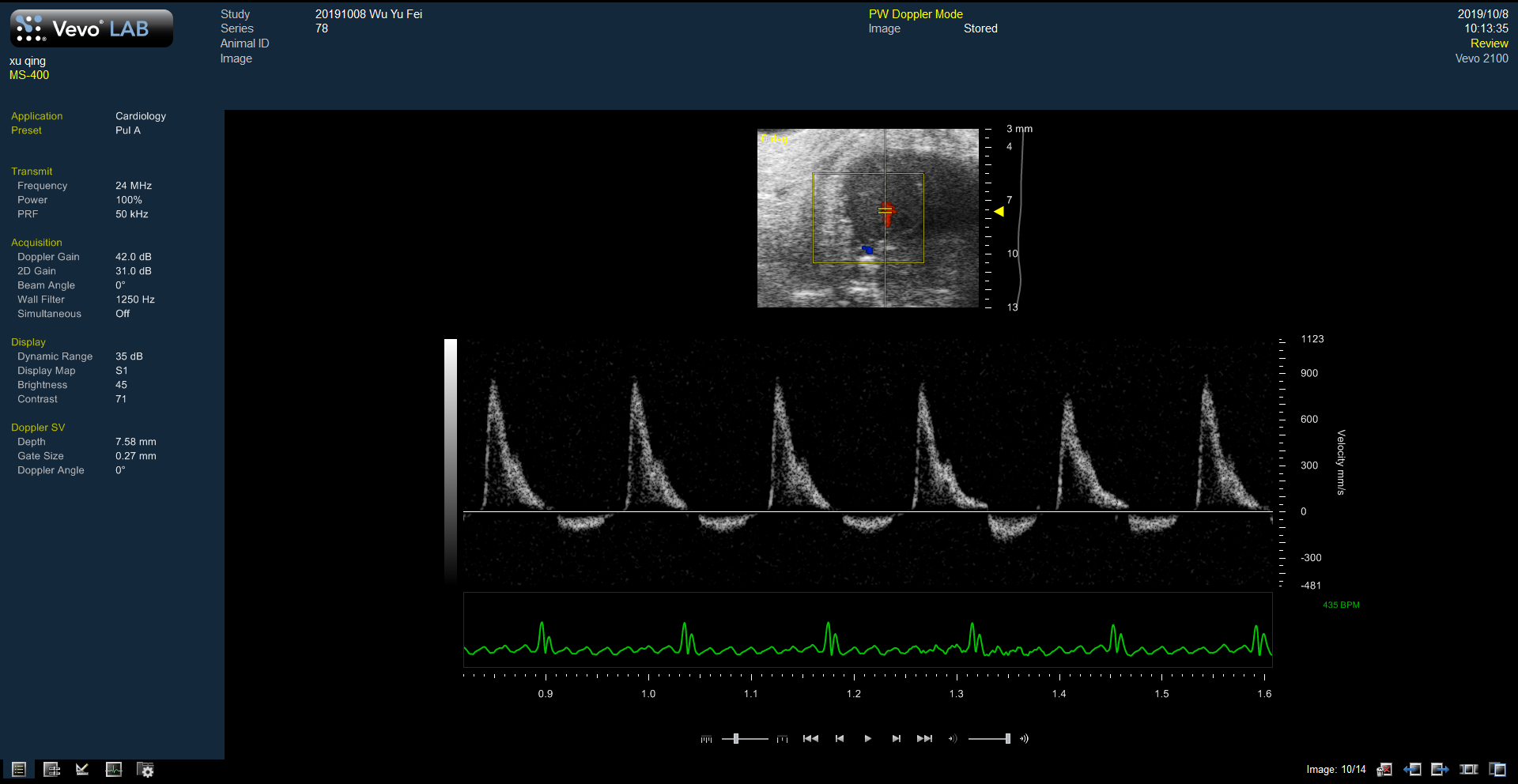

Supplement: Supplementary file 4 — Source data Fig. 2 [file 44321_2026_405_MOESM4_ESM.zip › Figure 2/M/Pulsed-wave Doppler waveform/HFpEF-Sflfl.tif]

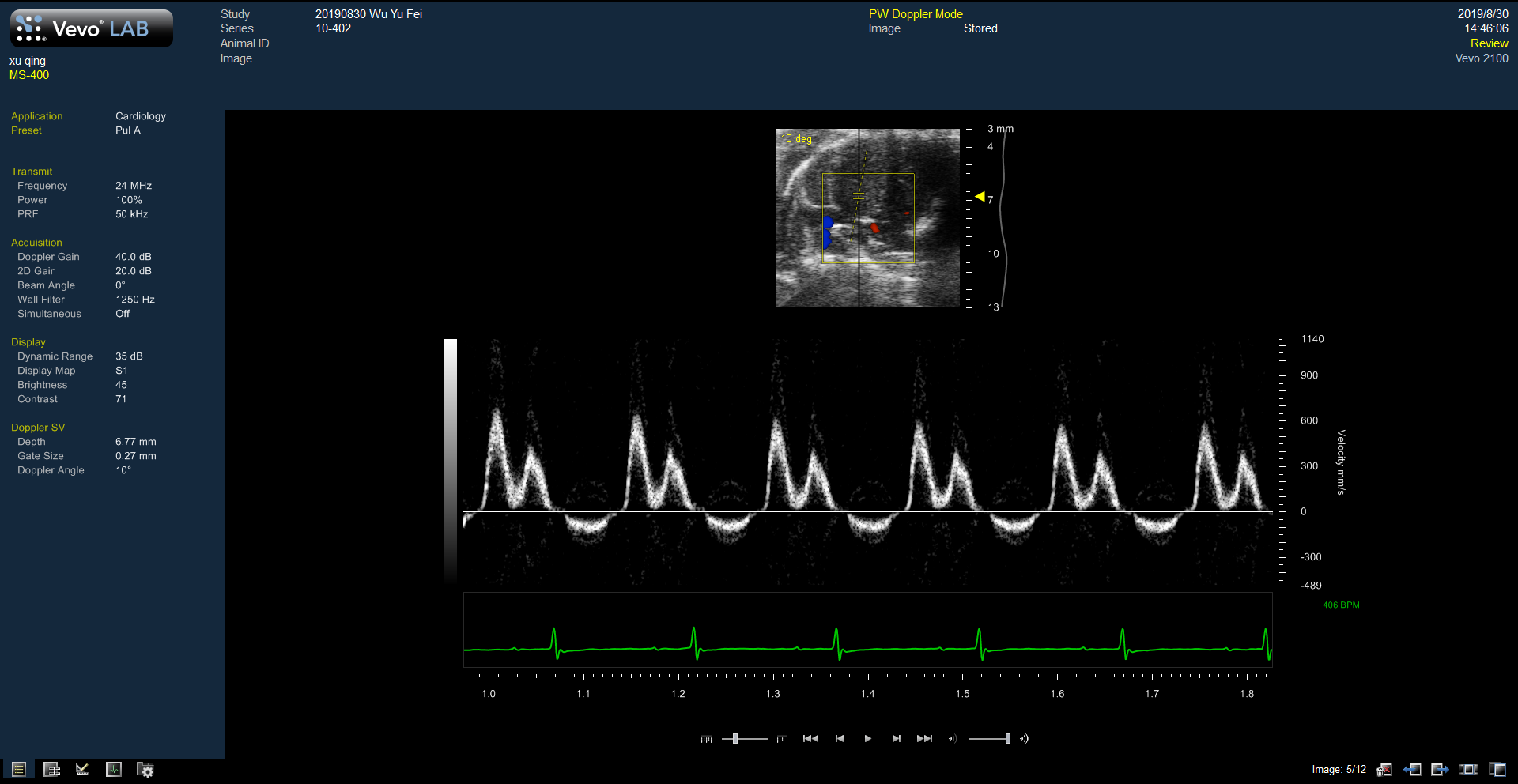

Supplement: Supplementary file 4 — Source data Fig. 2 [file 44321_2026_405_MOESM4_ESM.zip › Figure 2/M/Pulsed-wave Doppler waveform/NC-SEC.tif]

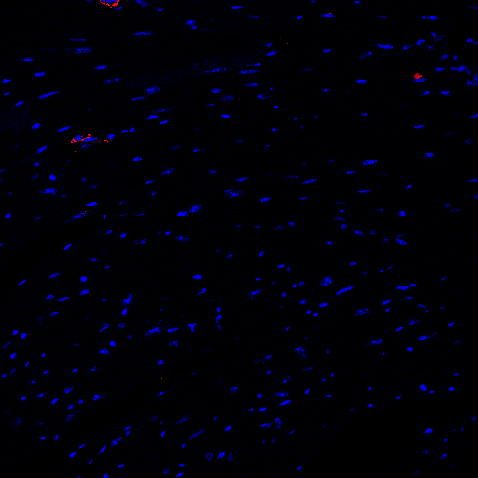

Supplement: Supplementary file 5 — Source data Fig. 3 [file 44321_2026_405_MOESM5_ESM.zip › Figure 3/F/NC-Sflfl.tif]

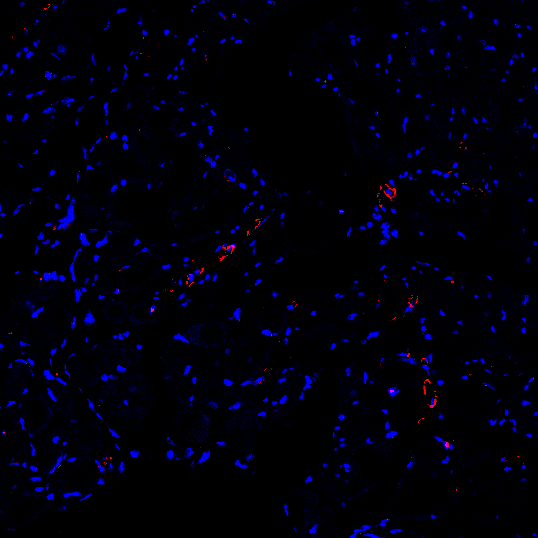

Supplement: Supplementary file 5 — Source data Fig. 3 [file 44321_2026_405_MOESM5_ESM.zip › Figure 3/F/HFpEF-Sflfl.tif]

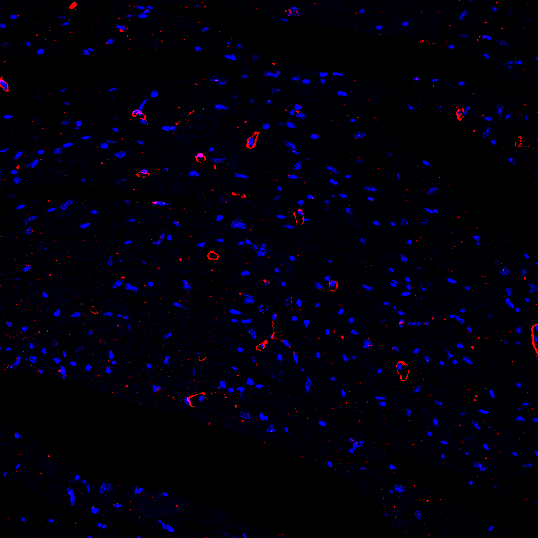

Supplement: Supplementary file 5 — Source data Fig. 3 [file 44321_2026_405_MOESM5_ESM.zip › Figure 3/F/HFpEF-SEC-merge.tif]

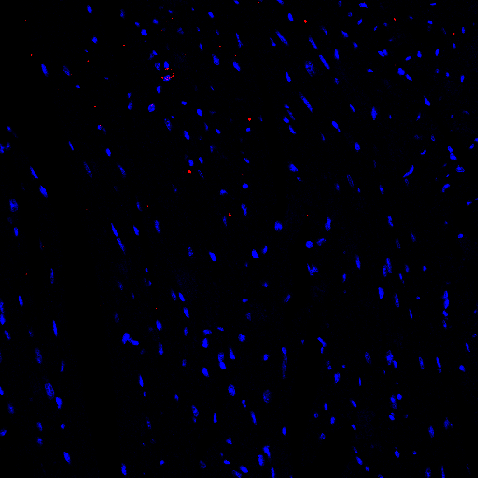

Supplement: Supplementary file 5 — Source data Fig. 3 [file 44321_2026_405_MOESM5_ESM.zip › Figure 3/F/NC-SEC.tif]

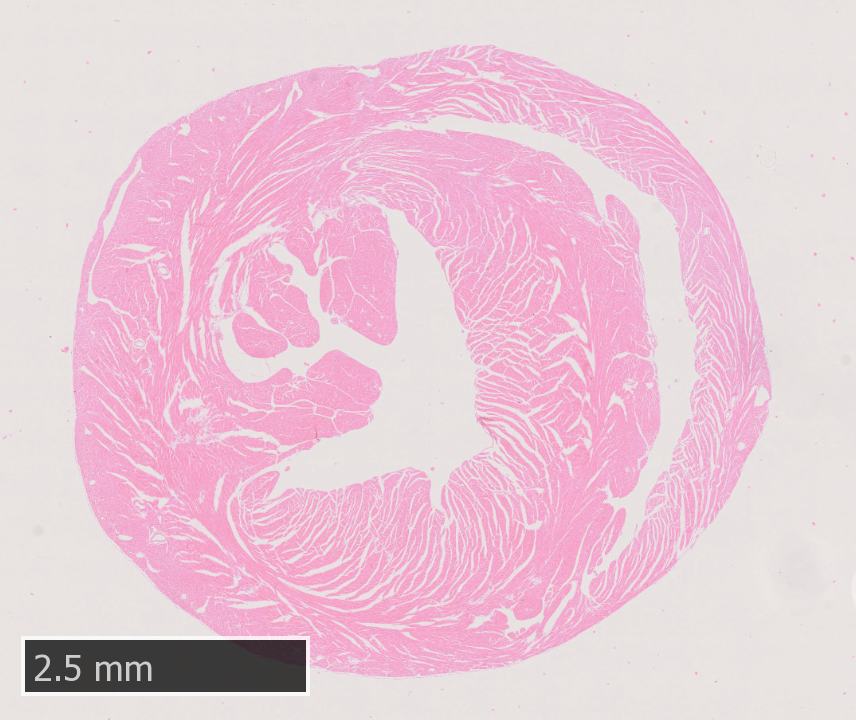

Supplement: Supplementary file 5 — Source data Fig. 3 [file 44321_2026_405_MOESM5_ESM.zip › Figure 3/C/H&E/HFpEF-SEC ∩╝êwith scale bar∩╝ë.tif]

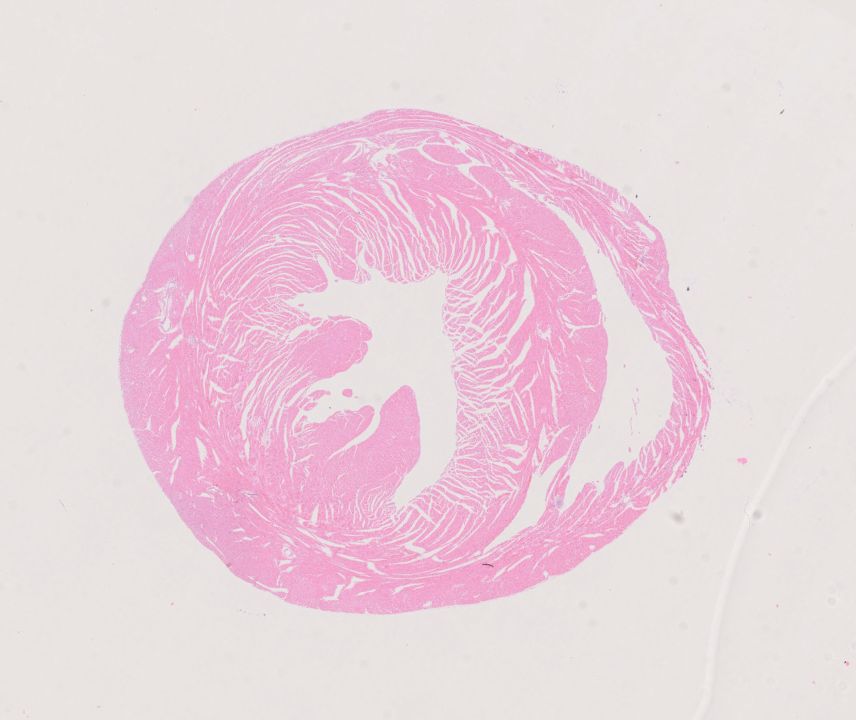

Supplement: Supplementary file 5 — Source data Fig. 3 [file 44321_2026_405_MOESM5_ESM.zip › Figure 3/C/H&E/NC-Sflfl.tif]

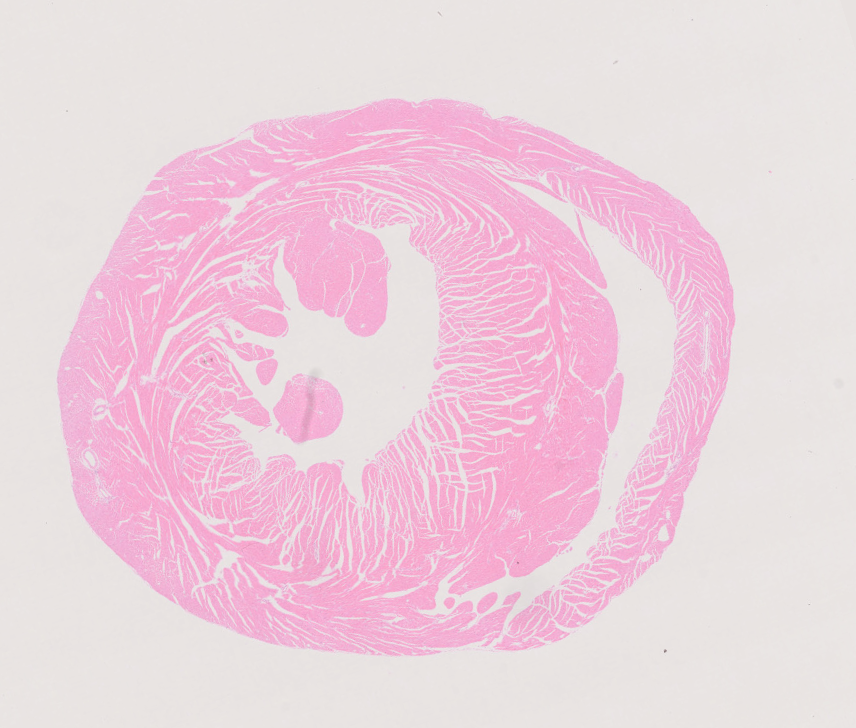

Supplement: Supplementary file 5 — Source data Fig. 3 [file 44321_2026_405_MOESM5_ESM.zip › Figure 3/C/H&E/HFpEF-Sflfl.tif]

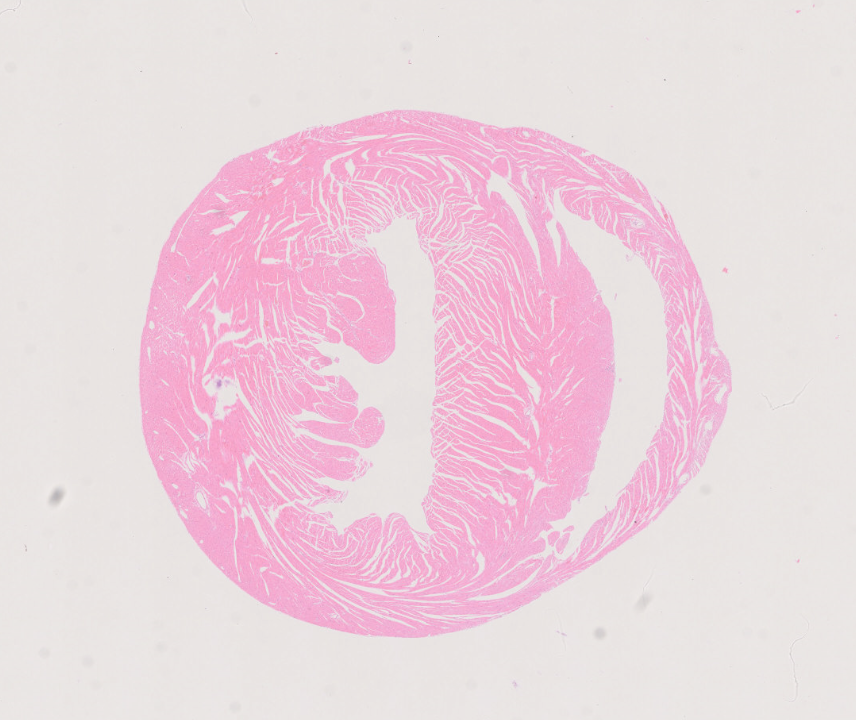

Supplement: Supplementary file 5 — Source data Fig. 3 [file 44321_2026_405_MOESM5_ESM.zip › Figure 3/C/H&E/NC-SEC.tif]

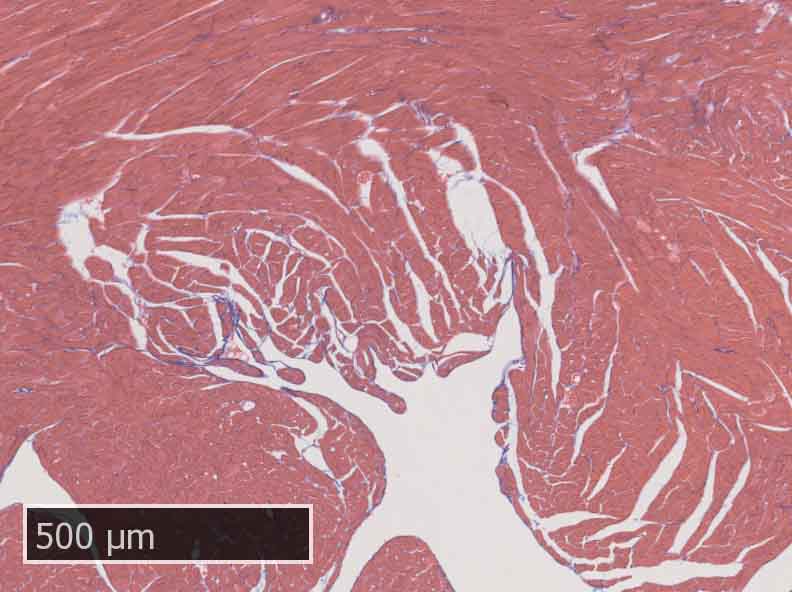

Supplement: Supplementary file 5 — Source data Fig. 3 [file 44321_2026_405_MOESM5_ESM.zip › Figure 3/C/Masson/NC-SEC (with Scale bar).jpg]

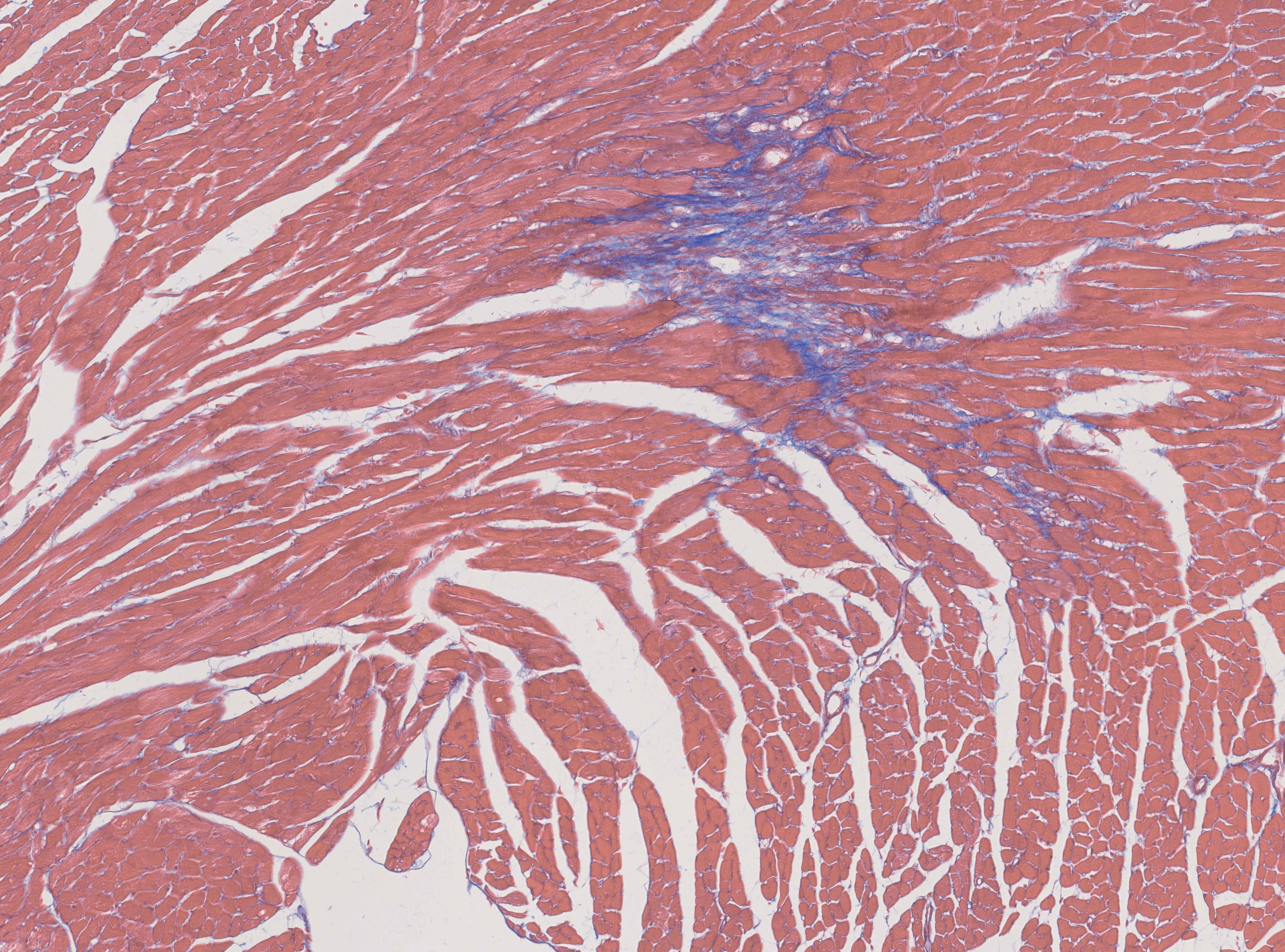

Supplement: Supplementary file 5 — Source data Fig. 3 [file 44321_2026_405_MOESM5_ESM.zip › Figure 3/C/Masson/HFpEF-Sflfl.jpg]

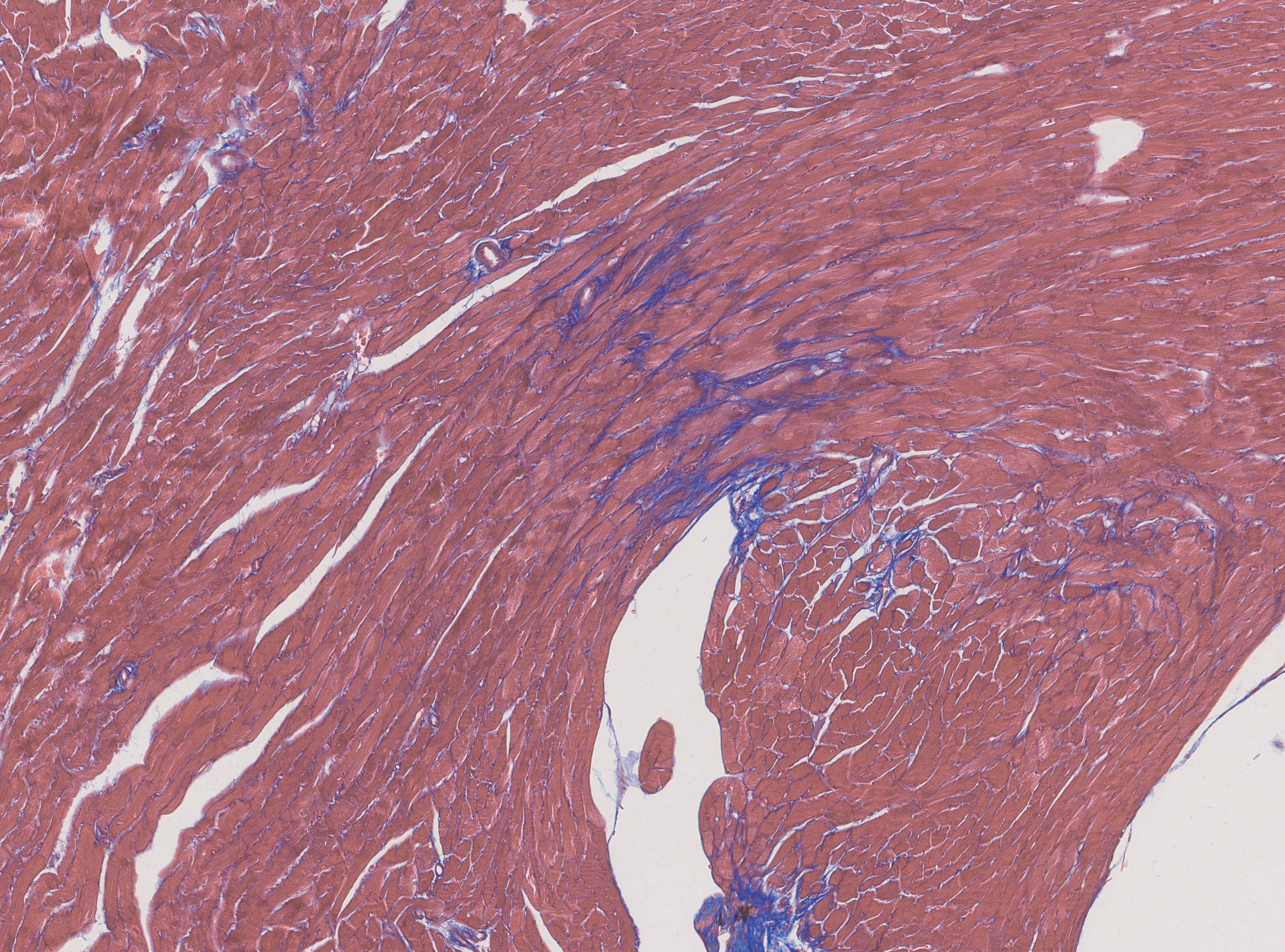

Supplement: Supplementary file 5 — Source data Fig. 3 [file 44321_2026_405_MOESM5_ESM.zip › Figure 3/C/Masson/HFpEF-SEC.jpg]

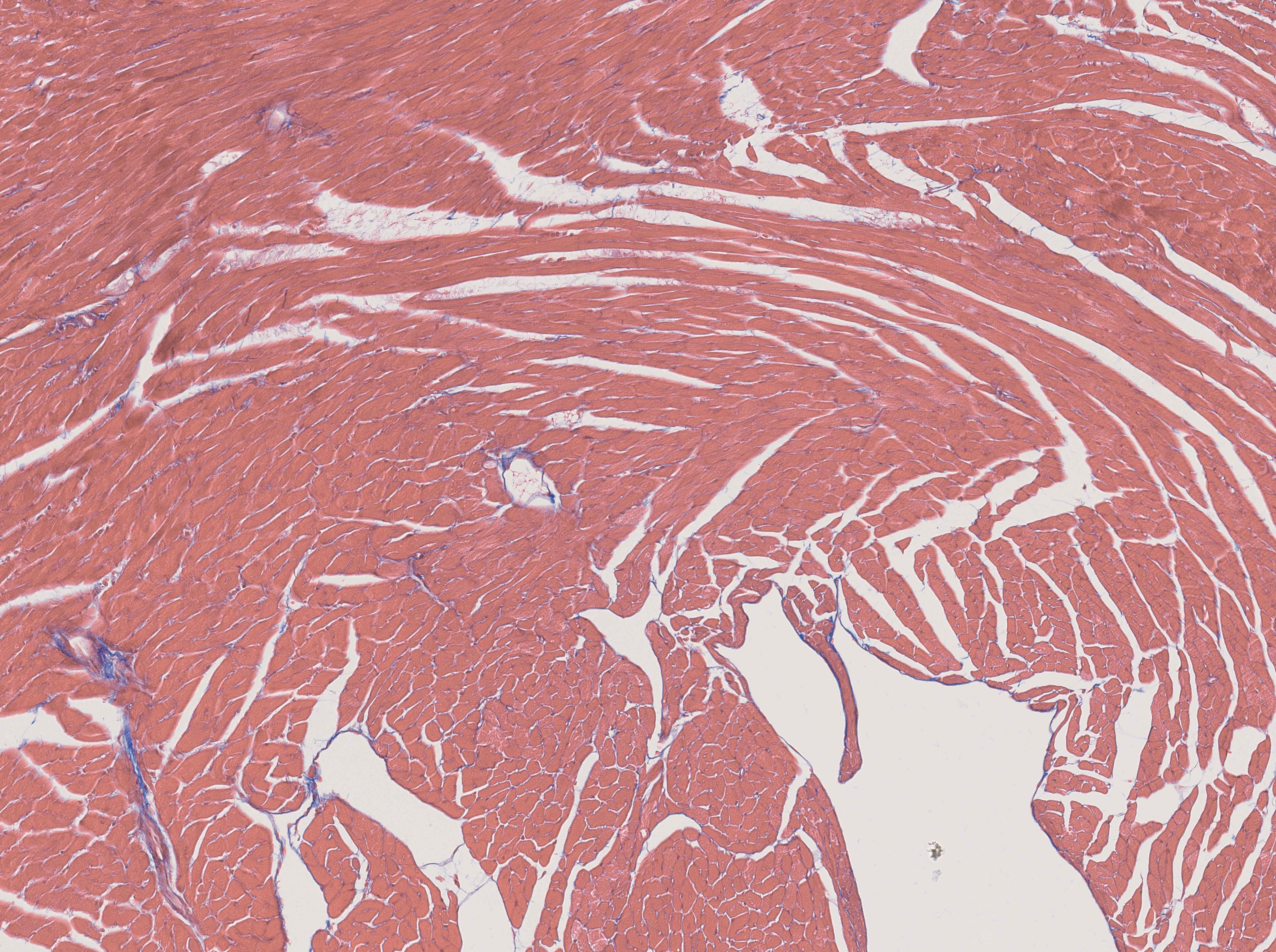

Supplement: Supplementary file 5 — Source data Fig. 3 [file 44321_2026_405_MOESM5_ESM.zip › Figure 3/C/Masson/NC-Sflfl.jpg]

# BD FACSDiva 9.0.2

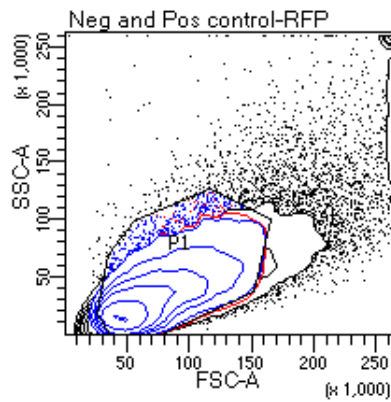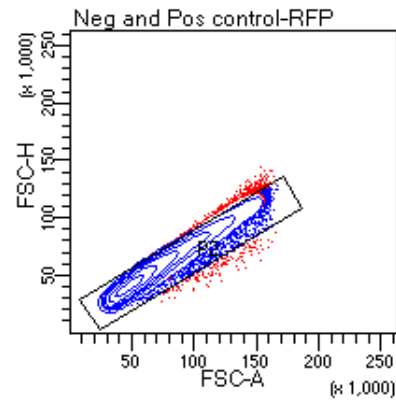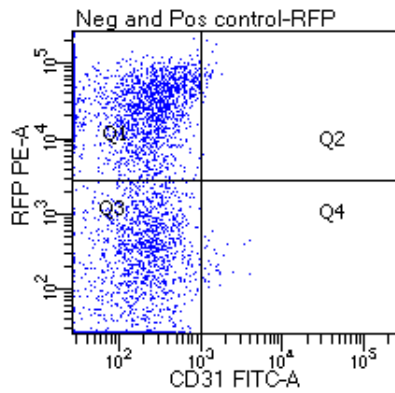

| Tube: RFP    |         |         |        |
|--------------|---------|---------|--------|
| Population   | #Events | %Parent | %Total |
| ■ All Events | 17,793  | ####    | 100.0  |
| ■ P1         | 11,749  | 66.0    | 66.0   |
| ■ P2         | 10,864  | 92.5    | 61.1   |
| ☒ Q1         | 5,915   | 54.4    | 33.2   |
| ☒ Q2         | 73      | 0.7     | 0.4    |
| ☒ Q3         | 4,792   | 44.1    | 26.9   |
| ☒ Q4         | 84      | 0.8     | 0.5    |

Supplement: Supplementary file 6 — Source data Fig. 4 [file 44321_2026_405_MOESM6_ESM.zip › Figure 4/G/RFP-positive control.pdf]

# BD FACSDiva 9.0.2

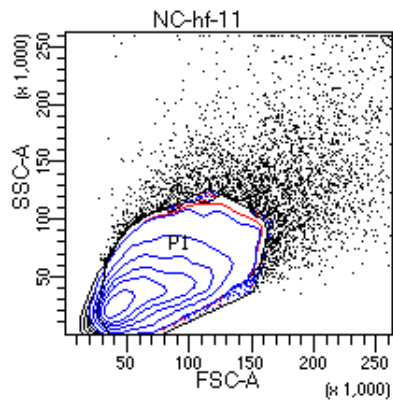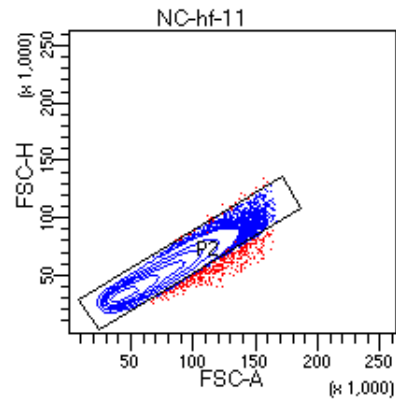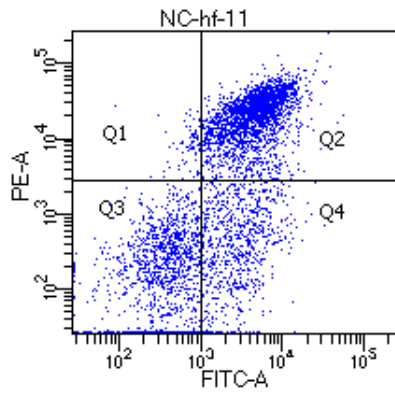

| Tube: 11     |         |         |        |
|--------------|---------|---------|--------|
| Population   | #Events | %Parent | %Total |
| ■ All Events | 23,080  | ####    | 100.0  |
| ■ P1         | 18,276  | 79.2    | 79.2   |
| ■ P2         | 17,842  | 97.6    | 77.3   |
| ☒ Q1         | 333     | 1.9     | 1.4    |
| ☒ Q2         | 9,589   | 53.7    | 41.5   |
| ☒ Q3         | 4,826   | 27.0    | 20.9   |
| ☒ Q4         | 3,094   | 17.3    | 13.4   |

Supplement: Supplementary file 6 — Source data Fig. 4 [file 44321_2026_405_MOESM6_ESM.zip › Figure 4/G/HFpEF-Sfl+ and REC.pdf]

# BD FACSDiva 9.0.2

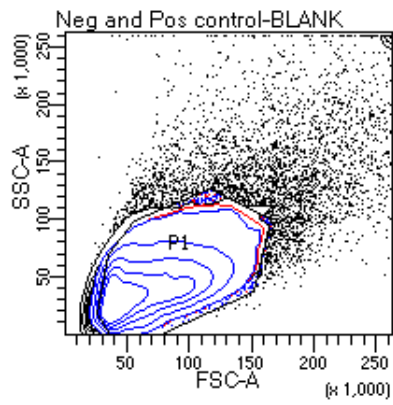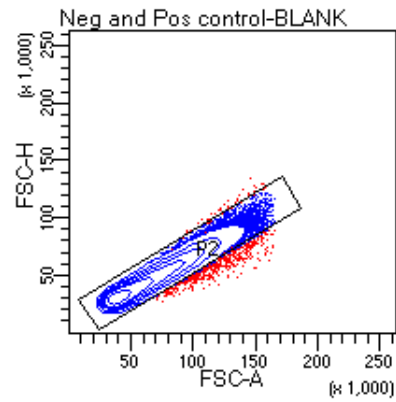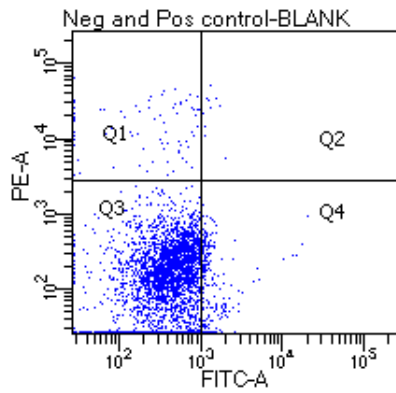

| Tube: BLANK  |         |         |        |
|--------------|---------|---------|--------|
| Population   | #Events | %Parent | %Total |
| ■ All Events | 25,368  | ####    | 100.0  |
| ■ P1         | 18,347  | 72.3    | 72.3   |
| ■ P2         | 17,853  | 97.3    | 70.4   |
| ☒ Q1         | 448     | 2.5     | 1.8    |
| ☒ Q2         | 36      | 0.2     | 0.1    |
| ☒ Q3         | 16,249  | 91.0    | 64.1   |
| ☒ Q4         | 1,120   | 6.3     | 4.4    |

Supplement: Supplementary file 6 — Source data Fig. 4 [file 44321_2026_405_MOESM6_ESM.zip › Figure 4/G/Negative control.pdf]

# BD FACSDiva 9.0.2

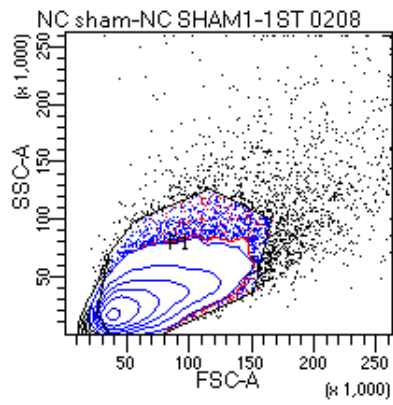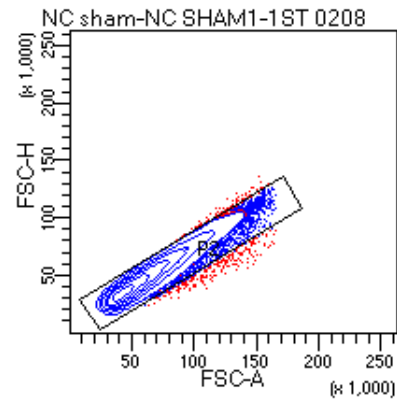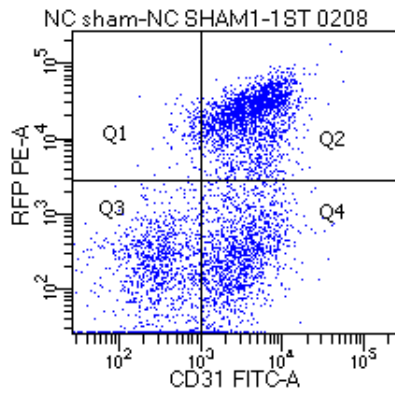

| Tube: NC SHAM1-1ST 0208 |         |         |        |
|-------------------------|---------|---------|--------|
| Population              | #Events | %Parent | %Total |
| ■ All Events            | 17,241  | ####    | 100.0  |
| ■ P1                    | 13,884  | 80.5    | 80.5   |
| ■ P2                    | 13,490  | 97.2    | 78.2   |
| ☒ Q1                    | 301     | 2.2     | 1.7    |
| ☒ Q2                    | 5,855   | 43.4    | 34.0   |
| ☒ Q3                    | 3,417   | 25.3    | 19.8   |
| ☒ Q4                    | 3,917   | 29.0    | 22.7   |

Supplement: Supplementary file 6 — Source data Fig. 4 [file 44321_2026_405_MOESM6_ESM.zip › Figure 4/G/NC-Sflfl and REC.pdf]

# BD FACSDiva 9.0.2

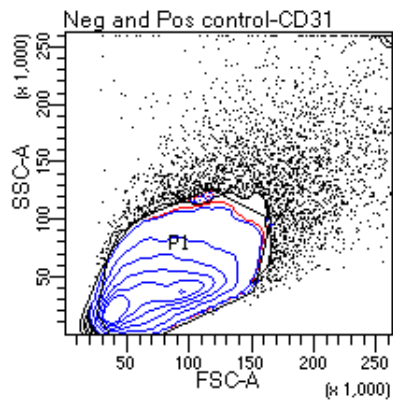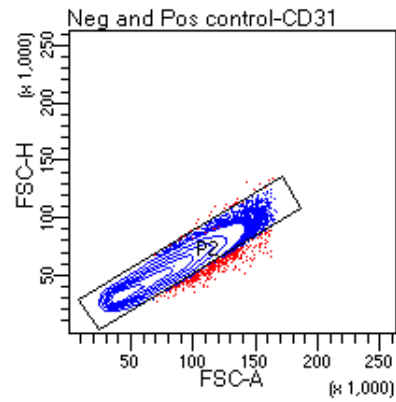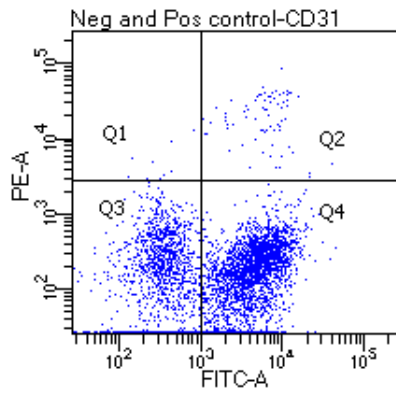

| Tube: CD31   |         |         |        |
|--------------|---------|---------|--------|
| Population   | #Events | %Parent | %Total |
| ■ All Events | 21,289  | ####    | 100.0  |
| ■ P1         | 16,586  | 77.9    | 77.9   |
| ■ P2         | 16,220  | 97.8    | 76.2   |
| ☒ Q1         | 43      | 0.3     | 0.2    |
| ☒ Q2         | 372     | 2.3     | 1.7    |
| ☒ Q3         | 5,584   | 34.4    | 26.2   |
| ☒ Q4         | 10,221  | 63.0    | 48.0   |

Supplement: Supplementary file 6 — Source data Fig. 4 [file 44321_2026_405_MOESM6_ESM.zip › Figure 4/G/CD31-positive control.pdf]

# BD FACSDiva 9.0.2

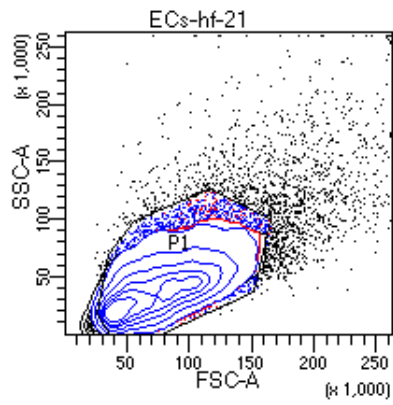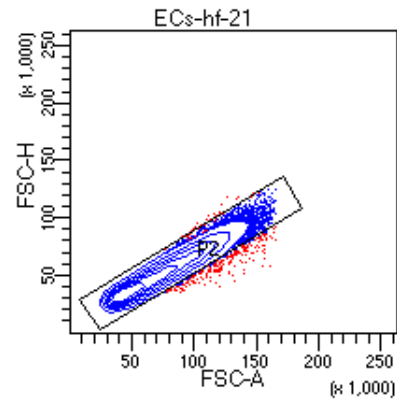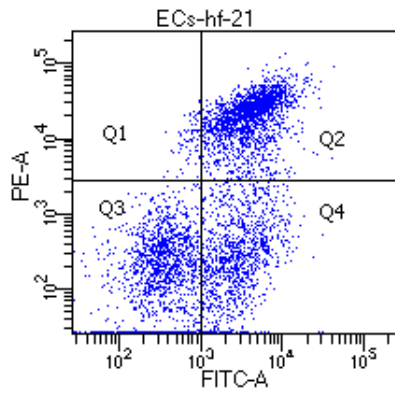

| Tube: 21     |         |         |        |
|--------------|---------|---------|--------|
| Population   | #Events | %Parent | %Total |
| ■ All Events | 16,219  | ####    | 100.0  |
| ■ P1         | 13,515  | 83.3    | 83.3   |
| ■ P2         | 13,248  | 98.0    | 81.7   |
| ☒ Q1         | 217     | 1.6     | 1.3    |
| ☒ Q2         | 5,460   | 41.2    | 33.7   |
| ☒ Q3         | 4,296   | 32.4    | 26.5   |
| ☒ Q4         | 3,275   | 24.7    | 20.2   |

Supplement: Supplementary file 6 — Source data Fig. 4 [file 44321_2026_405_MOESM6_ESM.zip › Figure 4/G/HFpEF-Sflfl and REC.pdf]

# BD FACSDiva 9.0.2

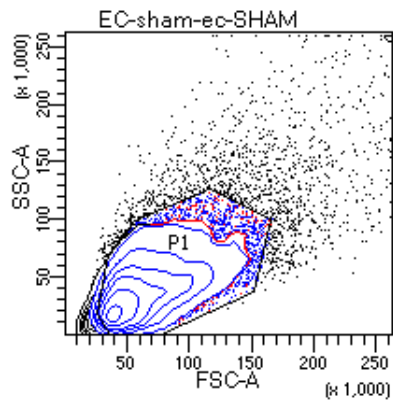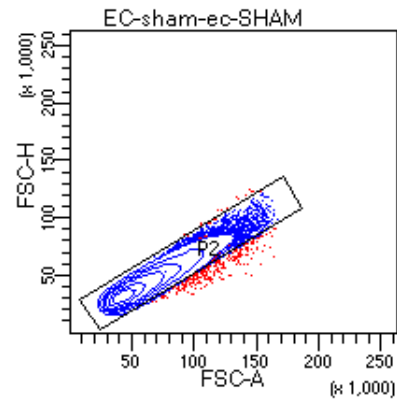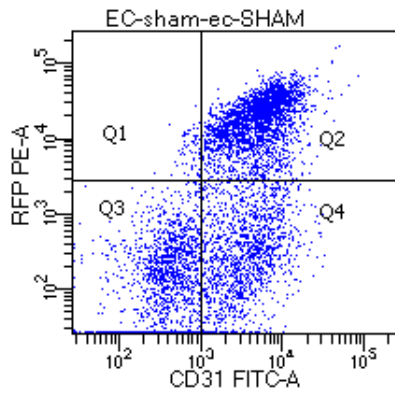

| Tube: ec-SHAM |         |         |        |
|---------------|---------|---------|--------|
| Population    | #Events | %Parent | %Total |
| ■ All Events  | 14,660  | ####    | 100.0  |
| ■ P1          | 11,595  | 79.1    | 79.1   |
| ■ P2          | 11,311  | 97.6    | 77.2   |
| ☒ Q1          | 147     | 1.3     | 1.0    |
| ☒ Q2          | 5,215   | 46.1    | 35.6   |
| ☒ Q3          | 2,984   | 26.4    | 20.4   |
| ☒ Q4          | 2,965   | 26.2    | 20.2   |

Supplement: Supplementary file 6 — Source data Fig. 4 [file 44321_2026_405_MOESM6_ESM.zip › Figure 4/G/NC-Sfl+ and REC.pdf]

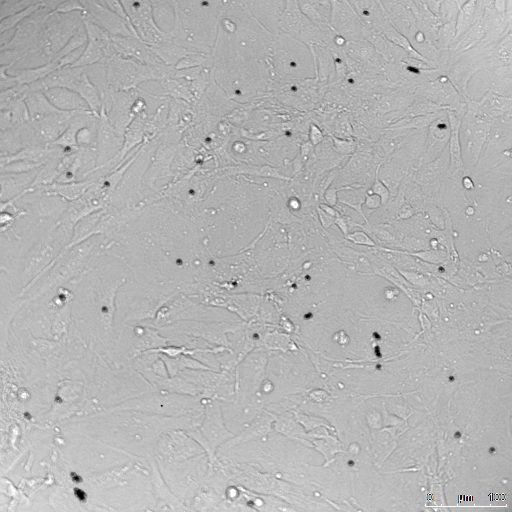

Supplement: Supplementary file 6 — Source data Fig. 4 [file 44321_2026_405_MOESM6_ESM.zip › Figure 4/A/TGF-b1.tif]

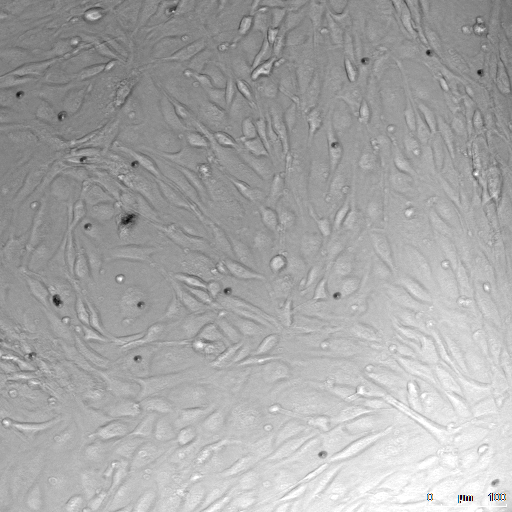

Supplement: Supplementary file 6 — Source data Fig. 4 [file 44321_2026_405_MOESM6_ESM.zip › Figure 4/A/Vehicle.tif]

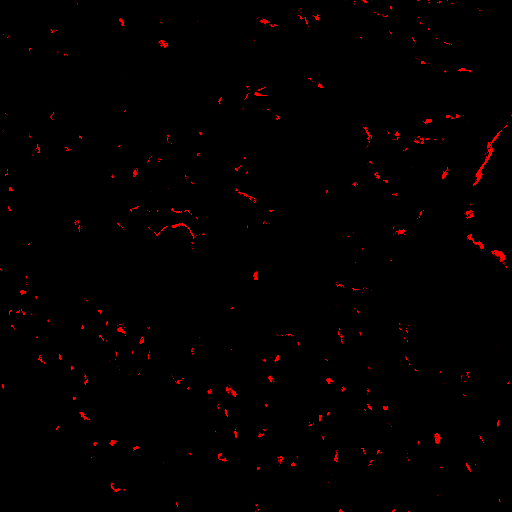

Supplement: Supplementary file 6 — Source data Fig. 4 [file 44321_2026_405_MOESM6_ESM.zip › Figure 4/F/RFP.tif]

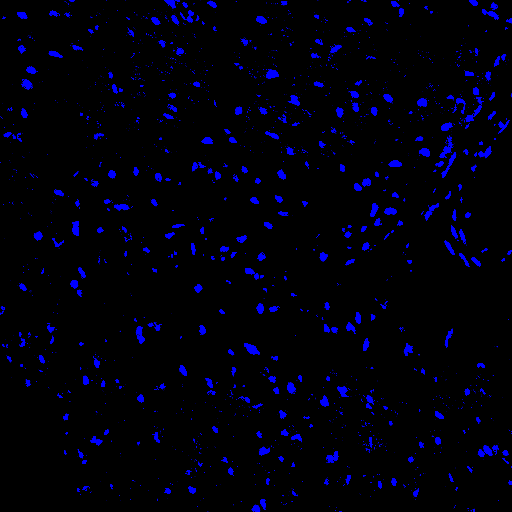

Supplement: Supplementary file 6 — Source data Fig. 4 [file 44321_2026_405_MOESM6_ESM.zip › Figure 4/F/DAPI.tif]

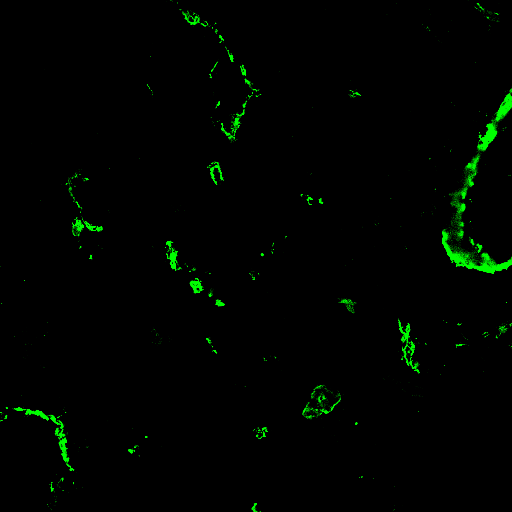

Supplement: Supplementary file 6 — Source data Fig. 4 [file 44321_2026_405_MOESM6_ESM.zip › Figure 4/F/aSMA.tif]

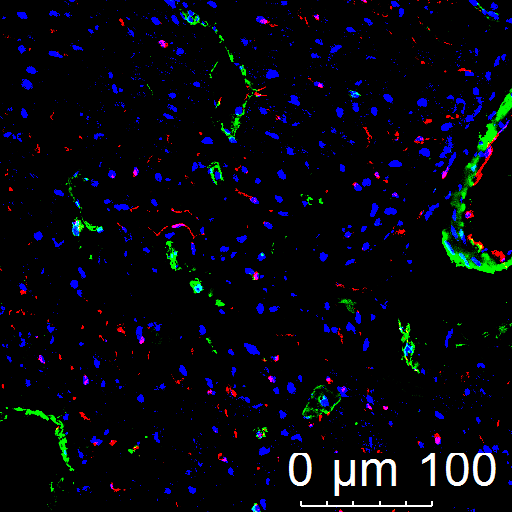

Supplement: Supplementary file 6 — Source data Fig. 4 [file 44321_2026_405_MOESM6_ESM.zip › Figure 4/F/Merge.tif]

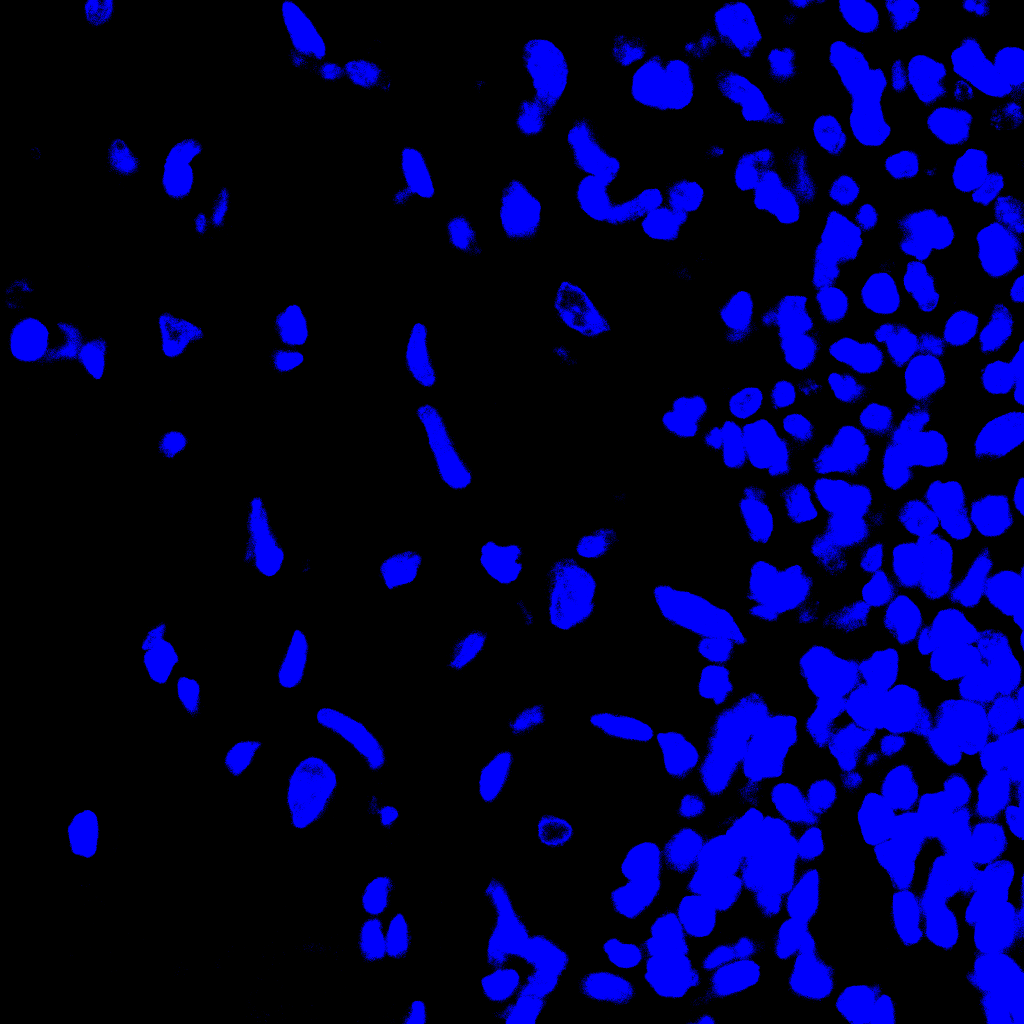

Supplement: Supplementary file 6 — Source data Fig. 4 [file 44321_2026_405_MOESM6_ESM.zip › Figure 4/C/DAPI.tif]

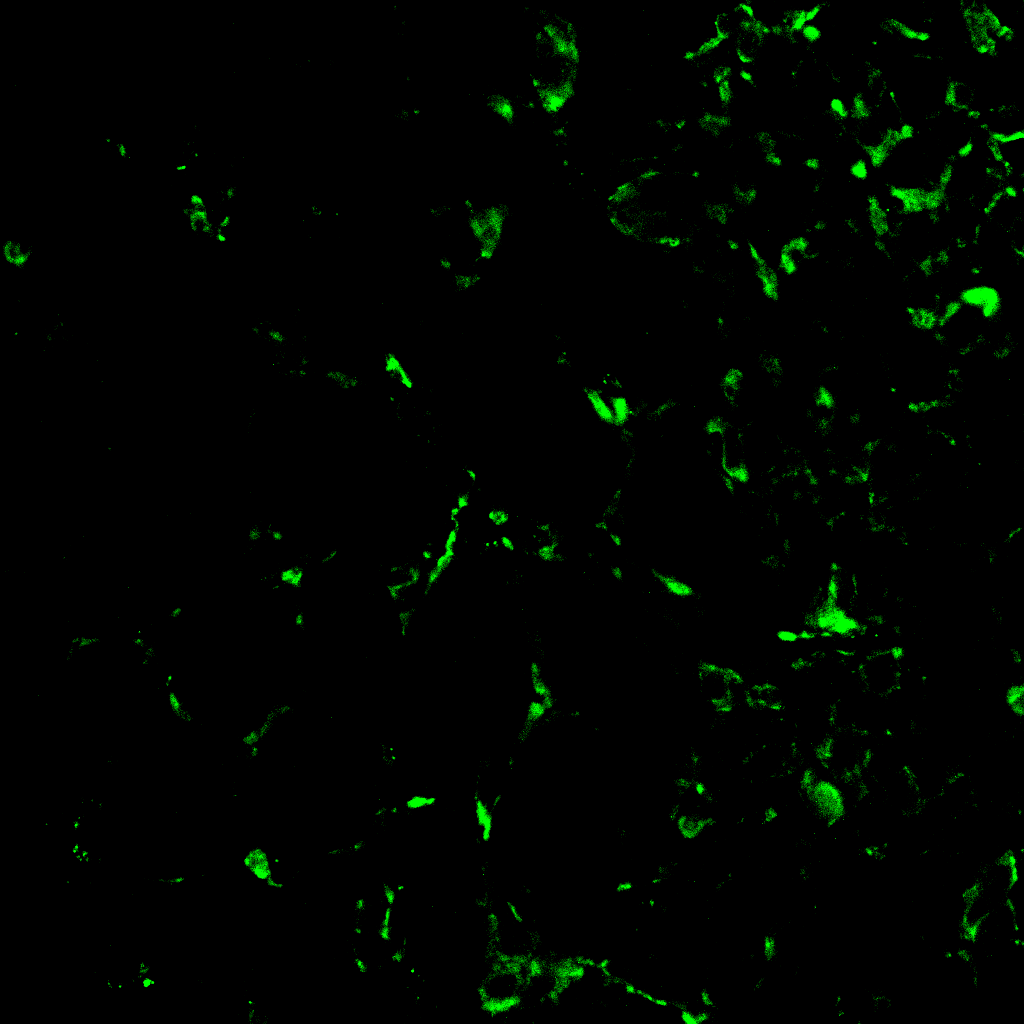

Supplement: Supplementary file 6 — Source data Fig. 4 [file 44321_2026_405_MOESM6_ESM.zip › Figure 4/C/aSMA.tif]

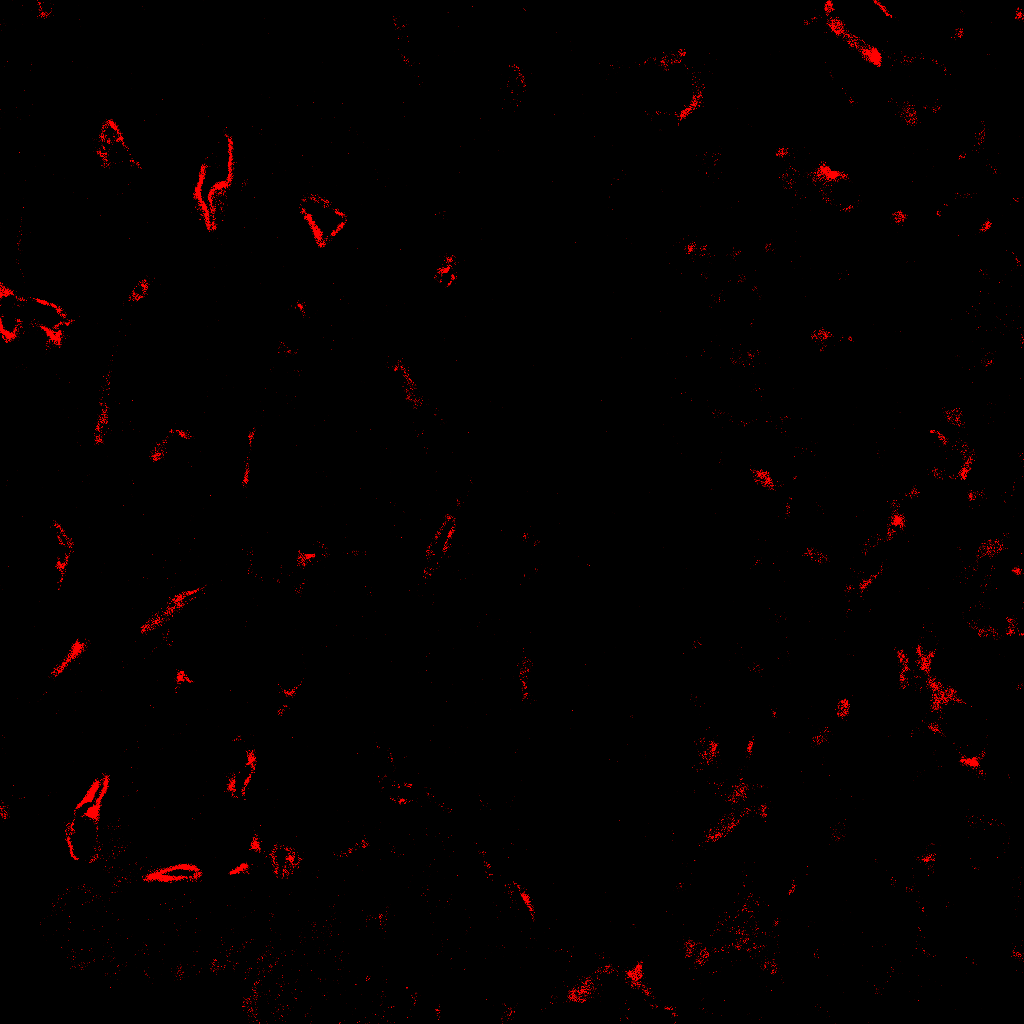

Supplement: Supplementary file 6 — Source data Fig. 4 [file 44321_2026_405_MOESM6_ESM.zip › Figure 4/C/CD31.tif]

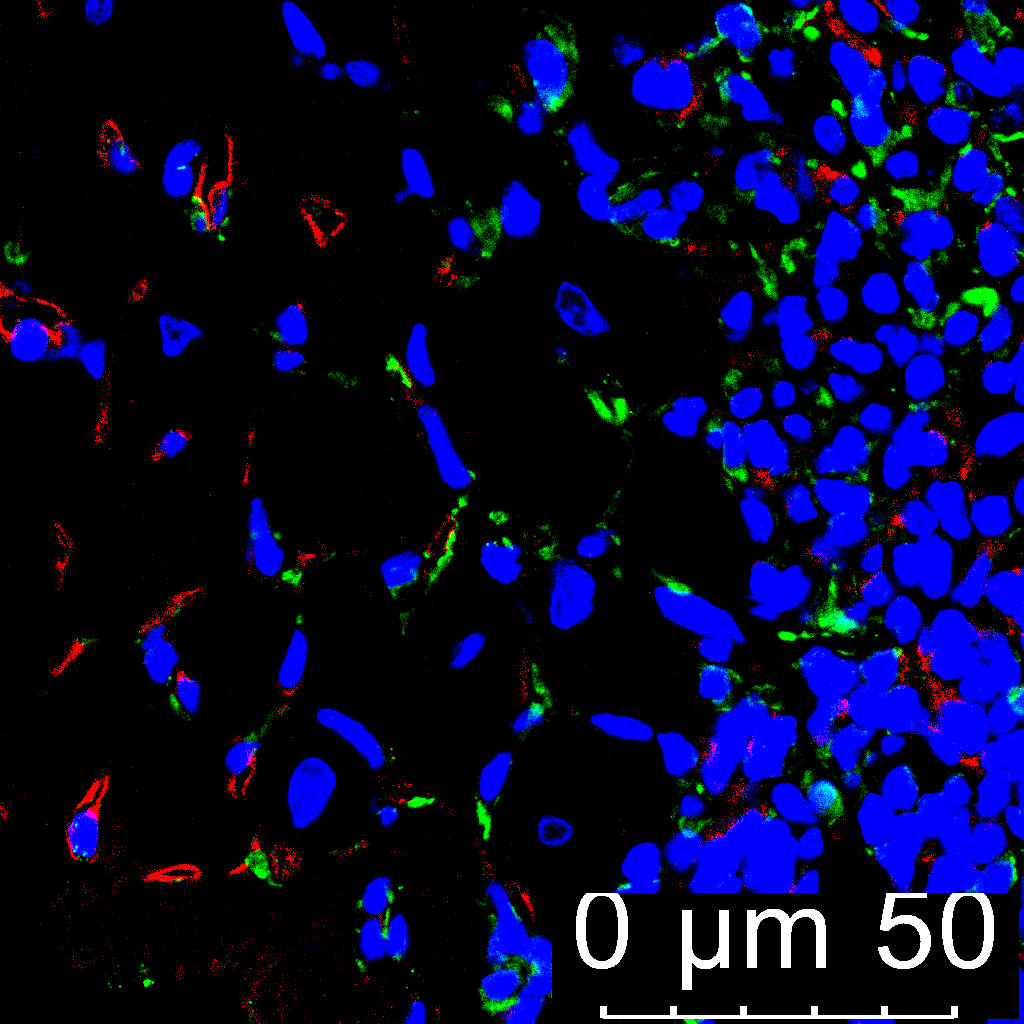

Supplement: Supplementary file 6 — Source data Fig. 4 [file 44321_2026_405_MOESM6_ESM.zip › Figure 4/C/Merge.tif]

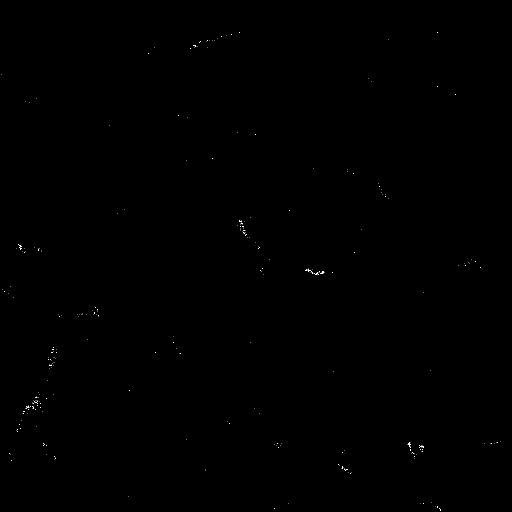

Supplement: Supplementary file 6 — Source data Fig. 4 [file 44321_2026_405_MOESM6_ESM.zip › Figure 4/E/PDGFRa.tif]

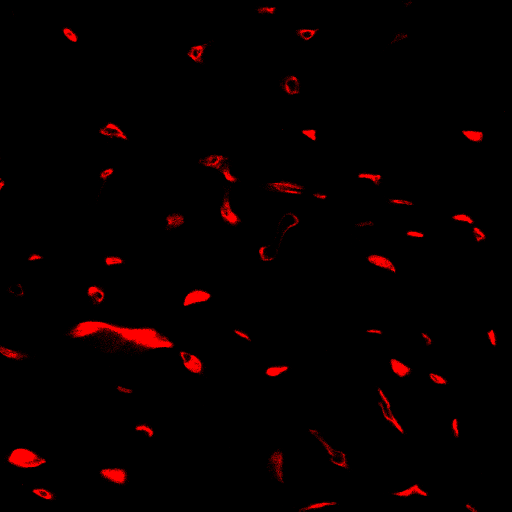

Supplement: Supplementary file 6 — Source data Fig. 4 [file 44321_2026_405_MOESM6_ESM.zip › Figure 4/E/RFP.tif]

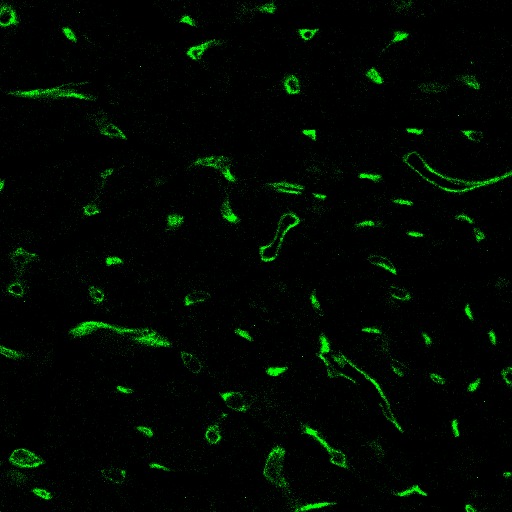

Supplement: Supplementary file 6 — Source data Fig. 4 [file 44321_2026_405_MOESM6_ESM.zip › Figure 4/E/CD31.tif]

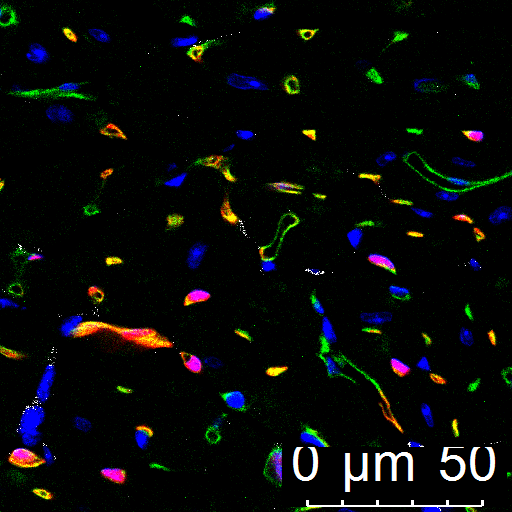

Supplement: Supplementary file 6 — Source data Fig. 4 [file 44321_2026_405_MOESM6_ESM.zip › Figure 4/E/Merge.tif]

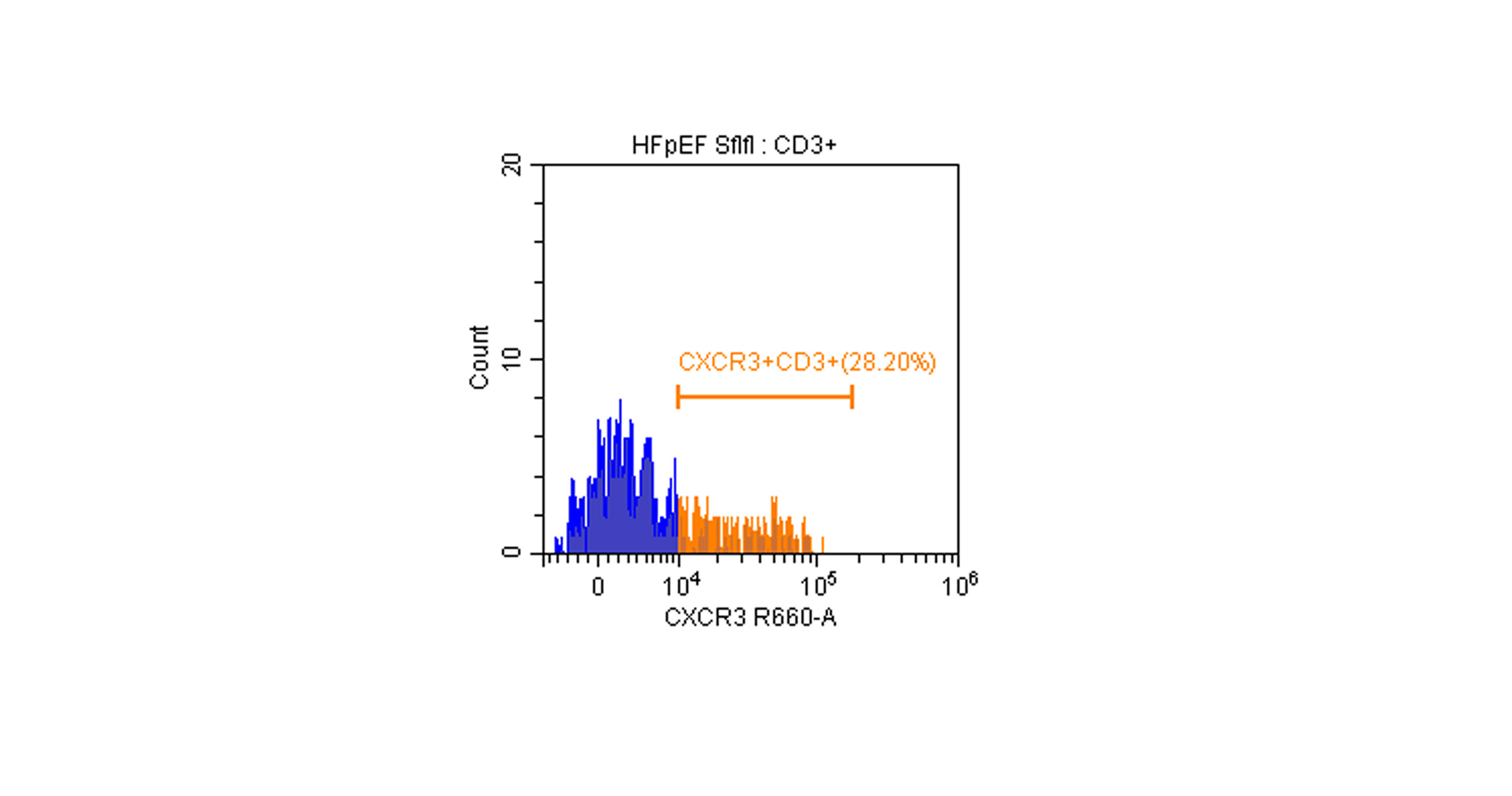

Supplement: Supplementary file 7 — Source data Fig. 5 [file 44321_2026_405_MOESM7_ESM.zip › Figure 5/I/HFpEF Sflfl.jpg]

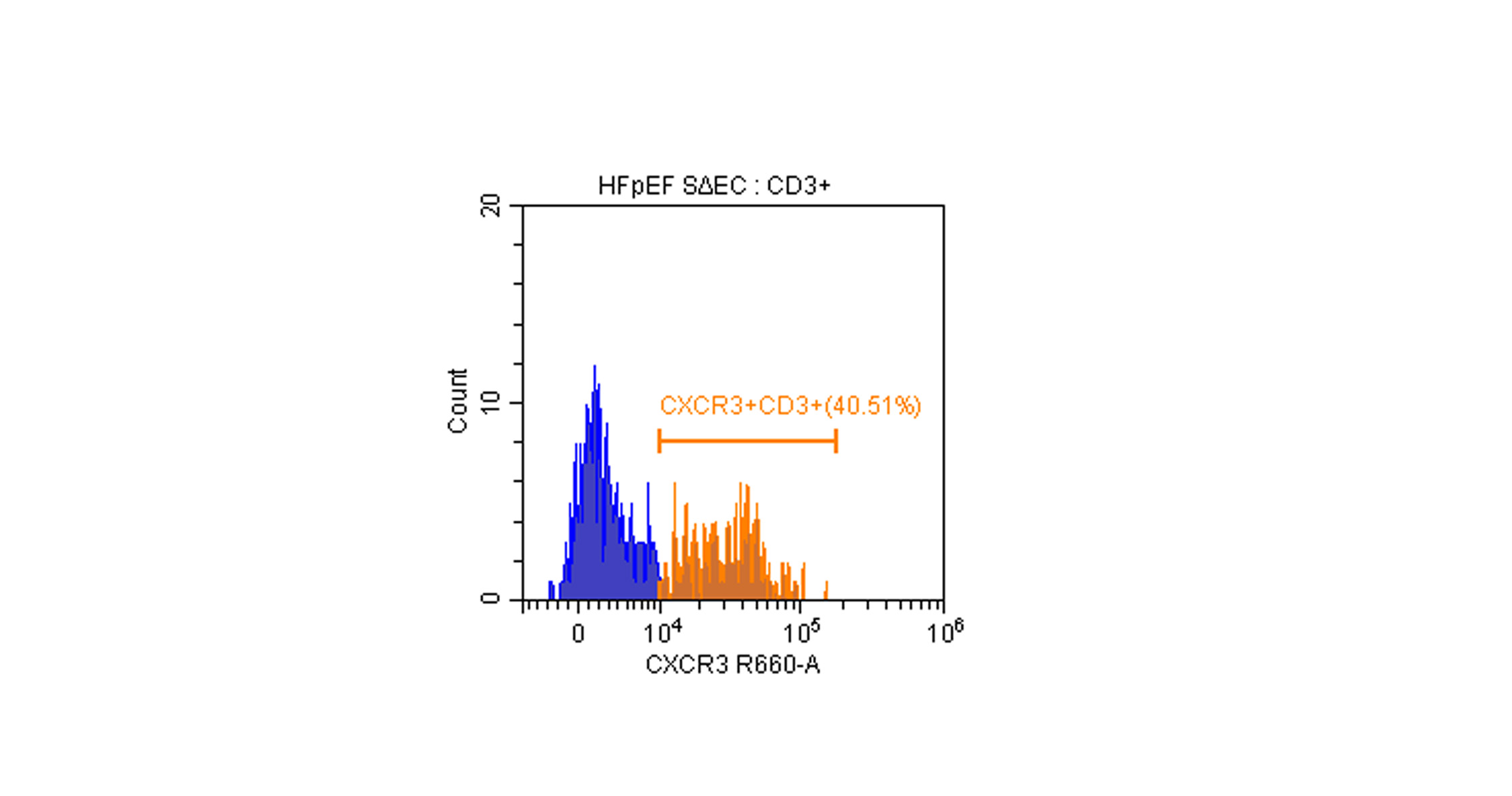

Supplement: Supplementary file 7 — Source data Fig. 5 [file 44321_2026_405_MOESM7_ESM.zip › Figure 5/I/HFpEF SEC.jpg]

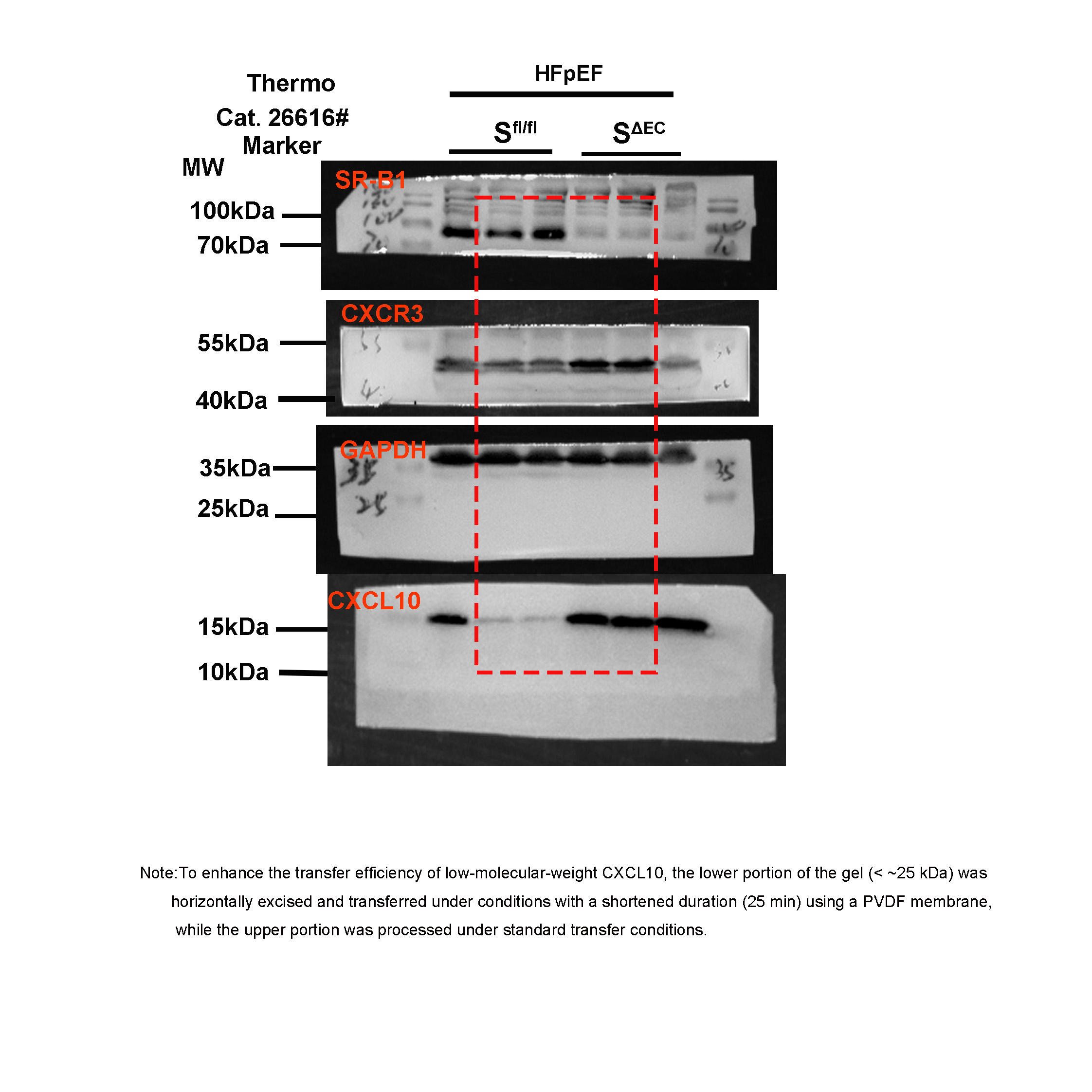

Supplement: Supplementary file 7 — Source data Fig. 5 [file 44321_2026_405_MOESM7_ESM.zip › Figure 5/G/5G.jpg]

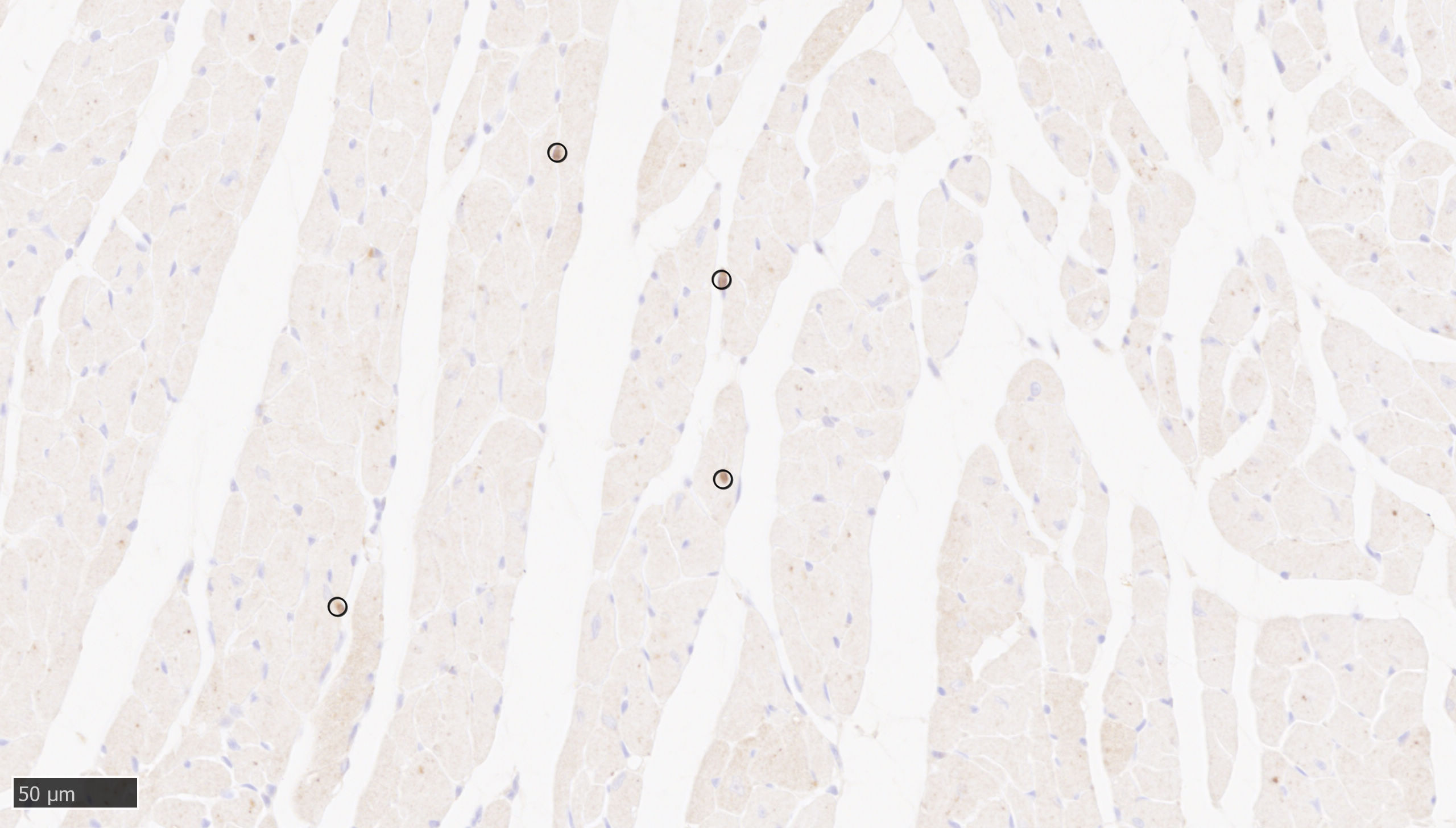

Supplement: Supplementary file 7 — Source data Fig. 5 [file 44321_2026_405_MOESM7_ESM.zip › Figure 5/H/Sflfl zoom-in.jpg]

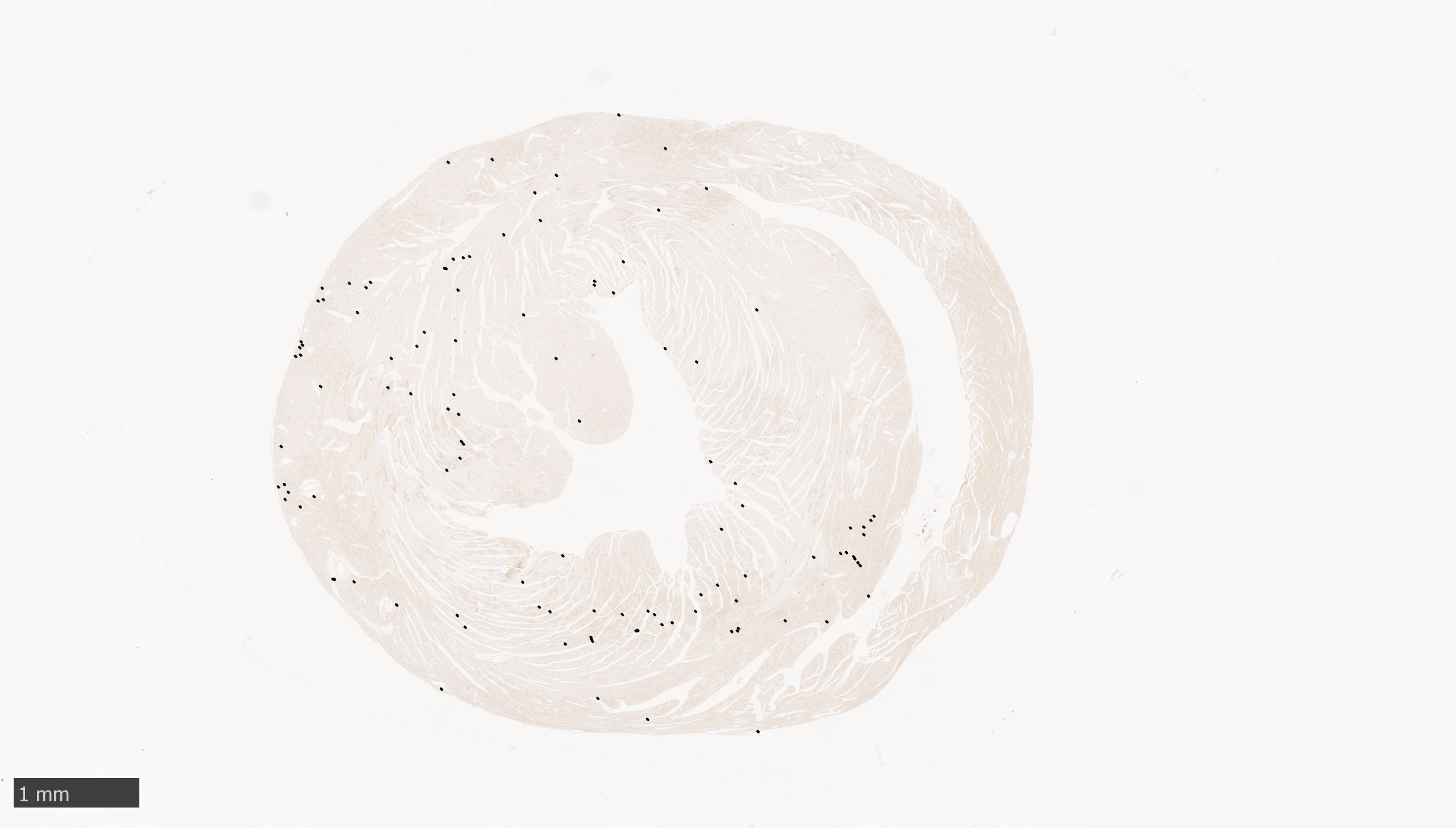

Supplement: Supplementary file 7 — Source data Fig. 5 [file 44321_2026_405_MOESM7_ESM.zip › Figure 5/H/SEC.jpg]

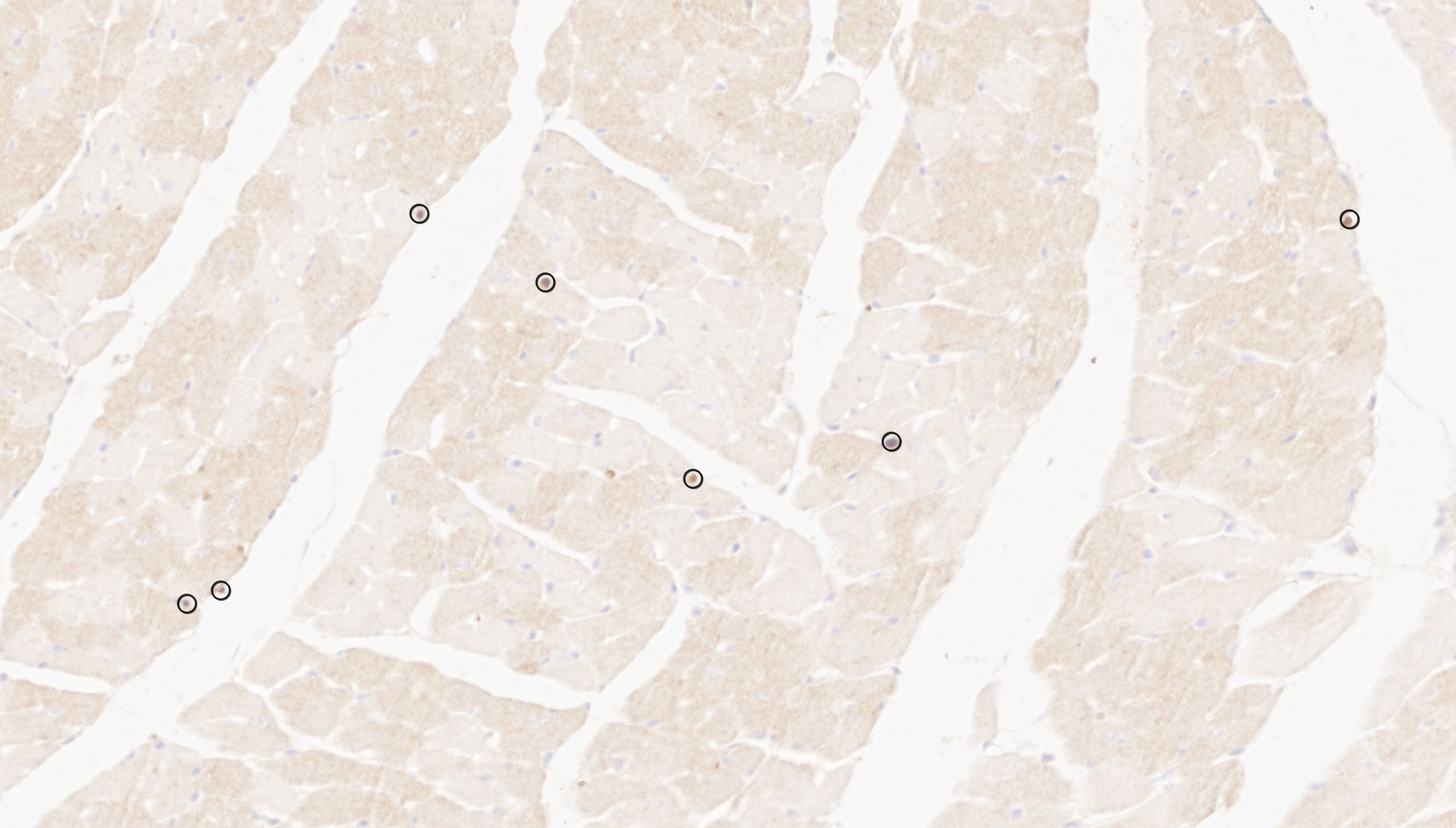

Supplement: Supplementary file 7 — Source data Fig. 5 [file 44321_2026_405_MOESM7_ESM.zip › Figure 5/H/SEC zoom-in.jpg]

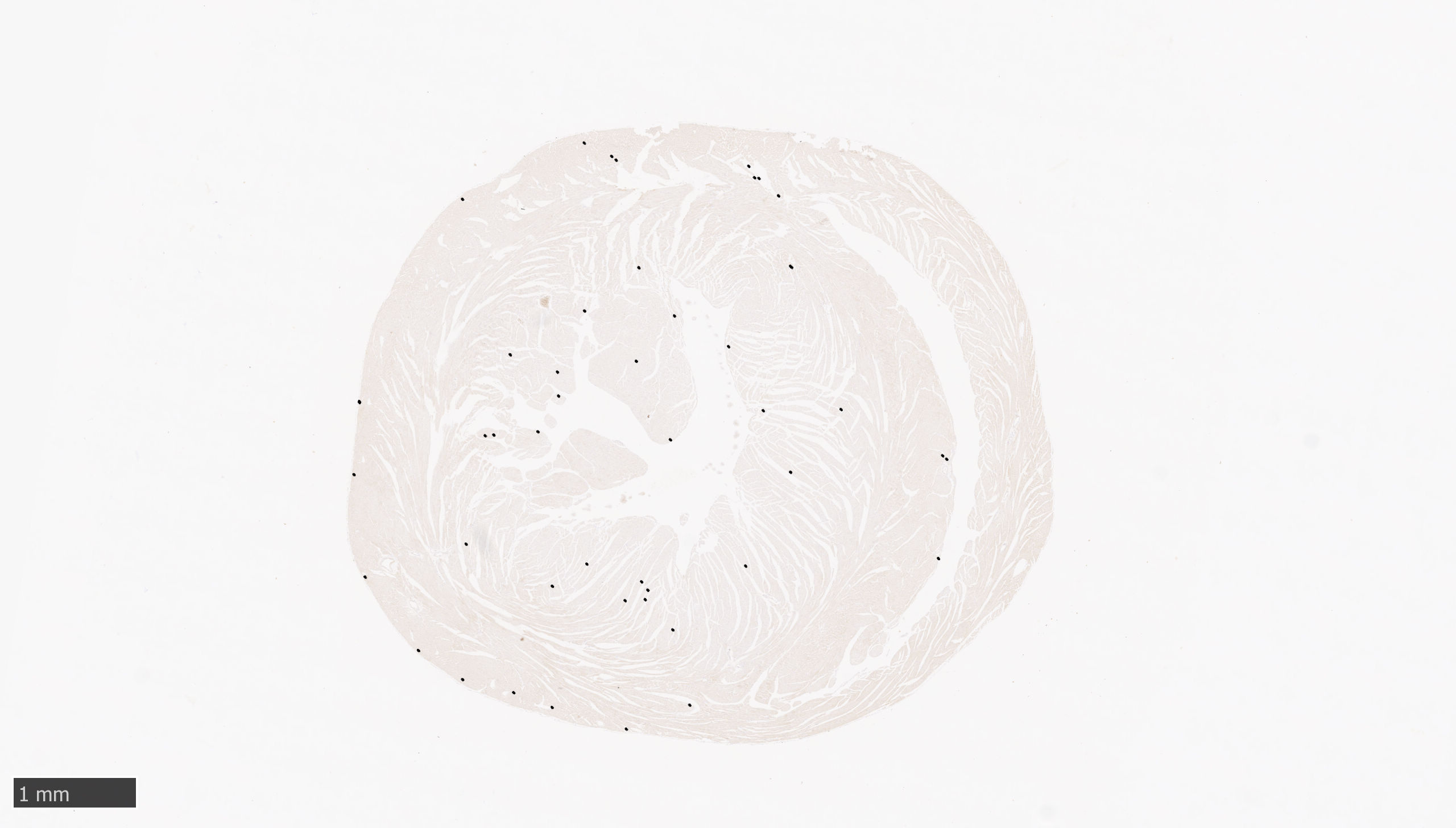

Supplement: Supplementary file 7 — Source data Fig. 5 [file 44321_2026_405_MOESM7_ESM.zip › Figure 5/H/Sflfl.jpg]

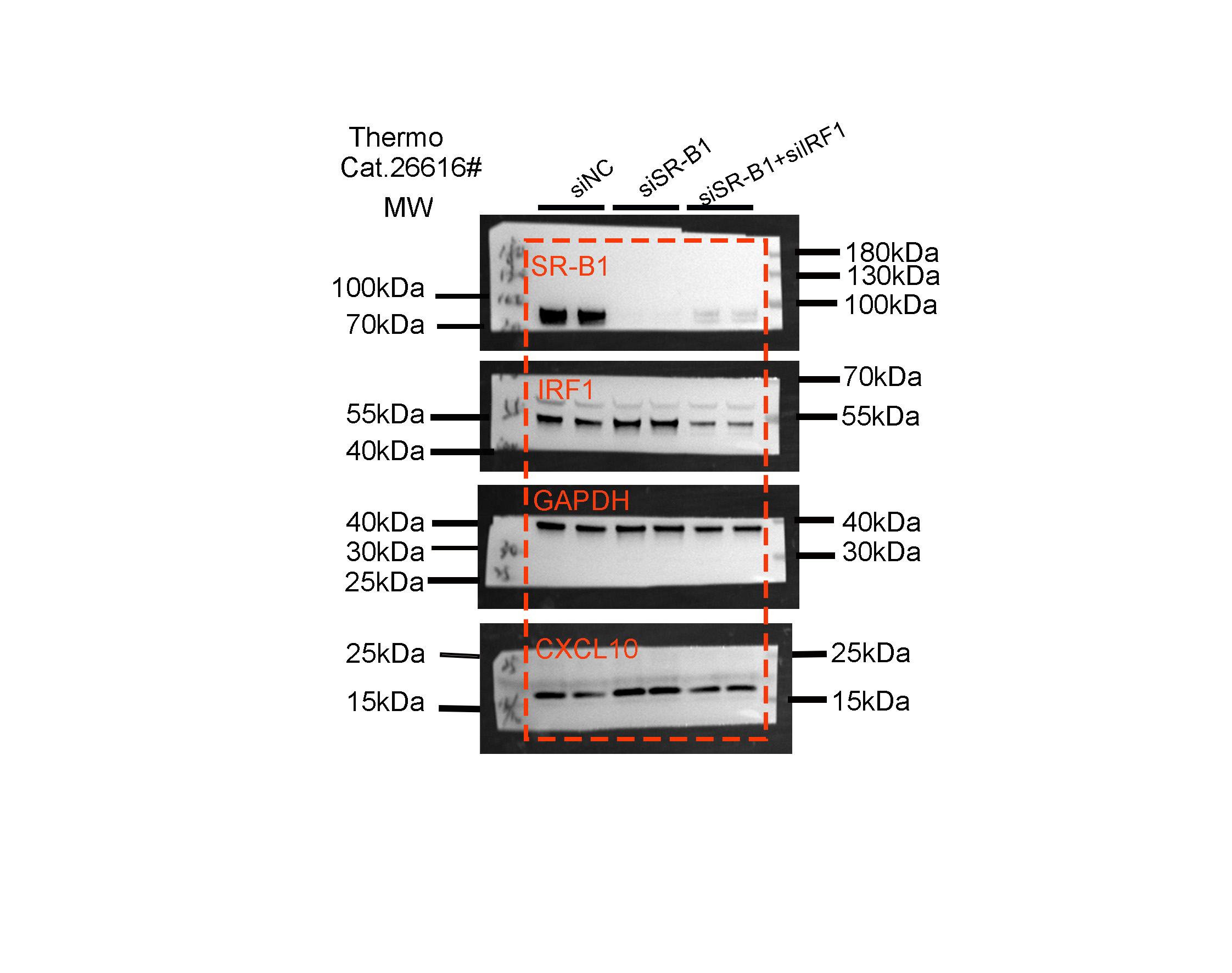

Supplement: Supplementary file 8 — Source data Fig. 6 [file 44321_2026_405_MOESM8_ESM.zip › Figure 6/I/6I.jpg]

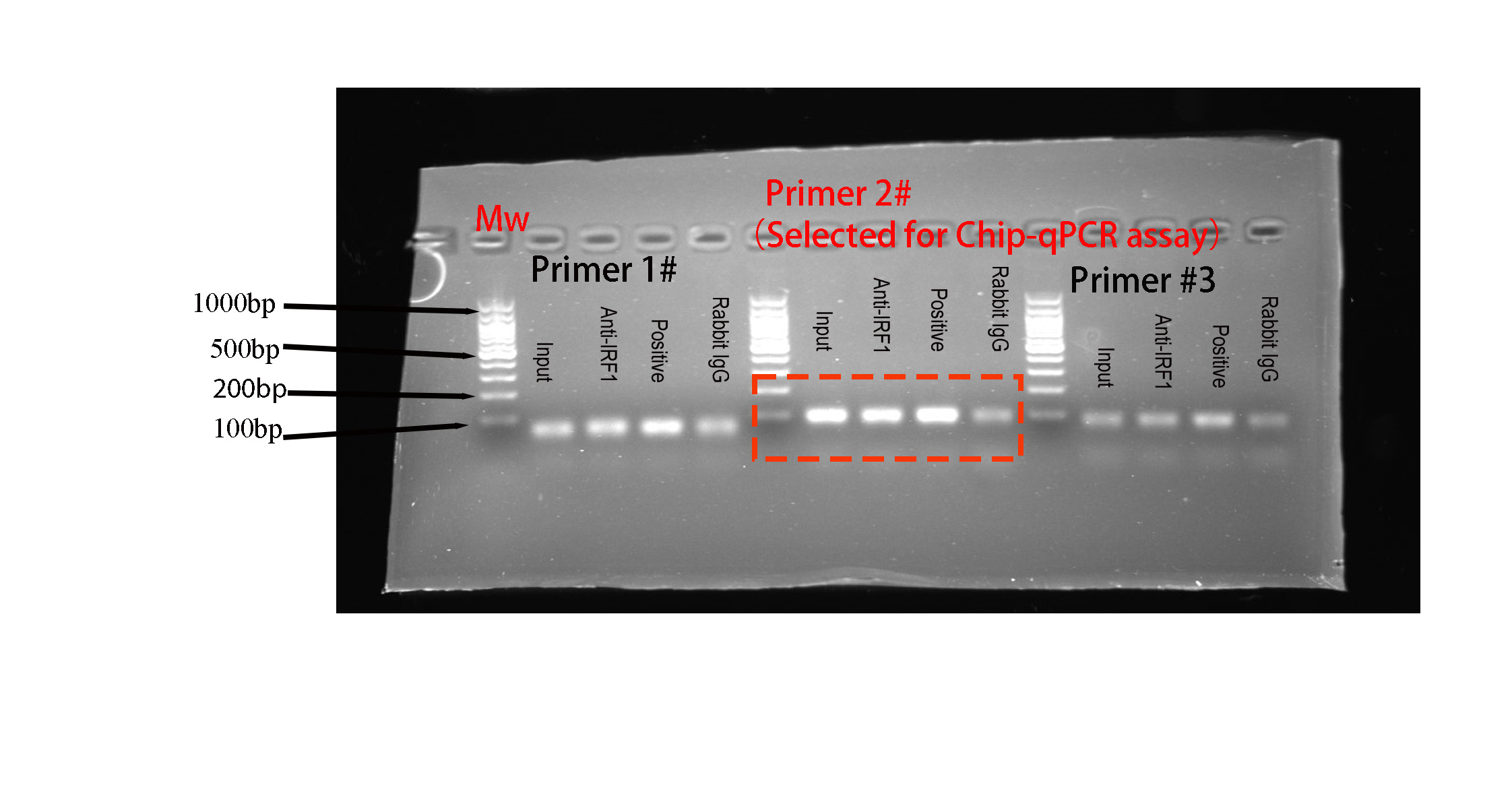

Supplement: Supplementary file 8 — Source data Fig. 6 [file 44321_2026_405_MOESM8_ESM.zip › Figure 6/F/6F.jpg]

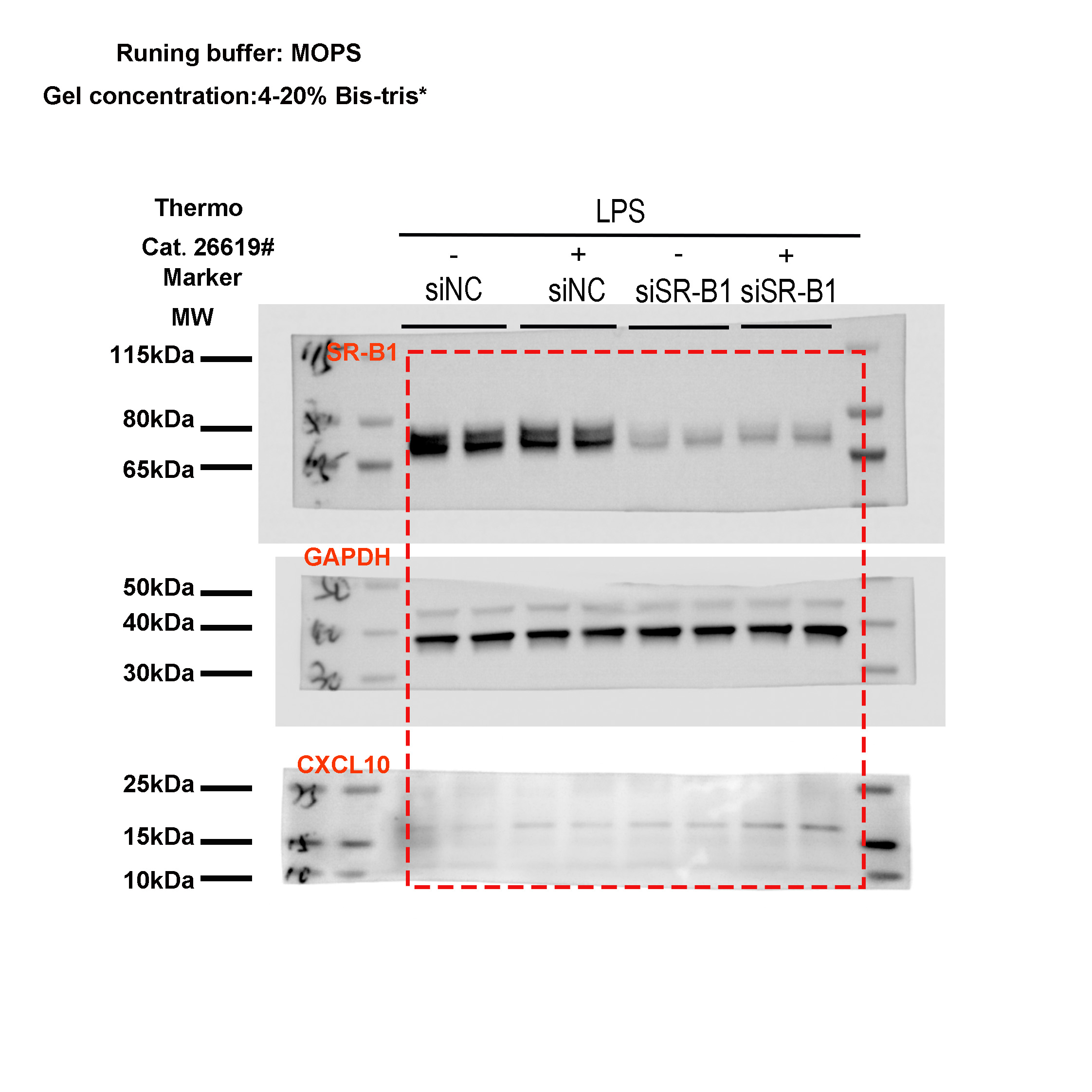

Supplement: Supplementary file 8 — Source data Fig. 6 [file 44321_2026_405_MOESM8_ESM.zip › Figure 6/C/6C.jpg]

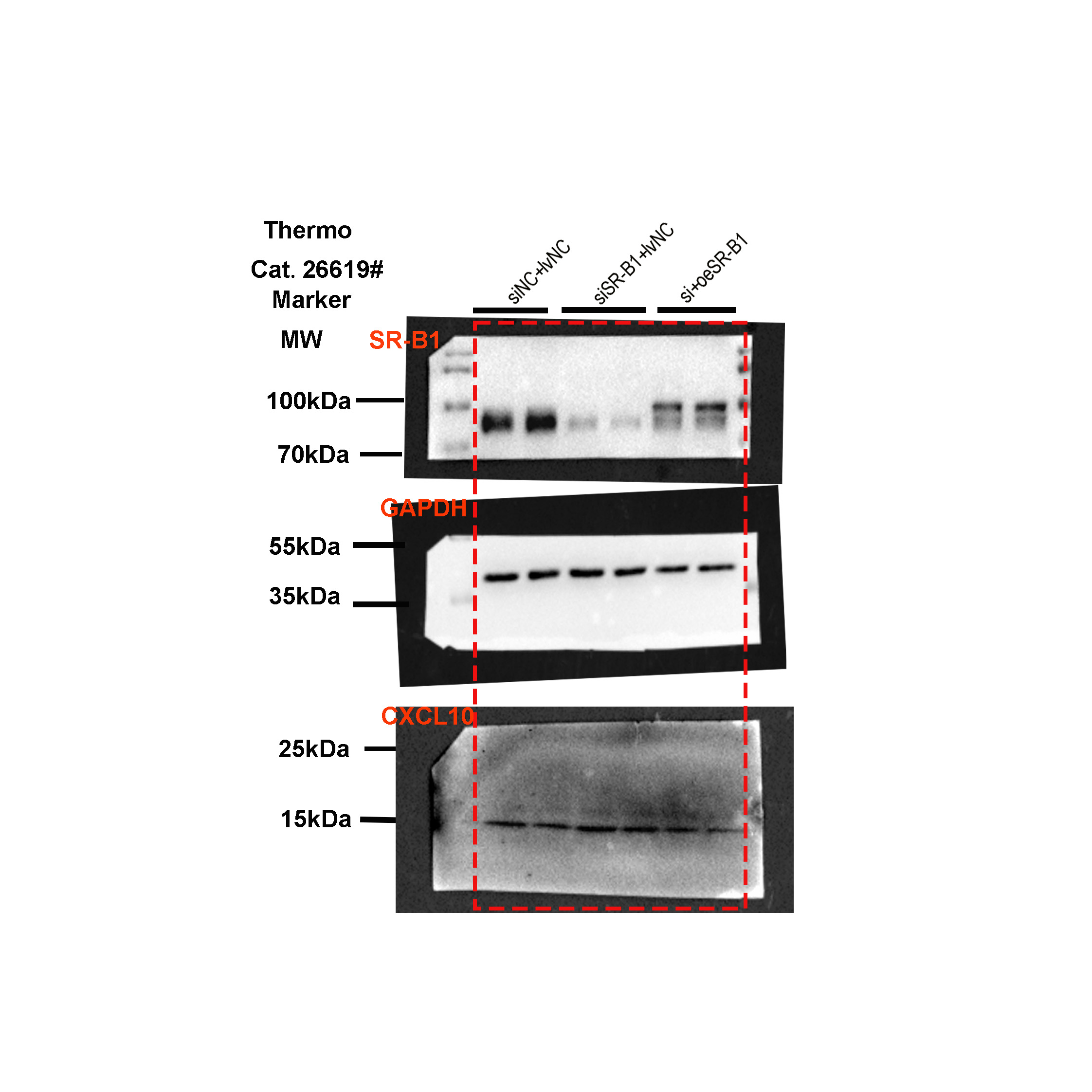

Supplement: Supplementary file 8 — Source data Fig. 6 [file 44321_2026_405_MOESM8_ESM.zip › Figure 6/D/6D.jpg]

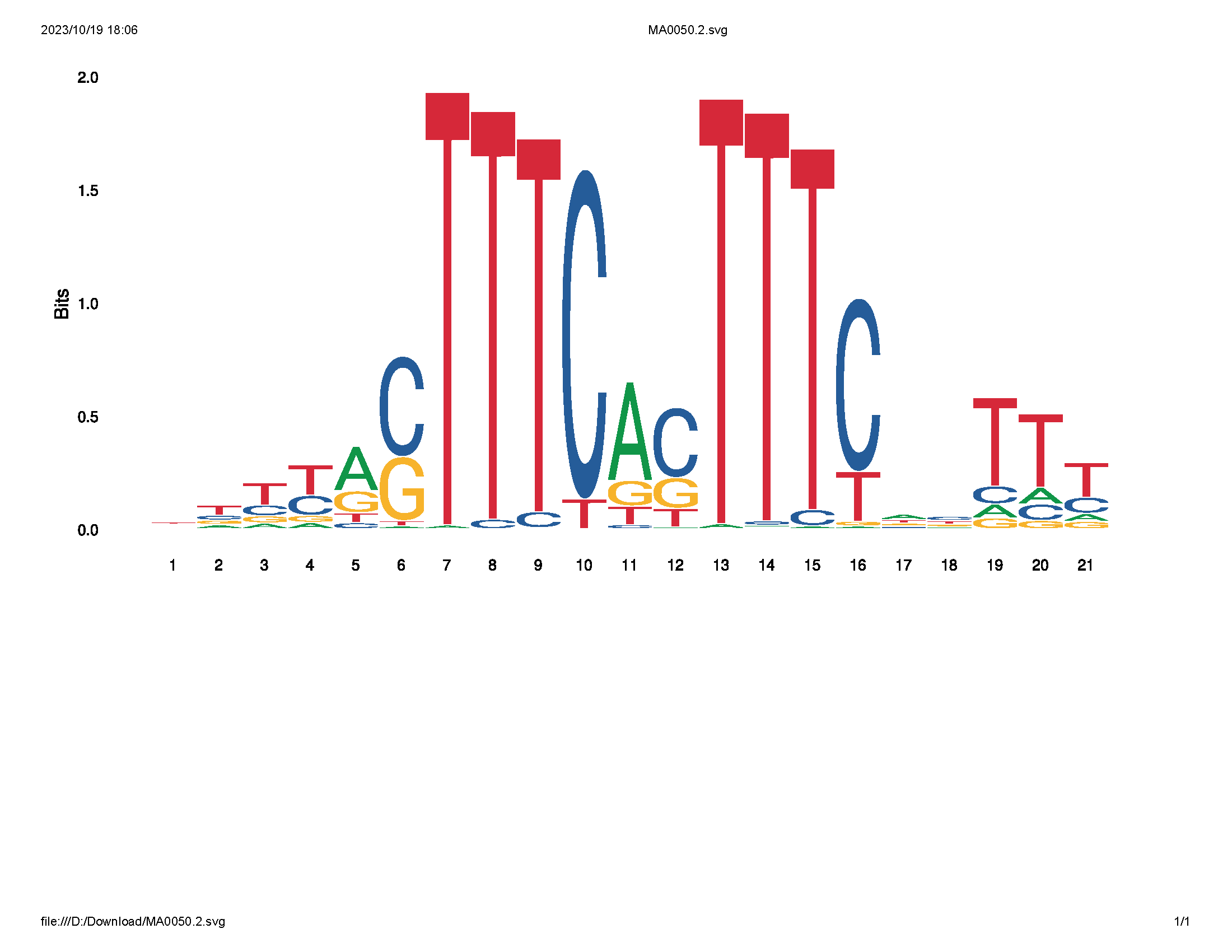

Supplement: Supplementary file 8 — Source data Fig. 6 [file 44321_2026_405_MOESM8_ESM.zip › Figure 6/E/E.tiff]

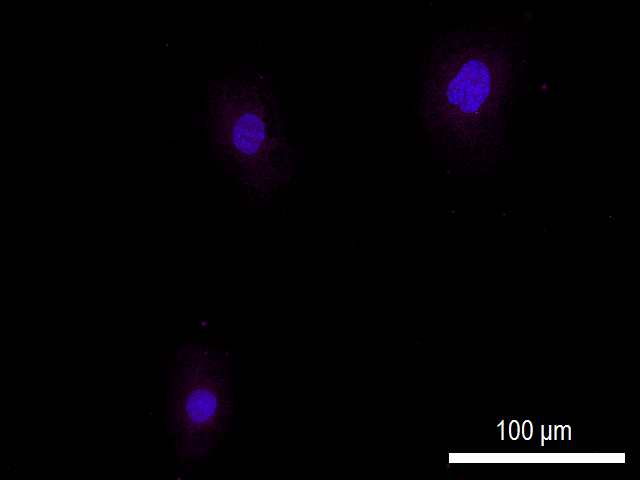

Supplement: Supplementary file 8 — Source data Fig. 6 [file 44321_2026_405_MOESM8_ESM.zip › Figure 6/H/siNC/Merge with Scale.tif]

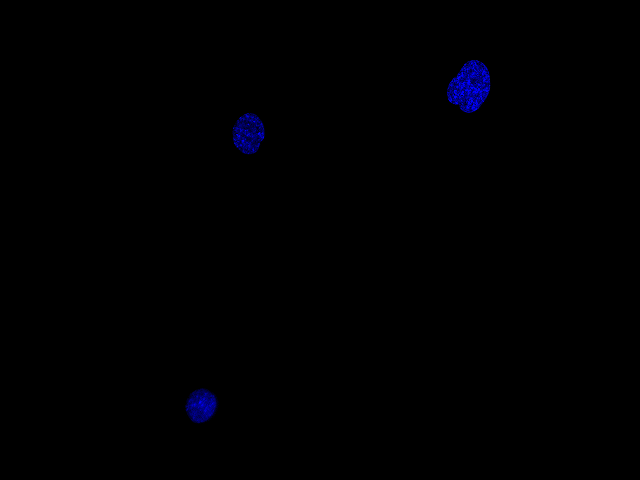

Supplement: Supplementary file 8 — Source data Fig. 6 [file 44321_2026_405_MOESM8_ESM.zip › Figure 6/H/siNC/DAPI.tif]

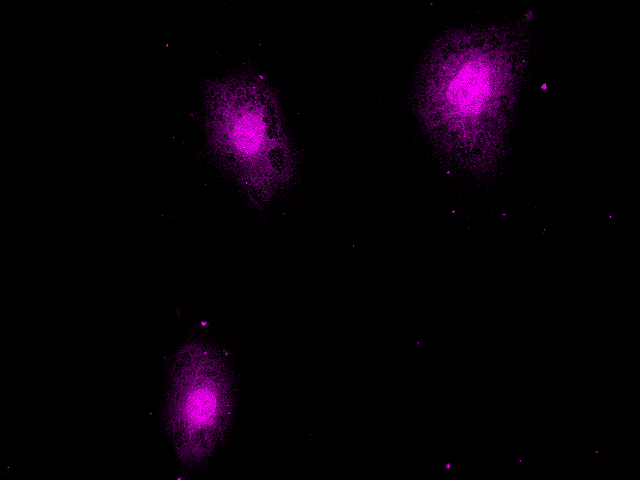

Supplement: Supplementary file 8 — Source data Fig. 6 [file 44321_2026_405_MOESM8_ESM.zip › Figure 6/H/siNC/IRF1.tif]

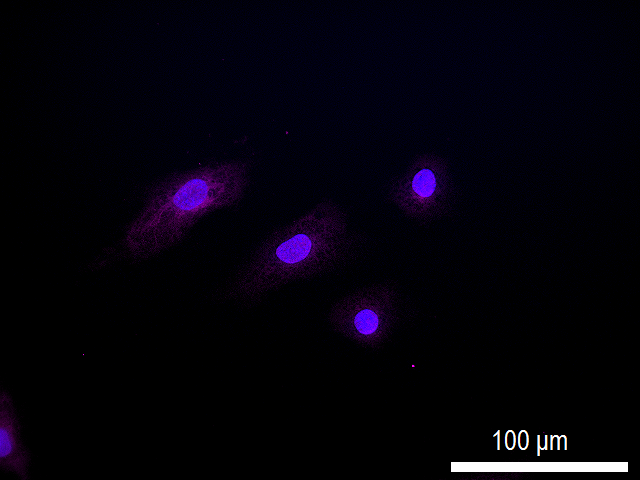

Supplement: Supplementary file 8 — Source data Fig. 6 [file 44321_2026_405_MOESM8_ESM.zip › Figure 6/H/siSR-B1_2/Merge with Scale.tif]

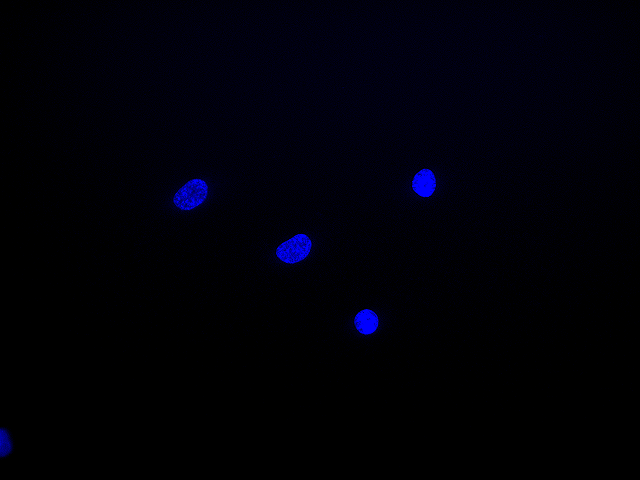

Supplement: Supplementary file 8 — Source data Fig. 6 [file 44321_2026_405_MOESM8_ESM.zip › Figure 6/H/siSR-B1_2/DAPI.tif]

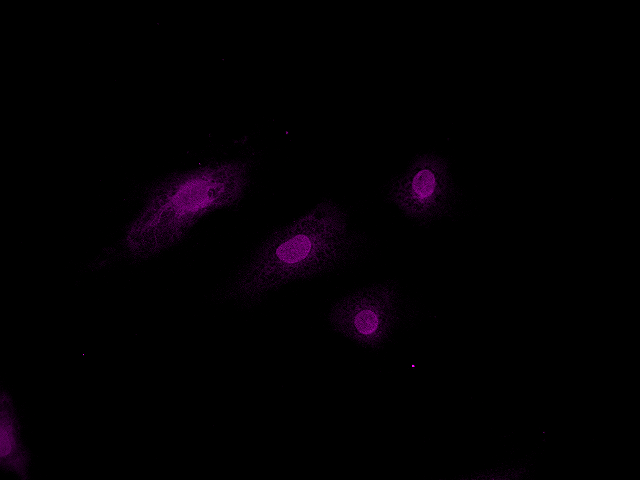

Supplement: Supplementary file 8 — Source data Fig. 6 [file 44321_2026_405_MOESM8_ESM.zip › Figure 6/H/siSR-B1_2/IRF1.tif]

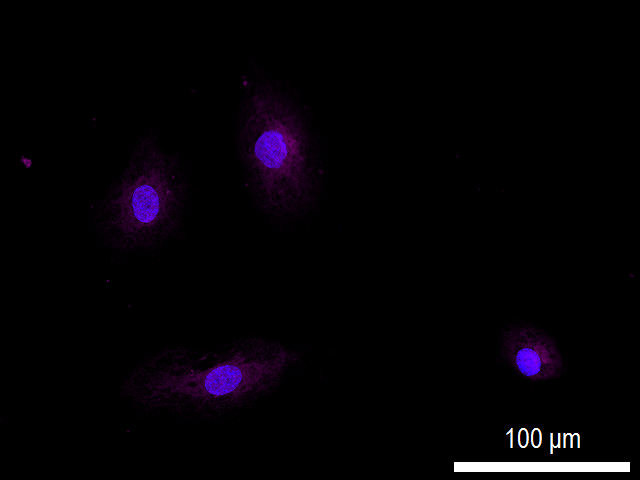

Supplement: Supplementary file 8 — Source data Fig. 6 [file 44321_2026_405_MOESM8_ESM.zip › Figure 6/H/siSR-B1_1/Merge with Scale.tif]

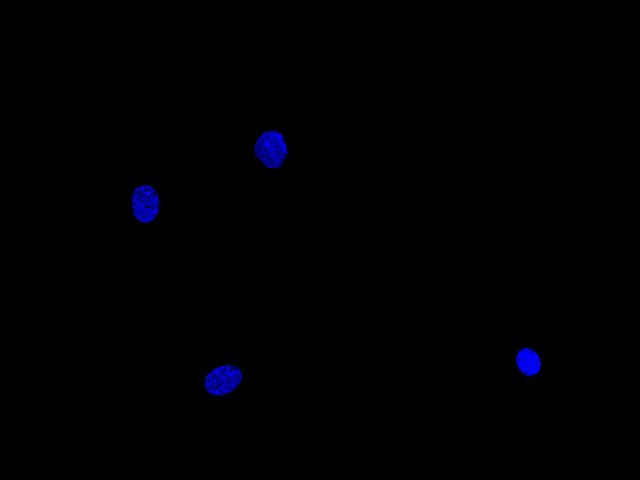

Supplement: Supplementary file 8 — Source data Fig. 6 [file 44321_2026_405_MOESM8_ESM.zip › Figure 6/H/siSR-B1_1/DAPI.tif]

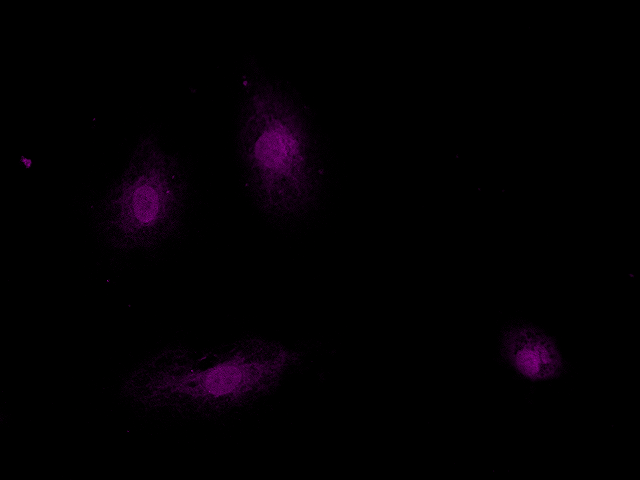

Supplement: Supplementary file 8 — Source data Fig. 6 [file 44321_2026_405_MOESM8_ESM.zip › Figure 6/H/siSR-B1_1/IRF1.tif]

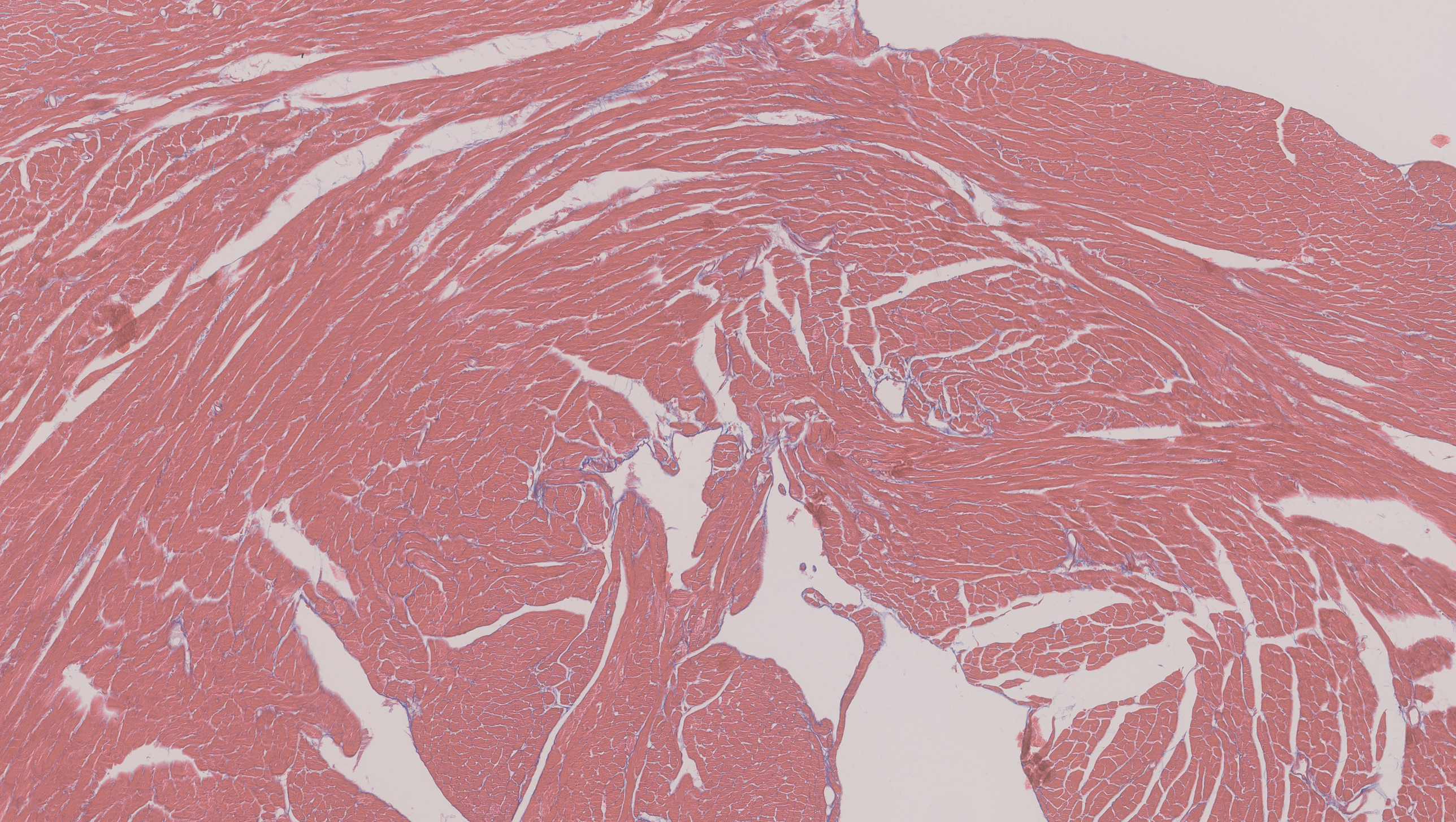

Supplement: Supplementary file 9 — Source data Fig. 7 [file 44321_2026_405_MOESM9_ESM.zip › Figure 7/7L/Masson (zsGreen group).jpg]

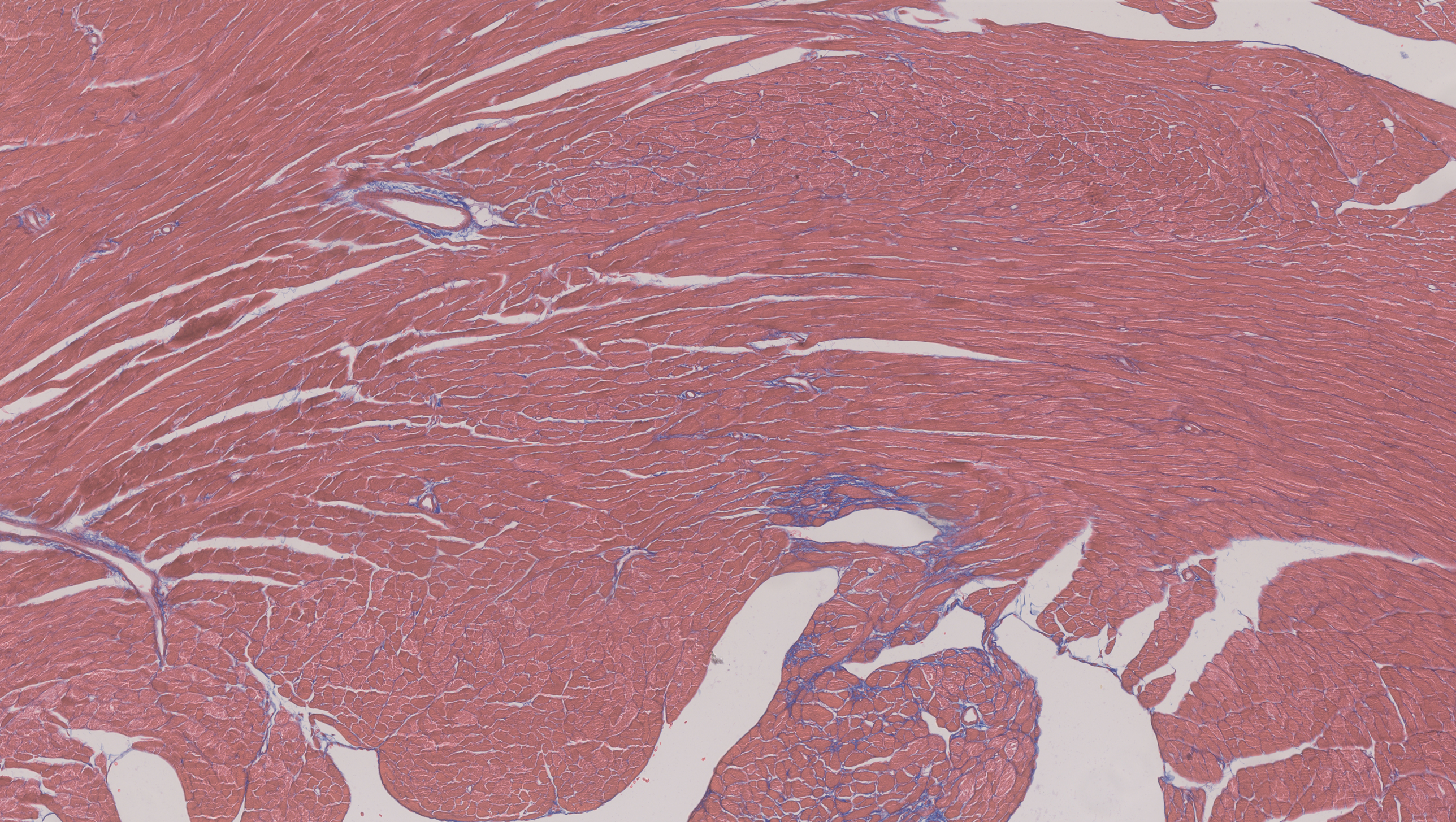

Supplement: Supplementary file 9 — Source data Fig. 7 [file 44321_2026_405_MOESM9_ESM.zip › Figure 7/7L/Masson (CXCL10 group).jpg]

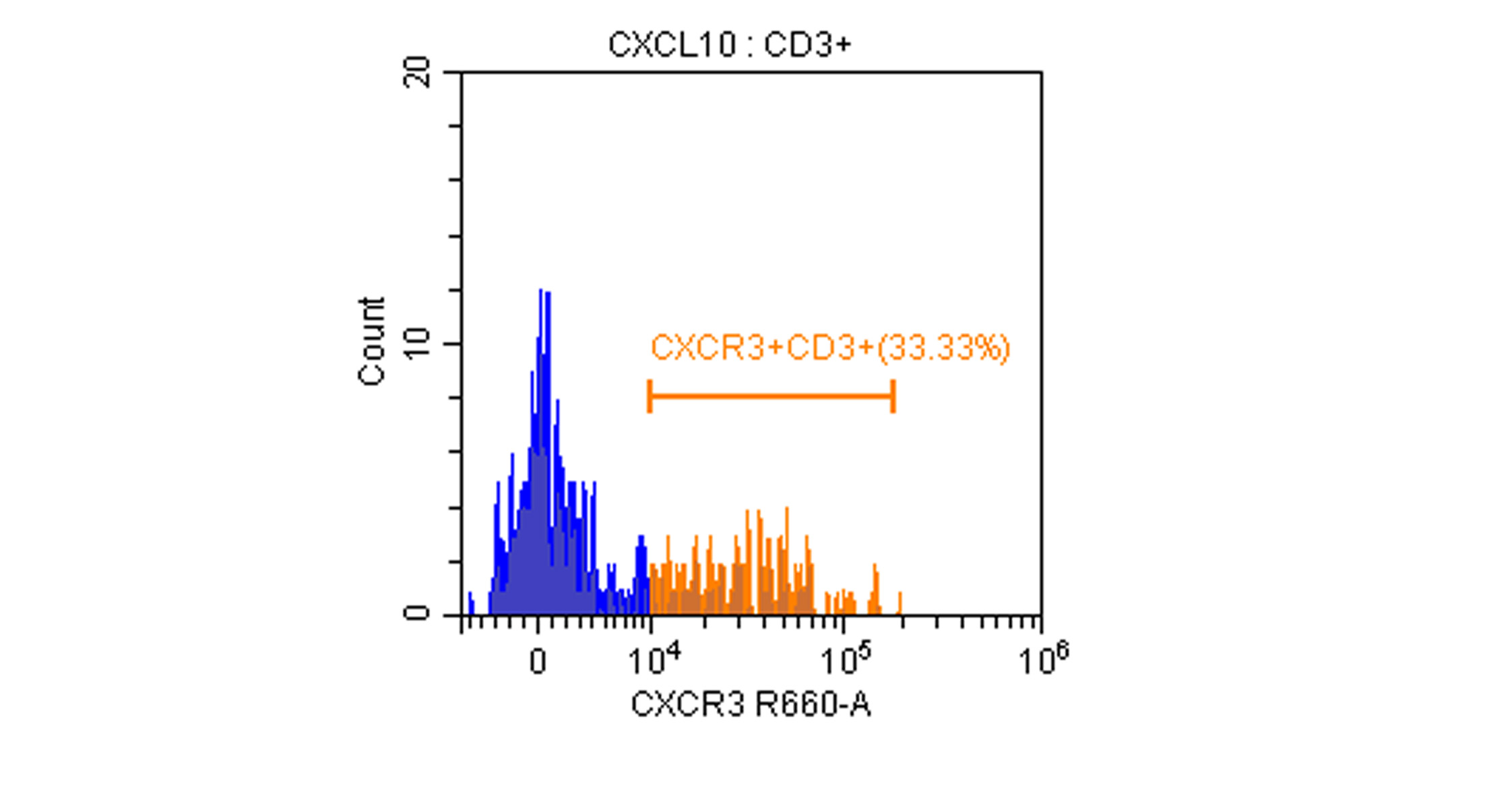

Supplement: Supplementary file 9 — Source data Fig. 7 [file 44321_2026_405_MOESM9_ESM.zip › Figure 7/7P/HFpEF CXCL10.jpg]

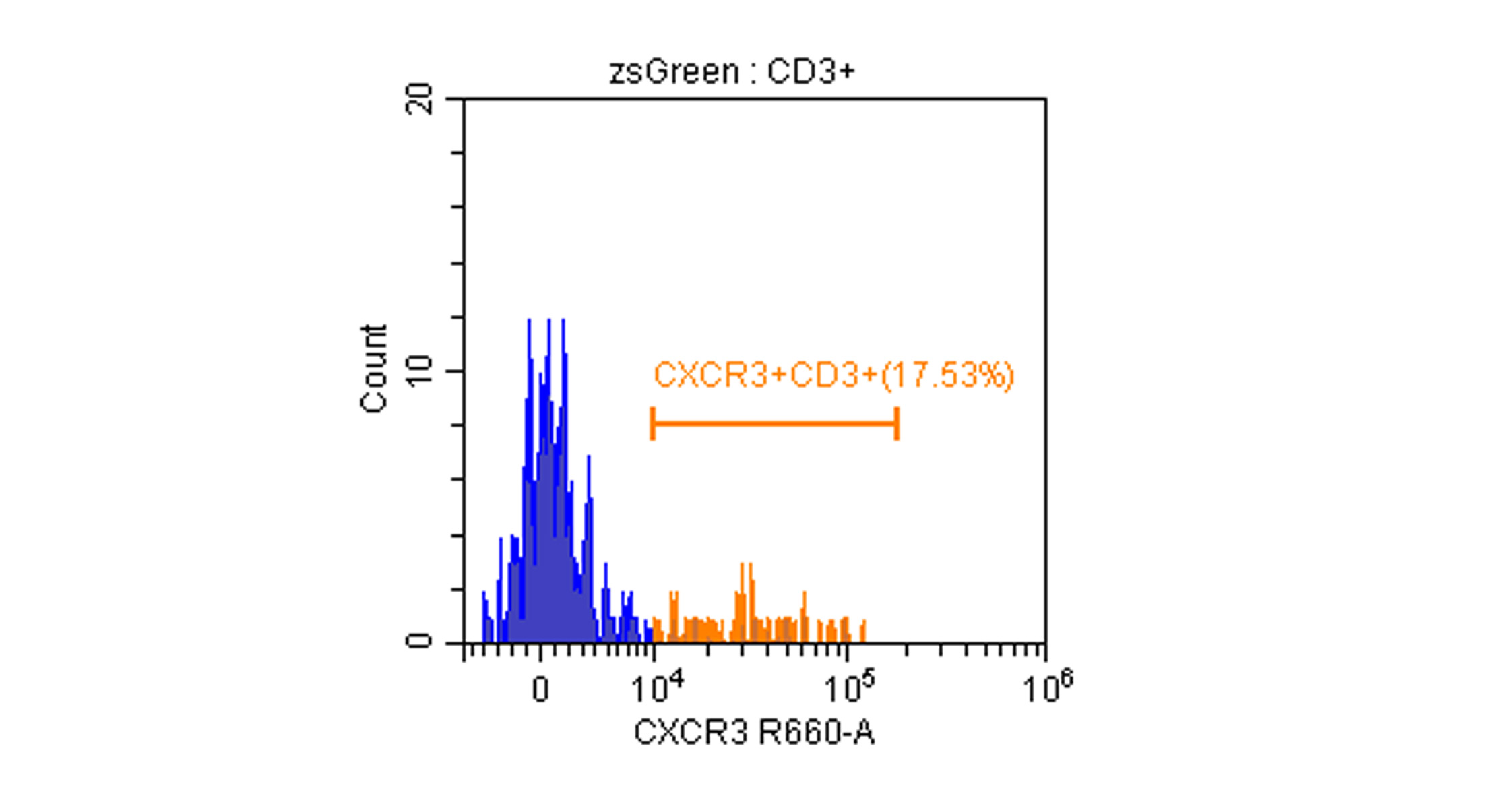

Supplement: Supplementary file 9 — Source data Fig. 7 [file 44321_2026_405_MOESM9_ESM.zip › Figure 7/7P/HFpEF zsGreen.jpg]

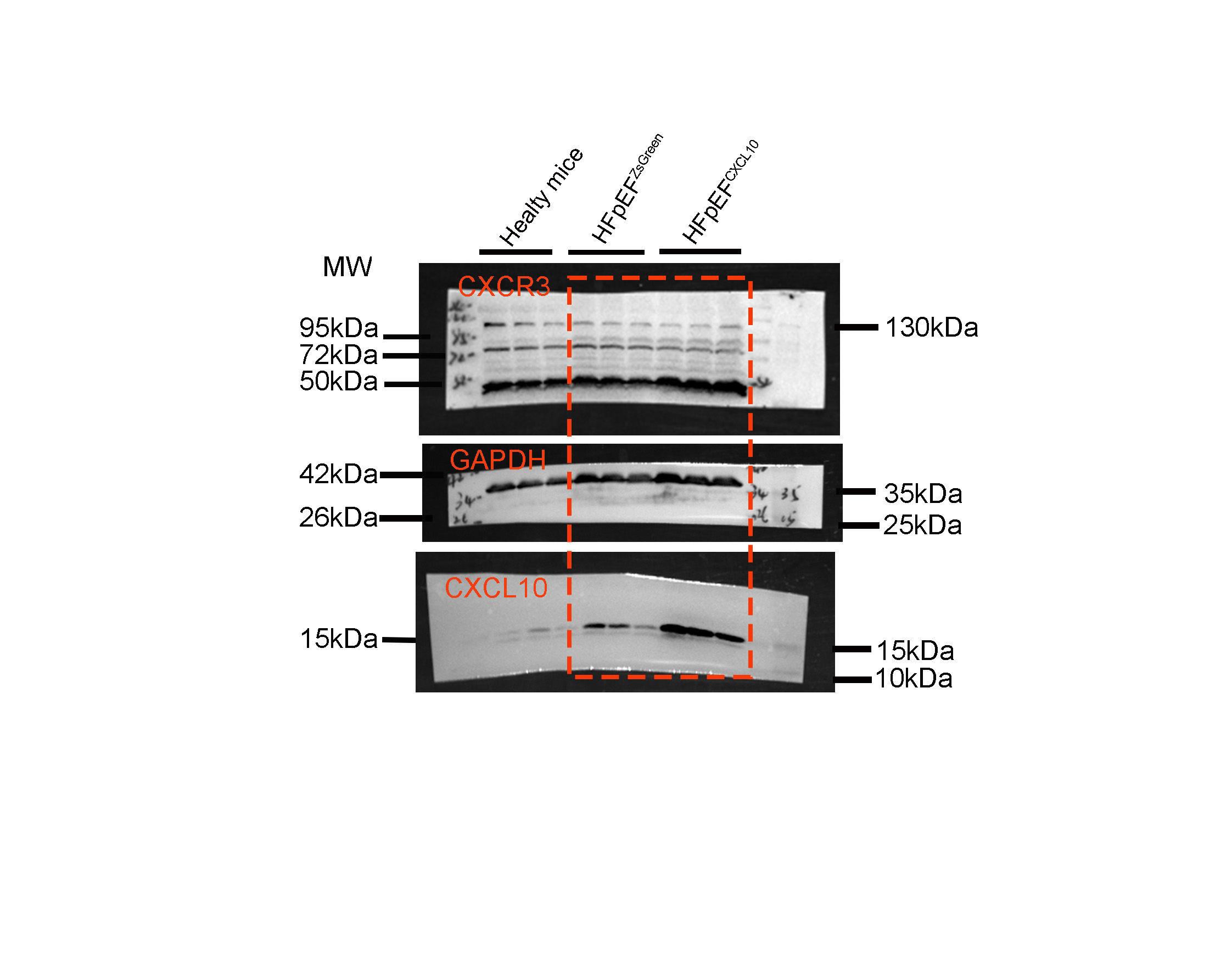

Supplement: Supplementary file 9 — Source data Fig. 7 [file 44321_2026_405_MOESM9_ESM.zip › Figure 7/7O/7O.jpg]
